# A double-blind randomised controlled trial on the effect of Tocovid, a tocotrienol-rich capsule on postoperative atrial fibrillation at the National Heart Institute, Kuala Lumpur: an interim blinded analysis

**DOI:** 10.1186/s13019-021-01721-6

**Published:** 2021-11-24

**Authors:** Ahmad Farouk Musa, Jeswant Dillon, Mohamed Ezani Md Taib, Alwi Mohamed Yunus, Abdul Rais Sanusi, Mohd Nazeri Nordin, Julian A. Smith

**Affiliations:** 1 Jeffrey Cheah School of Medicine and Health Sciences, Monash University Malaysia, Jalan Lagoon Selatan, Bandar Sunway, 47500 Subang Jaya, Selangor Malaysia; 2 Victorian Heart Institute, Monash University, Melbourne, Australia; 3 National Heart Institute, Kuala Lumpur, Malaysia; 4 Department of Surgery, School of Clinical Sciences at Monash Health, Monash University, Melbourne, Australia; 5 Department of Cardiothoracic Surgery, Monash Health, Melbourne, Australia

**Keywords:** Tocovid, Post-operative atrial fibrillation (POAF), CABG, Length of hospital stay (LoHS), Health-related quality of life (HRQoL), Mortality, Morbidity

## Abstract

**Introduction:**

Post-operative atrial fibrillation (POAF) is associated with poorer outcomes, increased resource utilisation, morbidity and mortality. Its pathogenesis is initiated by systemic inflammation and oxidative stress. It is hypothesised that a potent antioxidant and anti-inflammatory agent such as tocotrienol, an isomer of Vitamin E, could reduce or prevent POAF.

**Aims:**

The aim of this study is to determine whether a potent antioxidative and anti-inflammatory agent, Tocovid, a tocotrienol-rich capsule, could reduce the incidence of POAF and affect the mortality and morbidity as well as the duration of ICU, HDU and hospital stay.

**Methods:**

This study was planned as a prospective, randomised, controlled trial with parallel groups. The control group received placebo containing palm superolein while the treatment group received Tocovid capsules. We investigated the incidence of POAF, the length of hospital stay after surgery and the health-related quality of life.

**Results:**

Recruitment commenced in January 2019 but the preliminary results were unblinded as the study is still ongoing. Two-hundred and two patients have been recruited out of a target sample size of 250 as of January 2021. About 75% have completed the study and 6.4% were either lost during follow-up or withdrew; 4% of participants died. The mean age group was 61.44 ± 7.30 years with no statistical difference between the groups, with males having a preponderance for AF. The incidence of POAF was 24.36% and the mean time for developing POAF was 55.38 ± 29.9 h post-CABG. Obesity was not a predictive factor. No statistically significant difference was observed when comparing left atrial size, NYHA class, ejection fraction and the premorbid history. The mean cross-clamp time was 71 ± 34 min and the mean bypass time was 95 ± 46 min, with no difference between groups. There was a threefold increase in death among patients with POAF (*p* = 0.008) and an increase in the duration of ICU stay (*p* = 0.01), the total duration of hospital stay (*p* = 0.04) and reintubation (*p* = 0.045).

**Conclusion:**

A relatively low incidence rate of POAF was noted although the study is still ongoing. It remains to be seen if our prophylactic intervention using Tocovid would effectively reduce the incidence of POAF.

*Clinical Registration Number*: US National Library of Medicine. Clinical Trials - NCT03807037. Registered on 16th January 2019. Link: https://clinicaltrials.gov/ct2/show/NCT03807037

**Supplementary Information:**

The online version contains supplementary material available at 10.1186/s13019-021-01721-6.

## Introduction

Post-operative Atrial fibrillation (POAF) following cardiac surgery is the most common arrhythmia, occurring in about 25% of patients after isolated CABG but can double to between 40 and 50% in combined CABG and valve surgery [1]. In our own retrospective study [2] conducted on post-CABG patients at the National Heart Institute (IJN), Kuala Lumpur, it was demonstrated that patients who developed POAF had a prolonged Intensive Care Unit (ICU) stay, High Dependency Unit (HDU) stay and total hospital stay, with a concomitant consumption of healthcare resources. There was also a six-fold surge in strokes and a three-fold increase in deaths during our study [2].

The almost stagnant incidence of POAF over the years was observed despite the advances in surgical techniques and peri-operative medical care. Various factors have been postulated for the initiation and evolution POAF but it is now thought that shed mediastinal blood that created a proinflammatory and pro-oxidative milieu within the cardiac space played a role in the initiation of POAF [3–5] in light of the fact that POAF has resulted in longer hospital stay with associated increase in morbidity and mortality [6–8], we embarked on a prophylactic strategy to reduce POAF with the hope that it would also able to reduce the cost of hospital care in managing such patients and subsequently reducing the economic burden on the country.

Several studies have been conducted in order to suppress the influence of inflammation since it has been shown to be the main component in the genesis of POAF [3, 4, 9, 10]. But non-pharmacologic efforts using dietary supplements such as fish oil [11] and polyunsaturated fatty acids [12] as prophylactic measures have been shown to be ineffective based upon published meta-analyses and thus far have not been uniformly adopted. However a recent meta-analysis on the known anti-oxidant Vitamin C [13] has shown promising results in reducing POAF along with a shorter ICU and hospital stay. This has encouraged us to use a more potent anti-oxidant and anti-inflammatory agent, tocotrienol [14, 15], which is an isomer of Vitamin E as a compound that could potentially reduce the incidence of POAF and shorten the ICU and hospital stay just like its related, Vitamin C, in our current study.

## Aims

To determine whether the intake of tocotrienol-rich capsules, Tocovid, before and immediately following CABG is safe, reduces the incidence of post-operative AF after CABG, shortens the length of hospital stay, and improves the quality of life of patients post-CABG.

## Methods

### Study design

We planned this study as a prospective, randomised, controlled trial with parallel groups. The main goal was to assess the effect of Tocovid on the occurrence of POAF. We recruited all patients admitted for CABG or CABG and Valve surgery. The IJN’s CABG pathway has a 72-h maximum admission time prior to surgery which was deemed adequate for a minimal treatment duration with Tocovid before CABG.

We assigned the eligible patients to one of the two study arms through a computer-generated randomisation list:

1. Control group with standard care plus palm superolein as placebo, or
2. Treatment group with standard care plus Tocovid.

Immediately after randomisation and at least 2 days prior to surgery, we administered either 400 mg Tocovid per day in two divided doses or placebo. Hovid Berhad produces and markets Tocovid as Tocovid Suprabio, with each 200 mg soft-gel capsule containing 61.52 mg alpha-Tocotrienol, 112.80 mg gamma-Tocotrienol, 25.68 mg delta-Tocotrienol and 91.60 IU alpha-tocopherol. We continued this regime for a minimum of 5 days after surgery and until the follow-up visit at 6 weeks after discharge.

We estimated the dosage of Tocovid based on the regime used by Olaf Stanger et al. [16] which consisted of three ampoules of 45 IE Vitamin E; this is equivalent to 30 mg per ampoule or 90 mg in total. Since the oral preparation of Tocovid has a lower and incomplete absorption compared to an intravenous (IV) formulation [17], we decided on a higher dosing. In fact, the bioavailability of oral administration can be as low as 10–30% [17]. Given that many other clinical studies used 400 mg daily without any adverse effects [18, 19], we decided on the same dosing.

The treatment was continued until the patient was discharged. Compliance was monitored by the cardiothoracic ward nurses. Blood was taken for tocotrienol level testing pre-operatively on admission: at day-4 post-op, just before discharge and during the first follow-up 6 weeks later—the termination date of the study. After discharge, all patients were asked to report to the outpatient department of our institution in case of any relevant symptoms. ECG was also be taken at follow-up. All POAF episodes were treated under the direction of the attending cardiothoracic surgeon.

**Fig. 1:**
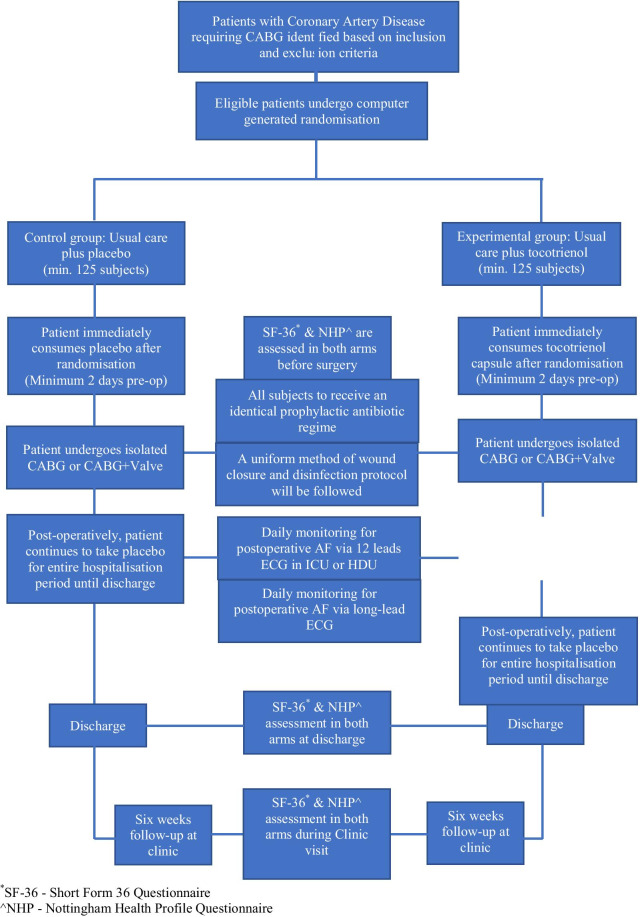
Study flow chart

For the study flow chart, see attachment: Fig. 1

### Inclusion and exclusion criteria

Inclusion Criteria:

Males or females more than 18 years of age.

Elective, on-pump surgery of coronary artery revascularisation, isolated or combined valve surgery.

Exclusion Criteria:

Any urgent or emergency surgery and off-pump surgery.

Poor LV (EF < 30%).

Allergy to palm oil or Vitamin E, or any form of arrhythmia pre-operatively.

Long-term corticosteroid treatment.

Participation in other clinical trial within the previous 3 months.

Supplementation with Vitamin E or other potent antioxidants within 1 month prior to randomisation.

### Study end points

The primary end point was the occurrence of POAF similar to any electrocardiographically confirmed episode of AF/Atrial Flutter (AFL) post CABG of at least 30-s duration. If a shorter duration ECG was available, we diagnosed AF/AFL on the arrhythmia present at onset or termination [20].

The secondary end points are the length of hospital stay (LoHS) post-surgery which include ICU and HDU stay and the health-related quality of life (HRQoL) of patients; the latter will be determined at the end of the study using the validated Malay Short-Form 36 Questionnaire (SF-36) [21, 22] and Nottingham Health Profile Part I [23]. The attending surgeon blinded to treatment assignment adjudicated all the end points based on the clinical records and ECG tracings.

### Sample size calculation

We used the PS Power and Sample Size Calculation Software for sample size calculation.

Calculation of sample size in the present study requires precise specification of the primary hypothesis of the study (Tocovid consumption reduces the incidence of POAF in subjects that had undergone CABG) and the method of analysis (using Relative Risk: RR)*.* In addition, the study has taken into account the possibility of “loss to follow-up” (attrition bias) of subjects by analysing all subjects from the start to completion of the study according to the groups in which they were originally randomised [Intention-To-Treat (ITT) analysis] [24]. To calculate the desired sample size, we used the PS Power and Sample Size Calculation Software [25, 26].

In the present RCT, the estimated sample size for the primary end point (*incidence of POAF*) was computed on the basis of findings from a prior study by Musa et al. [2] who found the incidence of POAF at IJN to be 28.7%. This RCT was planned with experimental subjects and controls with one control(s) per experimental subject similar to the study by Saravanan et al. [27] If the true relative risk of AF for experimental subjects relative to controls is 0.45 [28], then using the PS Power and Sample Size Calculator [25, 26] with α equivalent to 0.05 and power (1 − β) is 0.8, the estimated sample size is 103 experimental subjects and 103 control subjects in order to be able to reject the null hypothesis that this relative risk equals 1 with probability (power) of 0.8. We used the uncorrected chi-squared statistics to evaluate this null hypothesis. Taking into account a possible attrition rate of 20%, the total sample size was: 103 + 0.20 (103) × 2 = 250 subjects of which there will be 125 controls and 125 experimental subjects.

### Statistical analysis

We used SPSS version 27.0 for statistical analysis. A* p*-value of less than 0.05 is considered statistically significant.

## Ethical consideration

The study was conducted in compliance with ethical principles outlined in the Malaysian Good Clinical Practice Guideline; it also abides by the Helsinki Declaration revised in 2013. Written informed consent was obtained from potential study participants and/or legally acceptable representative prior to their enrolment into the study.

Ethical approval was obtained from both the National Heart Institute Research Ethics Committee (IJNREC/201/2017), the Monash University Human Research Ethics Committee (2017-9227-10263) and the National Pharmaceutical Regulatory Agency (NPRA) (CTX-180304). The study was also registered with the National Medical Research Register (NMRR-17-1994-34963) and the US National Library of Medicine—Clinical Trials (NCT03807037).

## Results

The recruitment of patients for this study commenced on 21st January 2019. The current results were based on patients recruited as of 31st January 2021 and the research is ongoing until the target sample size is reached. However, the tabulated data are based on details extracted from both the IJN track care and the patients’ medical records (PMR) as of 31st January 2021.

The total number of patients recruited as of 31st January 2021 was 202 patients (80.8% recruitment rate). Of these, 151 (60.4%) have completed the study, that is, they have at least been followed up to their first post-operative clinic visit. There are currently 28 patients who have yet to complete their enrolment in this study. They include patients who have been discharged but are awaiting their first post-operative clinic appointment. Five patients were lost during follow-up and 10 patients withdrew from the study. There were 8 deaths, equivalent to 3.96% of the study population which is slightly lower as compared to our previous retrospective study [29].

The statistical analyses conducted on the results of our study below were limited to the non-unblinded (i.e. blinded) data set since the study is still ongoing. Hence, the analyses comparing the study group (group that received Tocovid) versus the control group (placebo) will only be done at the end of the study when the groups are unblinded. It is also noteworthy that the current analyses were done on patients with complete information as traced from the track care and the medical records and comparing those that developed POAF to those that didn’t. Consequently, we expect some variations with regard to the total number of patients analysed in each section.

### Patients’ characteristics

Table 1 below on the characteristics of our sample population, shows that the study sample followed a normal distribution with a minimum and maximum age of 39 and 85 years respectively. As expected, the majority of the patients were Malays (69.8%) followed by Chinese (3.7%) who are in fact under-represented if we were to consider the Malaysian cohort. 8.5% of the study populations were Indians who are actually over-represented in our study sample.

**Table 1:** Characteristics of the sample population in our study and their association with POAF

Demographic	Total	Non-POAF group, n (%)	POAF group, n (%)	*p *value
Age (years)	61.44 ± 7.30	61.30 ± 7.62	61.87 ± 6.24	0.68^a^
Gender				0.03*
Male	130	94 (72.31)	36 (27.69)	
Female	26	24 (92.31)	2 (7.69)	
Population				0.94
Malay	132	100 (75.8)	32 (24.2)	
Chinese	7	5 (71.4)	2 (28.6)	
Indian	16	12 (75)	4 (25)	
Other	1	1 (100)	0 (0)	

^a^Test using Independent *t* test

^*^p value significant at 0.05 using the Chi-Square test

There was no statistically significant difference (*p* = 0.68) between the mean age group of POAF patients (61.87) compared to those without (61.30) though patients with POAF tended to be slightly older. This observation is at odds with existing literature [30–33] where advanced age is considered the most consistent risk factor for POAF, a condition attributed to a loss in myocardial fibres coupled with an increase in fibrosis and collagen deposition near the SA node in the atrium which alters the atrial electrical conductivity [34, 35]. One retrospective study [33] with almost 15,000 patients over 2 decades revealed that the chance of developing POAF increases at a higher rate after the age of 55; those who are 72 or older are five times more likely to develop POAF than those who are 55 years old.

However, the current data analysis showed a statistically significant difference (*p* = 0.03) between males and females in developing POAF. Males (27.69%) tended to have a higher preponderance for this phenomenon compared to females (7.69%). This is in line with a study by Filardo et al. [36] who showed over a 9-year study period that women had a statistically significant lower risk of developing POAF with an absolute difference of − 5.3% (95% confidence interval CI, − 10.5% to − 0.6%).

Our preliminary result also revealed no statistically significant difference between the various ethnic groups with respect to POAF. This differs from our earlier study [2] that saw the Indian population, as compared to the other races, having a significantly lower odds of developing POAF. It also contrasted with a Singaporean study [37] which found that, compared to Indians, Malays and Chinese were more prone to develop this condition post-CABG.

### Post-operative AF (POAF) characteristics

Table 2 below refers to the characteristics of POAF. Thirty-eight of our study patients corresponding to 24.36% of the sample developed AF. This figure is slightly lower than in our previous study [2] where 28.7% of patients developed POAF. However, this is an unblinded result and at this point in time, we do not know for certain if Tocovid managed to lower down the POAF rate significantly. Indeed, this incidence rate is within the cited range in the literature [38] that puts the POAF incidence range between 20 and 40%.

**Table 2:** Characteristics of POAF among 156 subjects

Characteristics of AF	n* (%), mean ± SD
Occurrence of POAF	38 (24.36)
Time from surgery to POAF (min)	55.38 ± 29.9
Duration (h)	
≤ 48	13 (41.94)
> 48	18 (58.06)
Number of episodes	
Single	17 (53.13)
Multiple	15 (46.87)
Atrial fibrillation on discharge	0 (0)

Total n varies slightly for each item due to a small amount of missing data in each

The median time for the development of POAF was 54 h after surgery, which was slightly delayed than the oft-cited 48 h post-surgery. The mean time was 55.38 ± 29.9 h post-CABG or on the third day after CABG. We also noticed that in our preliminary results, slightly more than half of the cases (58.06%) developed AF after more than 48 h post-surgery. However, this was within the range cited by many in the literature about POAF mainly occurring within the first week post-surgery at a median time of 2 days post-CABG [38]. Moreover, slightly more than half of our study patients (53.13%) had a single episode of POAF while the remaining 46.87% had multiple episodes. Nonetheless, all of them were discharged in sinus rhythm.

### Pre-operative characteristics

According to the WHO [39], Malaysia has the highest rate of obesity and being overweight among Asian countries: about 65% of the female and 64% of the male population are either obese or overweight. Referring to our Table 3 below, it is therefore not surprising that 53.2% of the study sample was categorised as overweight and 27.56% as obese according to the Asian guidelines [40]. However, obesity is not a predictive factor for POAF and we found no statistically significant difference between the groups. This does not conform to some of the data in the literature [41–44] suggesting that obese patients have significantly higher odds of developing POAF compared to non-obese ones. This suggestion of obesity as a significant independent predictor for postoperative AF is also supported by an earlier work of Sun et al. [45]

**Table 3:** Association between POAF and pre-operative characteristics that were recorded

Pre-operative characteristic	Total*, n (%)	Non-POAF group, n (%)	POAF group, n (%)	*p* value
Body mass index (kg/m^2^)	27.23 ± 4.61	27.43 ± 4.55	27.03 ± 5.20	0.50^a^
< 18.5	2 (1.29)	1 (50)	1 (50)	
18.5–22.9	28 (17.95)	19 (67.90)	9 (32.10)	
23–29.9	83 (53.20)	66 (79.50)	17 (20.50)	
≥ 30	43 (27.56)	32(74.40)	11 (25.60)	
New York heart functional class				0.57^a^
NYHA I	58 (53.71)	43 (74.14)	15 (25.86)	
NYHA II	48 (44.44)	32 (66.70)	16 (33.30)	
NYHA III	2 (1.85)	1 (50)	1 (50)	
NYHA IV	0 (0)	(0)	(0)	
Left ventricular ejection fraction	46.35 ± 15.82	46.96 ± 15.26	44.87 ± 17.25	0.49^b^
Left atrial size (mm)	18.43 ± 5.48	18.11 ± 5.89	19.16 ± 4.43	0.10^c^
Right atrial size (mm)	13.83 ± 4.02	13.58 ± 3.99	14.36 ± 4.09	0.14^c^

Total n varies slightly for each item due to a small amount of missing data in each

^a^Test using Chi Square test

^b^Test using independent *t* test

^c^Test using Mann–Whitney test

Since our study has omitted poor EF < 30% based on our Exclusion Criteria, most of the patients in the study cohort had relatively normal functional status with 53.71% of them in New York Heart Association (NYHA) Functional Class I and 44.44% in Class II. We found the mean left ventricular ejection fraction to be approximately 46%; we also noted that the POAF group has a poorer EF (about 45%) compared to the non-POAF group (about 47%). However, this was not statistically significant. Similarly, we observed no statistically significant difference between the POAF group that had a larger left atrial size compared to the non-POAF group. These two findings were at odds with some of the literature [46–49] that established a correlation between poor EF and left atrial size dilatation to the development of POAF.

### Medical history

We analysed the pre-morbid history of our patients as shown in Table 4 below. We found that the majority of them had the three most common pre-morbid conditions: hypertension (82.76%), diabetes mellitus (70.09%), and hypercholesterolaemia (85.09%). However, no statistically significant difference between the groups was detected. Similarly, we analysed the history of chronic kidney disease which has been associated with POAF in some of the literature [50, 51]. Again, we found no significant difference between the two groups.

**Table 4:** Association between POAF and underlying medical conditions on admission

Medical condition	Total*, n (%)	Non-POAF group, n (%)	POAF group, n (%)	χ^2^	*p *value
COPD					
Yes	2 (1.7)	2 (100)	0 (0)	–	1.00^a^
No	114 (98.3)	79 (69.3)	35 (30.7)		
Asthma					
Yes	1 (0.9)	1 (100)	0 (0)	–	1.00^a^
No	115 (99.1)	80 (69.6)	35 (30.4)		
Hypertension					
Yes	96 (82.8)	67 (69.8)	29 (30.2)	0.000	0.97
No	20 (17.2)	14 (70.0)	6 (30.0)		
Diabetes mellitus					
Yes	82 (70.1)	59 (72)	23 (28)	0.952	0.33
No	35 (29.9)	22 (62.9)	13 (37.1)		
Hypercholesterolemia					
Yes	97 (85.1)	69 (71.1)	28 (28.90)	1.030	0.31
No	17 (14.9)	10 (58.8)	7 (41.2)		
Chronic kidney disease					
Yes	15 (13.0)	10 (66.7)	5 (33.33)	–	0.77^a^
No	100 (87.0)	70 (70.0)	30 (30.0)		
Current or ex-smoker					
Yes	63 (58.9)	41 (65.1)	22 (34.9)	6.074	0.014*
No	44 (41.1)	38 (86.4)	6 (13.6)		
Alcohol intake					
Yes	6 (6.1)	3 (50)	3 (50)	–	0.173^a^
No	93 (93.9)	70 (75.3)	23 (24.)		

Total n varies slightly for each item due to a small amount of missing data in each

*COPD* chronic obstructive pulmonary disease

^a^Test using Fisher Exact Test

^*^p value significant at *p* < 0.05 using Chi-Square test

In our analysis of smoking habits, we observed that the current or ex-smoker group had a lower incidence of POAF compared to the non-smoker group, with a statistically significant difference (*p* = 0.0014). This is mirrored by another study [52] that showed a statistically significant difference between the groups where smokers tended to have a lower incidence of POAF (*p* < 0.05). That study [52] also uncovered that the postoperative complications incidence did not differ significantly among smoking status groups. Nevertheless, the researchers strongly recommended cessation of smoking for at least 4 weeks before surgery in order to improve post-operative outcomes and reduce the risk of post-operative pulmonary complications.

### Operative details

Since we have excluded off-pump surgery in the Exclusion Criteria, and taking into account that IJN is an on-pump centre, we mainly performed isolated CABG (91.55%) while combined valve surgery was only 8.45% as depicted in Table 5 below. Only the mitral valve was involved in the combined valve surgery, with two-third being mitral valve repair and the other one-third consisting of mitral valve replacement. However, there was no statistically significant difference between them in terms of developing POAF.

**Table 5:** Association between post-operative atrial fibrillation (POAF) and patient operative details

Operative details	Total*, n (%)	Non-POAF group, n (%)	POAF group, n (%)	*p* value
Surgery type				
CABG alone	130 (91.55)	96 (73.80)	34 (26.20)	0.59
CABG + valve	12 (8.45)	8 (66.67)	4 (33.33)	
Bypass time (in min)	95 ± 46	96 ± 57	94 ± 38	0.87
Cross-clamp time (in min)	71 ± 34	70 ± 36	73 ± 31	0.81

Total n varies slightly for each item due to a small amount of missing data in each

*CABG* coronary bypass grafting

We observed a mean cross-clamp time of 71 ± 34 min ranging from 22 to 244 min, and the mean bypass time of 95 ± 46 min ranging from 49 to 304 min. Similarly, there was no statistically significant difference between the two groups though it is an established fact that both cross-clamp and bypass time were associated with the development of POAF [53, 54].

### Post-operative outcomes

The discussion on the post-operative outcome is perhaps the most intriguing where POAF has been associated with numerous adverse outcomes including a two to four-fold increase in stroke, reoperation, infection, renal failure, respiratory complications and cerebral insults in addition to a two-fold increase in all-cause 30-day mortality [55–57]. While this might not be a direct correlation, it is certainly contributory and plays a part in the increase in morbidity and mortality after cardiac surgery [53]. Based on Table 6 below, the most significant association was the three-fold increase in deaths among patients with POAF (*p* = 0.008). The mortality rate in our study population was 3.96% which was slightly lower than the mortality rate (around 4.66%) in our earlier publication [29]. However, our finding of the increase in mortality among POAF patents not only confirmed the previous literature findings but also the most recent paper by Emma Thorén et al. [58] that associated POAF with mortality even after adjustment for AF during follow-up.

**Table 6:** Association between POAF and post-operative outcomes in 156 subjects

Post-operative outcomes	Total*, n (%)	Non-POAF group, n (%)	POAF group, n (%)	*p* value
Stroke				
Yes	4 (4.0)	2 (50)	2 (50)	0.58
No	97 (96.0)	69 (71.1)	28 (28.9)	
Sternal infection				
Yes	3 (3.0)	3 (100)	0 (0)	0.55
No	98 (97.0)	68 (69.4)	30 (30.6)	
Respiratory problems				
Yes	6 (5.9)	3 (50)	3 (50)	0.36
No	95 (94.1)	68 (71.6)	27 (28.4)	
Renal failure requiring dialysis				
Yes	5 (5.0)	4 (80)	1 (20)	1.00
No	96 (95.0)	67 (69.8)	29 (30.2)	
Endocrine problems				
Yes	1 (1.0)	1 (100.0)	0 (0)	1.00
No	100 (99.0)	70 (70.0)	30 (30.0)	
Pleural effusion				
Yes	5 (5.0)	3 (60)	2 (40)	0.63
No	96 (95.0)	68 (70.8)	28 (29.2)	
Cardiac tamponade				
Yes	14 (13.9)	13 (92.9)	1 (7.1)	0.06
No	87 (86.1)	58 (66.7)	29 (33.3)	
Fever				
Yes	6 (5.9)	4 (66.7)	2 (33.3)	1.00
No	95 (94.1)	67 (70.5)	28 (29.5)	
Hyperkalaemia				
Yes	4 (4.0)	3 (75)	1 (25)	1.00
No	97 (96.0)	68 (70.1)	29 (29.9)	
Others				
Yes	3 (3.0)	1 (33.33)	2 (66.7)	0.21
No	98 (97.0)	70 (71.4)	28 (28.6)	
Death				
Yes	8 (7.9)	2 (25)	6 (75)	0.008*
No	93 (92.1)	69 (74.2)	24 (25.8)	

Others: Low blood pressure, multiple premature ventricular complexes

Total n varies slightly for each item due to a small amount of missing data in each

^*^p value significant at < 0.05 using the Fisher Exact test

We also reviewed all the other common complications such as stroke, sternal wound infection, respiratory problems, renal failure requiring dialysis, endocrine problems, pleural effusion, cardiac tamponade, fever and hyperkalaemia—none were significantly correlated.

### Postoperative stay

Studies elsewhere have shown that patients with POAF have a prolonged ICU stay, with an additional 2–5 days in the hospital [59, 60].In the US, patients who develop POAF would be utilising an average of USD10,000–USD20,000 in additional hospital treatment costs [61]. Furthermore, the healthcare expenditures related to the management of POAF in the US were estimated at over USD 1 billion per year [34]. Another study [62] conducted at the Instituto de Cardiologia, Bogota DC, Colombia, also demonstrated that the occurrence of POAF was associated with a significant increase in the utilisation of hospital resources and in direct cost in patient management. Unfortunately, to date, no study has been conducted in Malaysia on the financial burden in managing POAF patients. Nevertheless, we believe that the results would not be much different.

The results tabulated in Table 7 above reflect what we actually expected. We noted a statistically significant difference in the mean duration of ICU stay (*p* = 0.01), the total duration of hospital stay (*p* = 0.04) and reintubation (*p* = 0.045). Our findings were similar to one study [63] that collected and evaluated data from 28 centres across the United States, Italy and Argentina via multivariate adjusted models. The researchers found that the occurrence of POAF was significantly correlated with an increase in resource utilisation, including length of stay in ICU and total hospital stay. In another study that compared on-pump versus off-pump surgery [64], a statistically significant higher rate of reintubation and adverse outcome with POAF was observed.

**Table 7:** Association between POAF and duration of stay as well as duration of ventilation

Duration	Total, median ± IQR / n (%)	Non-POAF group, median ± IQR / n (%)	POAF group, median ± IQR /n (%)	*p*-value
Duration in ICU (min)	2767.50 ± 3927	1742 ± 2354	3847.50 ± 4773	0.01*
Duration in HDU (min)	1522.50 ± 1584	1480 ± 1514	1755 ± 2743	0.33
Duration of ventilation (min)	1187.50 ± 442	1157 ± 391	1230 ± 875	0.22
Duration of hosp. stay (day)	8.0 ± 3	7.0 ± 3	9.0 ± 3	0.04*
Reintubation				0.045**
Yes	4 (2.6)	1 (25)	3 (75)	
No	152 (97.4)	117 (77.0)	35 (23.0)	

Total n varies slightly for each item due to a small amount of missing data in each

*ICU* intensive care unit, *HDU* high dependency unit

^*^p value significant at < 0.05 using Mann–Whitney Test

^**^p value significant at < 0.05 using Fisher Exact test

## Discussion

Without doubt, POAF is the most common complication following CABG or CABG/valve surgery in 24.36% of our study population. This is lower than in our previous study [2] which reported an incidence rate of 28.7%. Nonetheless, the current study is still ongoing and the results will definitely change. What we anticipate even more eagerly is whether our prophylactic intervention using Tocovid, a tocotrienol-rich compound, would reduce the incidence of POAF in the study arm.

It is also noteworthy that the magnitude of increase in mortality rate and the length of stay in the intensive care unit and the total hospital stay, apart from the increased rate of reintubation, have remained almost unchanged over the years [65, 66]. This is despite advances in post-operative care of cardiac surgery patients. Consequently, in trying to alleviate this situation by providing a possible solution via prophylactic means, our project is intriguing. While we are unsure if our intervention would work, the scientific theory propelling our endeavour cannot be doubted. As Danish physicist Niels Bohr aptly puts it, “It’s very difficult to make predictions, especially about the future.”

The pathogenesis of POAF is now thought to be due to oxidative stress and inflammation. Cardiac surgery itself inflicts a trauma on the heart, and the use of cardiopulmonary bypass produces ischaemic injury. Reperfusion injury following when an artery is grafted leads to oxidative stress and the production of pro-inflammatory molecules, resulting in leucocyte activation and the production of nitrous oxide and reactive oxygen species [55, 67]. It has also been demonstrated in human studies that a correlation exists between systemic inflammation and oxidative stress and the development of POAF [68, 69]. Guided by this information, we ventured into this research project. If our hypothesis is proven correct, we would be able to reduce the morbidity and mortality associated with POAF together with the total time spent in ICU and the overall hospital stay—this alone would reduce the cost in patient management and lessen the strain on the healthcare system.

Research has shown that POAF has been correlated with longer and costlier lengths of stay both in the ICU and total hospital stay, besides the rate of readmission. [65, 66, 70]. In the USA, these outcomes translate into a substantial financial impact amounting to approximately USD 2 billion per year [34] out of a total expenditure of more than USD 6 billion related to AF care in that country [34, 61, 71, 72]. Despite the absence of any data either at the IJN of Kuala Lumpur or elsewhere countrywide regarding the total cost incurred in managing patients with this type of complication, it is predicted that the total costs would be massive. Hence, it is undeniable that improving the health condition of patients would have a positive effect on their economic activity, and subsequently, the national economy itself.

In devising ways to address this issue, we are aware that several non-drugs or non-pharmacological compounds have been used in the research to prevent POAF—namely polyunsaturated fatty acids (PUFAs), vitamin C, or a combination of vitamins C and E [73]. While focusing on the non-pharmacological compounds, we noticed that as a known dietary antioxidant, PUFAs have been shown to confer potential benefits in reducing cardiovascular morbidity in animal models despite limited evidence for their use as prophylaxis for POAF [74]. However, a very recent paper by Rubanenko and Rubanenko [75] showed that patients treated with PUFAs had less activation of inflammation and oxidative stress after CABG, with a significant decrease in the prevalence of POAF after CABG. A 2017 meta-analysis [76] of 19 randomised controlled trials (RCTs) found a reduction in POAF. Similarly, a 2018 meta-analysis [77] that included 14 RCTs also showed a significant reduction of POAF with PUFAs as compared to controls, although this effect was found only in CABG, not valve surgery. It is very promising that an antioxidant had been shown to have an effect in preventing POAF, especially when our hypothesis is built upon a similar promise that tocotrienol-rich Tocovid, itself a powerful anti-oxidant, could ameliorate and prevent the occurrence of POAF.

Another compound which has been studied quite extensively is vitamin C, a compound known to reduce oxidative stress. In a 2016 meta-analysis [78] of 7 RCTs, the incidence of POAF was found to be reduced as compared to controls. However, a more recent RCT [79] involving 314 on-pump patients found to have no difference in POAF, ICU stay and the total hospital stay, and to date, there are still no guidelines referring its use for POAF prophylaxis. However, when we look at a combined antioxidants use where vitamin C is combined with vitamin E and PUFAs, a 2013 study [80] showed that there was a significant reduction in the incidence of POAF among patients receiving antioxidants as compared to controls. It was, therefore, not surprising then when the authors recommended the use of a combination of these antioxidants as an effective, safe and cheap prophylaxis against the onset of POAF. However, until today, no guidelines reference of such a protocol is available.

Based on all the above-mentioned studies, it is highly likely that a powerful antioxidant and anti-inflammatory agent such as Tocovid might be able to mitigate the occurrence of POAF; this is where this study might be able to pave the way in finding such a solution. While it is still too early to make any conclusion, the scientific basis to pursue such a study is well established and it will take a few more months before this study is concluded and the study groups unblinded.

## Limitations

The main limitation in the study is with regard to the current COVID-19 pandemic that made it difficult to recruit patients for enrolment in the study. There was a reduction in the numbers of patients enrolled due to the limited number of ICU beds.

## Conclusion

At present, we can conclude that the incidence of the occurrence of POAF remains high (slightly above 20%), exacting a high toll in terms of worse patient outcomes. It increases both the mortality and hospital care costs since we observed a statistically significant increase in ICU stay and total hospital stay. Consequently, a non-invasive, highly effective, low-risk and cheaper alternative in preventive therapy to reduce the incidence of POAF would be a huge step forward in managing this common problem. It still remains uncertain whether this prophylactic intervention for reducing POAF should be limited to high-risk patients or if it should be extended to all patients. A knowledge gap persists in this area, as is the mechanism of the development of POAF; it is highly unlikely that there is a single unifying mechanism for the development of this arrhythmia although the inflammatory and oxidative pathways are most likely involved in exacting the common outcome of this major complication of CABG.

## Supplementary Information

**Additional file 1**. Set 1: Raw Data.

**Additional file 2**. Set 2: Output Data.

## Data Availability

Harvard Dataverse: Replication Data for: Tocovid, a tocotrienol-rich vitamin E in preventing atrial fibrillation in post-coronary artery bypass grafting (CABG) surgery: A preliminary result. Https: https://doi.org/10.7910/DVN/HX0AVU. This project contains the following underlying data: Set 1: Raw Data (Additional file 1). Set 2: Output Data (Additional file 2).
