# Supplementary material for: A double-blind randomised controlled trial on the effect of Tocovid, a tocotrienol-rich capsule on postoperative atrial fibrillation at the National Heart Institute, Kuala Lumpur: an interim blinded analysis
Source: J Cardiothorac Surg. 2021 Nov 24;16:340. doi: 10.1186/s13019-021-01721-6 (PMC8611632; doi:10.1186/s13019-021-01721-6)
Supplement: Supplementary file 2 — Additional file 2. Set 2: Output Data. [file 13019_2021_1721_MOESM2_ESM.pdf]

```
EXAMINE VARIABLES=AGE BY AF
  /PLOT BOXPLOT STEMLEAF NPPLOT
  /COMPARE GROUPS
  /STATISTICS DESCRIPTIVES
  /CINTERVAL 95
  /MISSING LISTWISE
  /NOTOTAL.
```

Explore

Notes

Output Created		01-MAR-2021 14:30:58
Comments		
Input	Data	C:\Users\user\Downloads\ImAnisah Statistic & SPSS\TOCO T3\Data toco.sav
	Active Dataset	DataSet1
	Filter	<none>
	Weight	<none>
	Split File	<none>
	N of Rows in Working Data File	156
Missing Value Handling	Definition of Missing	User-defined missing values for dependent variables are treated as missing.
	Cases Used	Statistics are based on cases with no missing values for any dependent variable or factor used.
Syntax		EXAMINE VARIABLES=AGE BY AF<br>/PLOT BOXPLOT STEMLEAF NPPLOT<br>/COMPARE GROUPS<br>/STATISTICS DESCRIPTIVES<br>/CINTERVAL 95<br>/MISSING LISTWISE<br>/NOTOTAL.
Resources	Processor Time	00:00:02.95
	Elapsed Time	00:00:07.96

AF

Case Processing Summary

	AF	Cases					
		Valid		Missing		Total	
		N	Percent	N	Percent	N	Percent
AGE	No	118	100.0%	0	0.0%	118	100.0%
	Yes	38	100.0%	0	0.0%	38	100.0%

Descriptives

	AF			Statistic	Std. Error
AGE	No	Mean		61.30	.702
		95% Confidence Interval for Mean	Lower Bound	59.91	
			Upper Bound	62.69	
		5% Trimmed Mean		61.32	
		Median		61.00	
		Variance		58.125	
		Std. Deviation		7.624	
		Minimum		39	
		Maximum		85	
		Range		46	
		Interquartile Range		11	
		Skewness		-.029	.223
		Kurtosis		.224	.442
	Yes	Mean		61.87	1.012
		95% Confidence Interval for Mean	Lower Bound	59.82	
			Upper Bound	63.92	
		5% Trimmed Mean		62.07	
		Median		61.50	
		Variance		38.928	
		Std. Deviation		6.239	
		Minimum		49	
		Maximum		72	
		Range		23	
		Interquartile Range		9	
		Skewness		-.488	.383
		Kurtosis		-.634	.750

Tests of Normality

	AF	Kolmogorov-Smirnov[a]			Shapiro-Wilk		
		Statistic	df	Sig.	Statistic	df	Sig.
AGE	No	.062	118	.200*	.993	118	.832
	Yes	.140	38	.060	.950	38	.086

*. This is a lower bound of the true significance.

a. Lilliefors Significance Correction

AGE

Stem-and-Leaf Plots

```
AGE Stem-and-Leaf Plot for
AF= No

 Frequency    Stem &  Leaf

     1.00 Extremes    (=<39)
      .00        4 .
     2.00        4 .  45
      .00        4 .
     5.00        4 .  99999
     3.00        5 .  011
     6.00        5 .  222233
    10.00        5 .  4444455555
     9.00        5 .  666777777
    13.00        5 .  8888888999999
    13.00        6 .  0000000111111
    12.00        6 .  222223333333
     5.00        6 .  44555
    12.00        6 .  666666677777
    11.00        6 .  88888999999
     7.00        7 .  0000111
     4.00        7 .  2233
     3.00        7 .  455
     1.00        7 .  6
     1.00 Extremes    (>=85)

 Stem width:        10
 Each leaf:        1 case(s)



AGE Stem-and-Leaf Plot for
AF= Yes

 Frequency    Stem &  Leaf

     2.00        4 .  99
     3.00        5 .  114
     7.00        5 .  5567899
     9.00        6 .  000111124
    14.00        6 .  55566677788899
```

```
    3.00        7 .  002

Stem width:        10
Each leaf:        1 case(s)
```

Normal Q-Q Plots

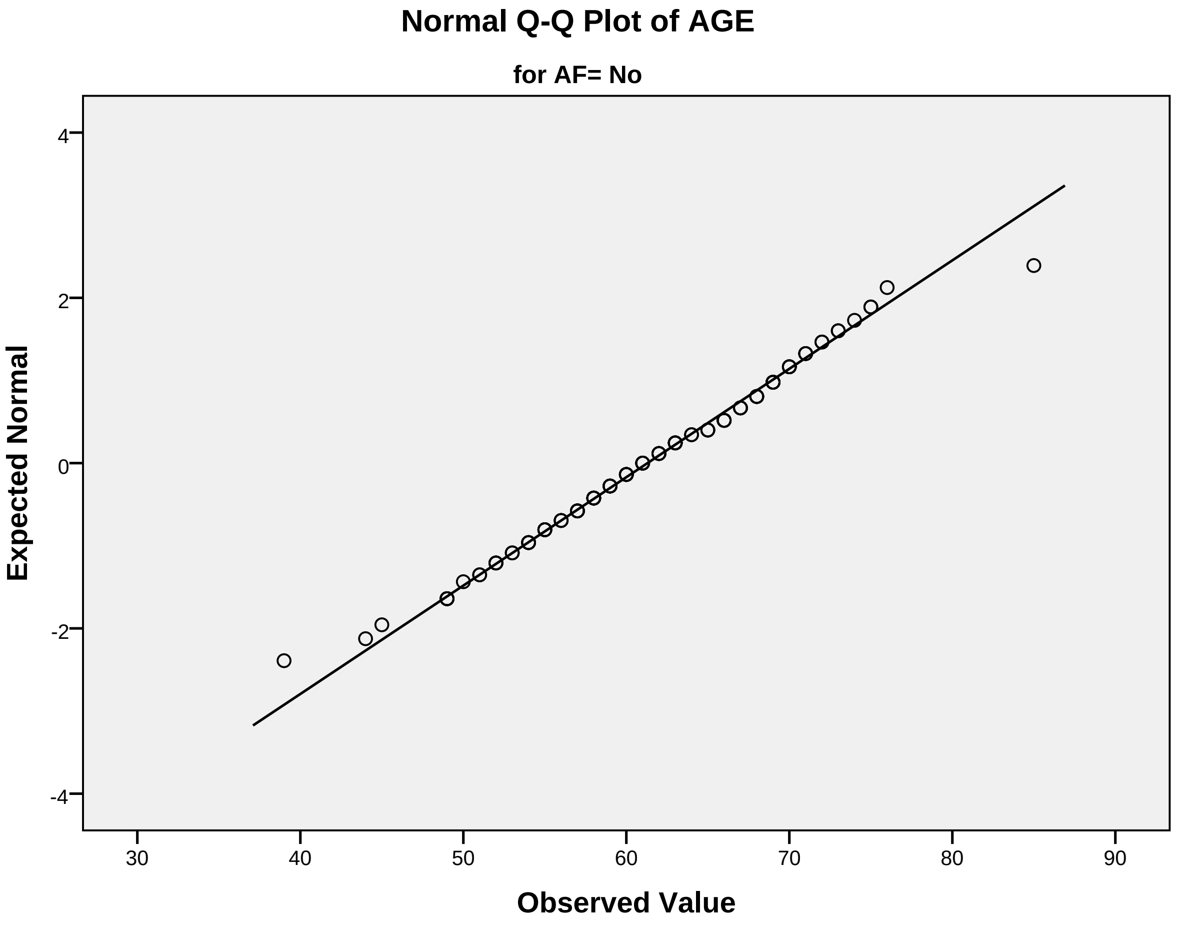

Normal Q-Q Plot of AGE

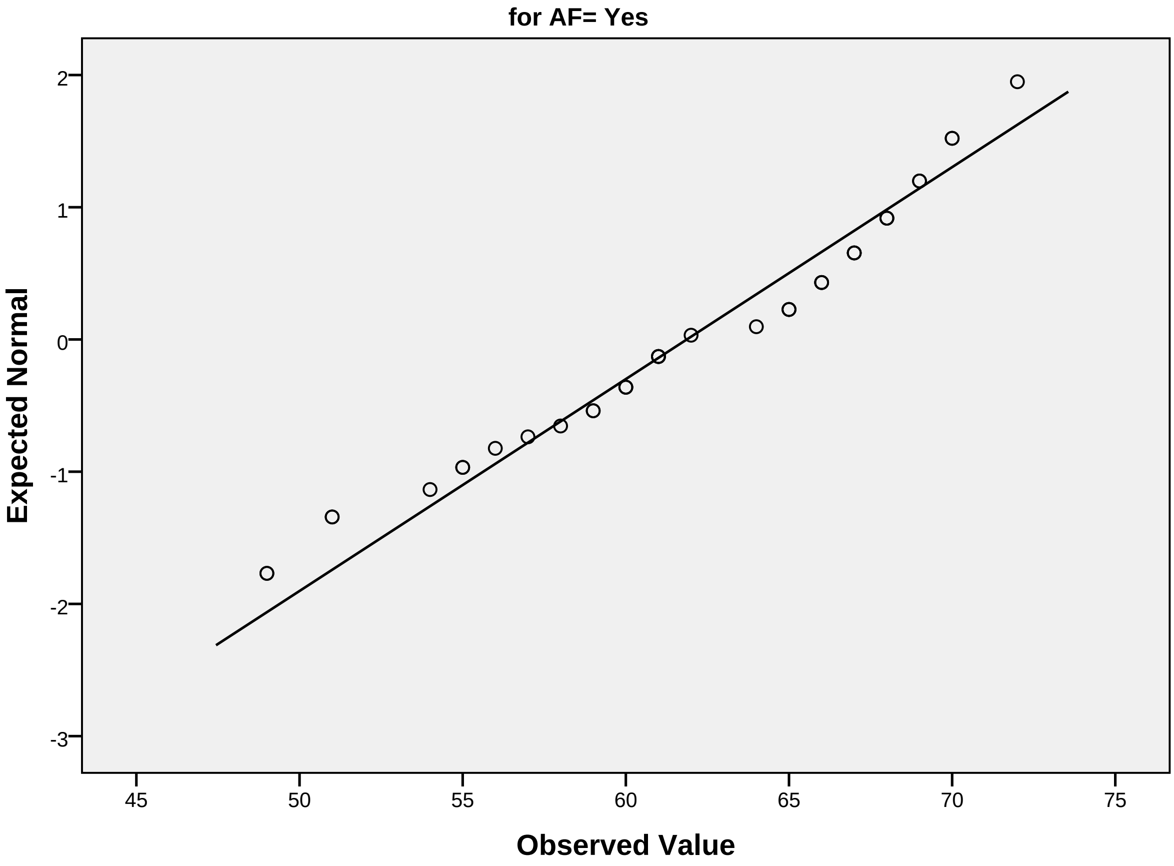

Detrended Normal Q-Q Plots

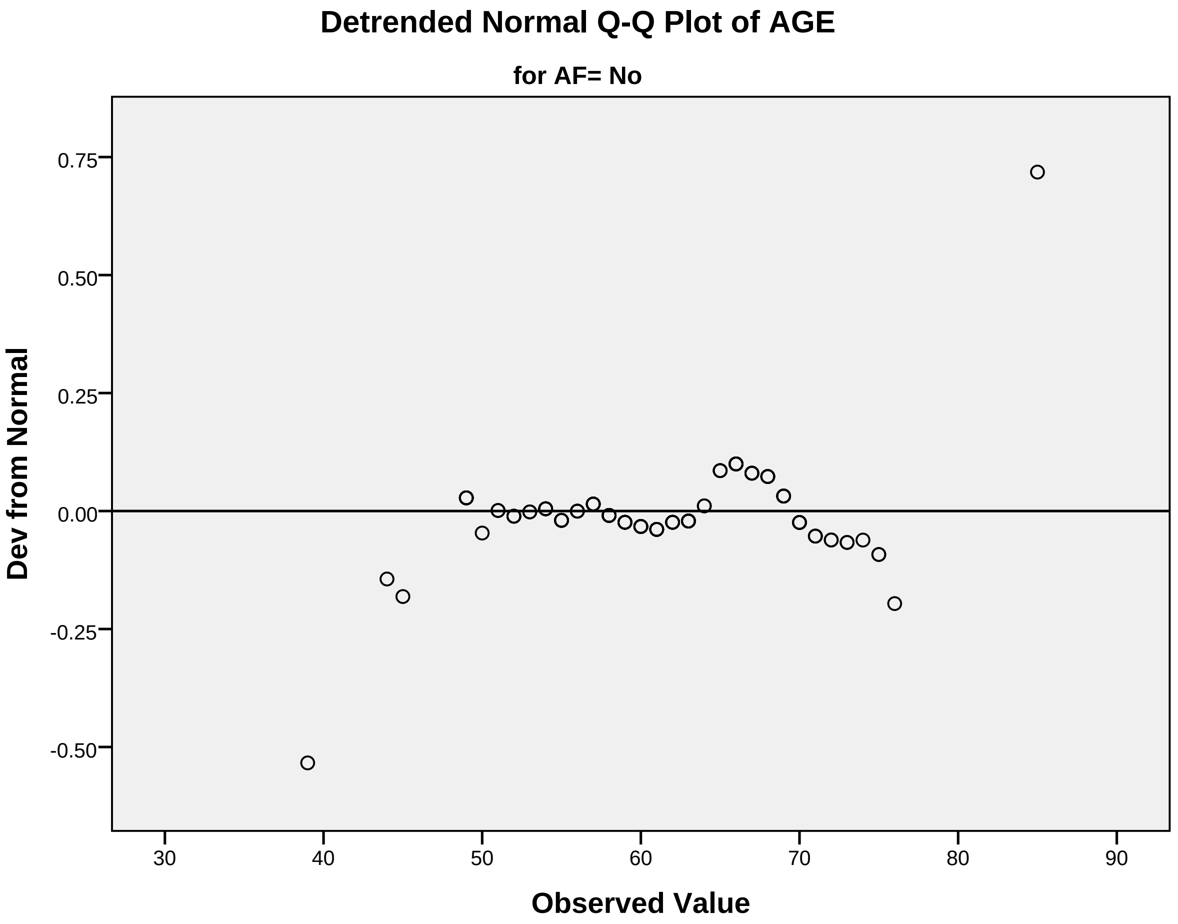

Detrended Normal Q-Q Plot of AGE
for AF= No
Dev from Normal
0.75
0.50
0.25
0.00
-0.25
-0.50
30
40
50
60
70
80
90
Observed Value

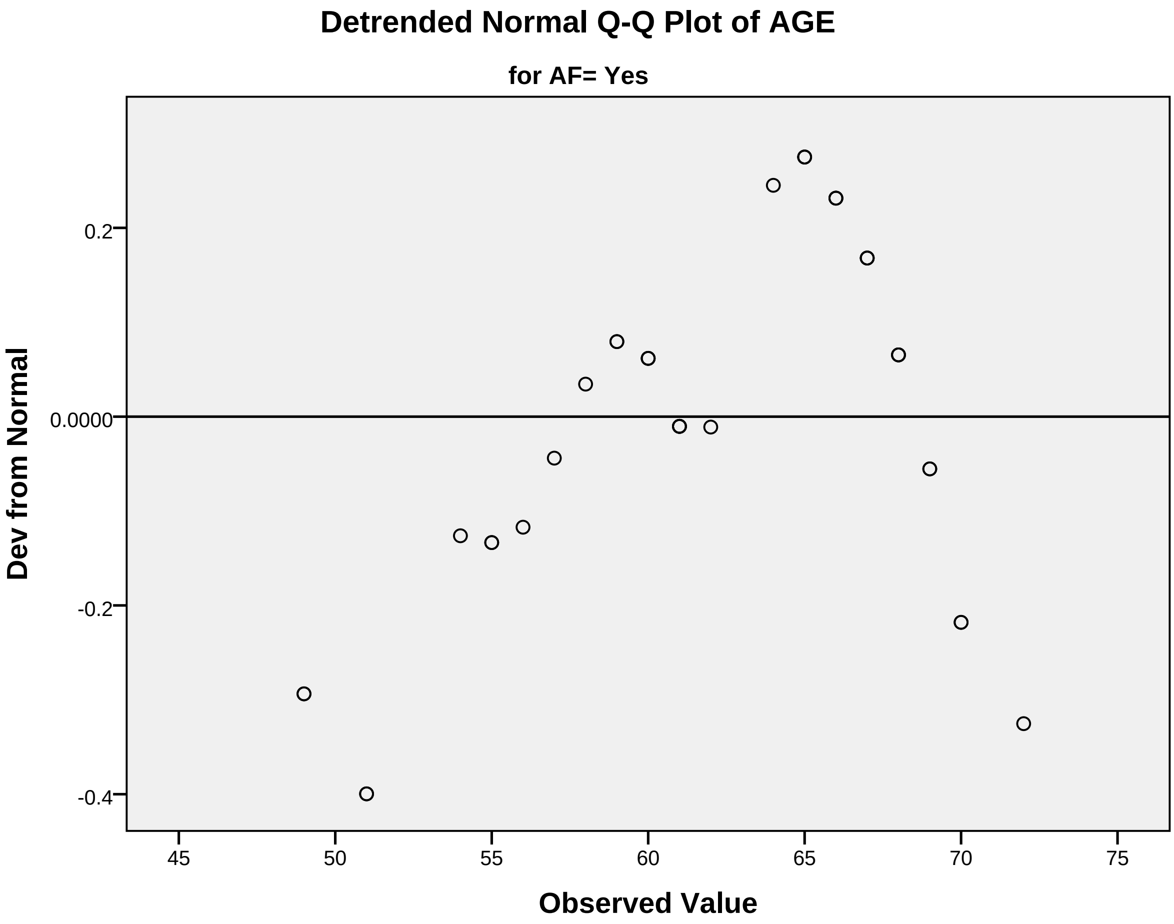
Detrended Normal Q-Q Plot of AGE
for AF= Yes
Dev from Normal
0.2
0.0000
-0.2
-0.4
45
50
55
60
65
70
75
Observed Value

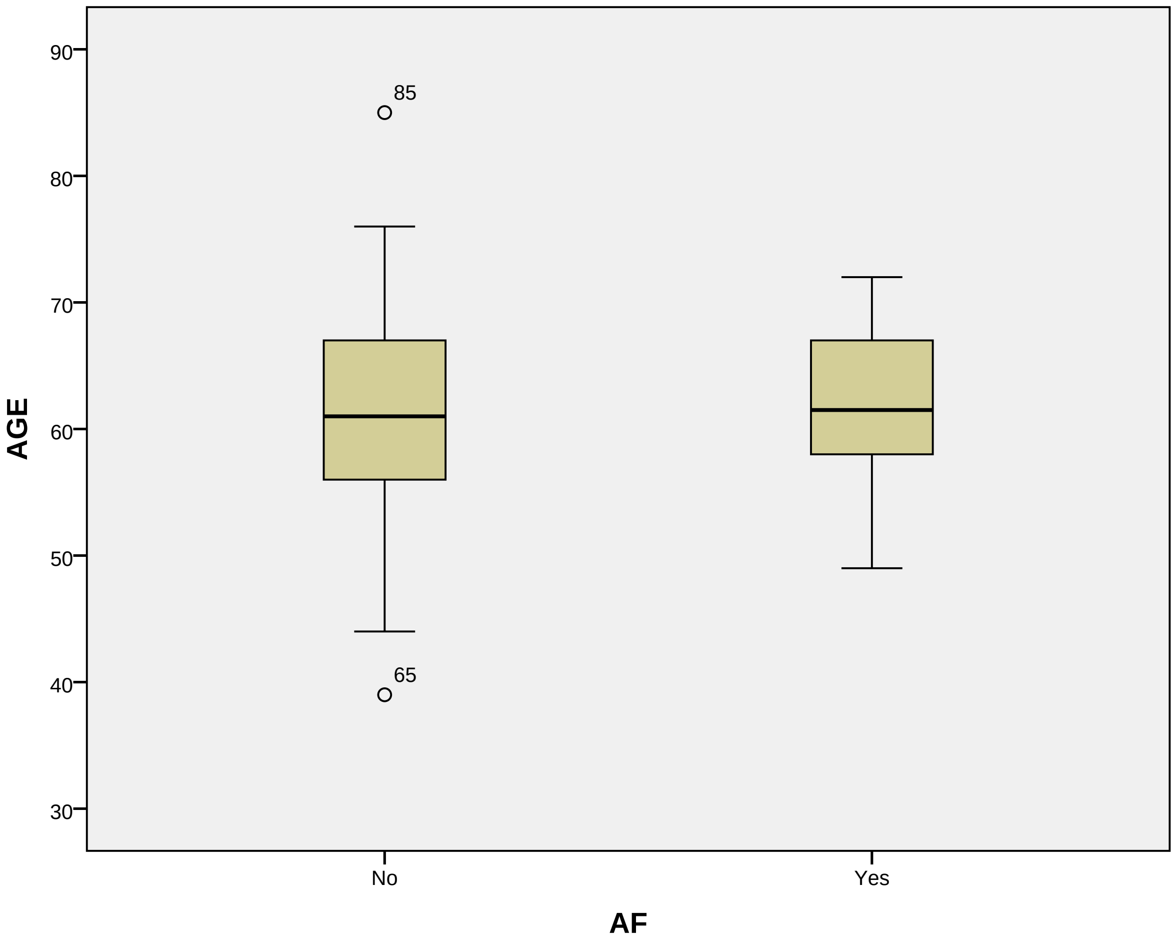


```
T-TEST GROUPS=AF(0 1)
 /MISSING=ANALYSIS
 /VARIABLES=AGE
 /CRITERIA=CI(.95).
```

T-Test

Notes

Output Created		01-MAR-2021 14:31:47
Comments		
Input	Data	C:\Users\user\Downloads\ImAnisah Statistic & SPSS\TOCO T3\Data toco.sav
	Active Dataset	DataSet1
	Filter	<none>
	Weight	<none>
	Split File	<none>
	N of Rows in Working Data File	156
Missing Value Handling	Definition of Missing	User defined missing values are treated as missing.
	Cases Used	Statistics for each analysis are based on the cases with no missing or out-of-range data for any variable in the analysis.
Syntax		T-TEST GROUPS=AF(0 1)<br>/MISSING=ANALYSIS<br>/VARIABLES=AGE<br>/CRITERIA=CI(.95).
Resources	Processor Time	00:00:00.02
	Elapsed Time	00:00:00.24

Group Statistics

	AF	N	Mean	Std. Deviation	Std. Error Mean
AGE	No	118	61.30	7.624	.702
	Yes	38	61.87	6.239	1.012

Independent Samples Test

		Levene's Test for Equality of Variances		t-test for Equality of Means						
									95% Confidence Interval of the Difference	
		F	Sig.	t	df	Sig. (2-tailed)	Mean Difference	Std. Error Difference	Lower	Upper
AGE	Equal variances assumed	1.342	.248	-.419	154	.676	-.572	1.364	-3.267	2.124
	Equal variances not assumed			-.464	75.609	.644	-.572	1.232	-3.025	1.881

```
CROSSTABS
 /TABLES=GENDER ETHNIC BY AF
 /FORMAT=AVALUE TABLES
 /STATISTICS=CHISQ
 /CELLS=COUNT ROW COLUMN
 /COUNT ROUND CELL.
```

Crosstabs

Notes

Output Created		01-MAR-2021 14:32:17
Comments		
Input	Data	C:\Users\user\Downloads\ImAnisah Statistic & SPSS\TOCO T3\Data toco.sav
	Active Dataset	DataSet1
	Filter	<none>
	Weight	<none>
	Split File	<none>
	N of Rows in Working Data File	156
Missing Value Handling	Definition of Missing	User-defined missing values are treated as missing.
	Cases Used	Statistics for each table are based on all the cases with valid data in the specified range(s) for all variables in each table.
Syntax		CROSSTABS /TABLES=GENDER ETHNIC BY AF /FORMAT=AVALUE TABLES /STATISTICS=CHISQ /CELLS=COUNT ROW COLUMN...
Resources	Processor Time	00:00:00.02
	Elapsed Time	00:00:00.04
	Dimensions Requested	2
	Cells Available	524245

Case Processing Summary

	Cases					
	Valid		Missing		Total	
	N	Percent	N	Percent	N	Percent
GENDER * AF	156	100.0%	0	0.0%	156	100.0%
ETHNIC * AF	156	100.0%	0	0.0%	156	100.0%

GENDER * AF

Crosstab

			AF		
			No	Yes	Total
GENDER	Male	Count	94	36	130
		% within GENDER	72.3%	27.7%	100.0%
		% within AF	79.7%	94.7%	83.3%
	Female	Count	24	2	26
		% within GENDER	92.3%	7.7%	100.0%
		% within AF	20.3%	5.3%	16.7%
Total		Count	118	38	156
		% within GENDER	75.6%	24.4%	100.0%
		% within AF	100.0%	100.0%	100.0%

Chi-Square Tests

	Value	df	Asymptotic Significance (2-sided)	Exact Sig. (2-sided)	Exact Sig. (1-sided)
Pearson Chi-Square	4.704[a]	1	.030		
Continuity Correction[b]	3.681	1	.055		
Likelihood Ratio	5.709	1	.017		
Fisher's Exact Test				.043	.021
Linear-by-Linear Association	4.674	1	.031		
N of Valid Cases	156				

a. 0 cells (0.0%) have expected count less than 5. The minimum expected count is 6.33.

b. Computed only for a 2x2 table

ETHNIC * AF

Crosstab

			AF		Total
			No	Yes	
ETHNIC	Malay	Count	100	32	132
		% within ETHNIC	75.8%	24.2%	100.0%
		% within AF	84.7%	84.2%	84.6%
	Chinese	Count	5	2	7
		% within ETHNIC	71.4%	28.6%	100.0%
		% within AF	4.2%	5.3%	4.5%
	Indian	Count	12	4	16
		% within ETHNIC	75.0%	25.0%	100.0%
		% within AF	10.2%	10.5%	10.3%
	Others	Count	1	0	1
		% within ETHNIC	100.0%	0.0%	100.0%
		% within AF	0.8%	0.0%	0.6%
Total		Count	118	38	156
		% within ETHNIC	75.6%	24.4%	100.0%
		% within AF	100.0%	100.0%	100.0%

Chi-Square Tests

	Value	df	Asymptotic Significance (2-sided)
Pearson Chi-Square	.394[a]	3	.941
Likelihood Ratio	.628	3	.890
Linear-by-Linear Association	.004	1	.948
N of Valid Cases	156		

a. 4 cells (50.0%) have expected count less than 5. The minimum expected count is .24.

```
DESCRIPTIVES VARIABLES=AGE BMI OnsetAFGrp AFnumber
  /STATISTICS=MEAN STDDEV RANGE MIN MAX.
```

Descriptives

Notes

Output Created		01-MAR-2021 14:33:43
Comments		
Input	Data	C:\Users\user\Downloads\ImAnisah Statistic & SPSS\TOCO T3\Data toco.sav
	Active Dataset	DataSet1
	Filter	<none>
	Weight	<none>
	Split File	<none>
	N of Rows in Working Data File	156
Missing Value Handling	Definition of Missing	User defined missing values are treated as missing.
	Cases Used	All non-missing data are used.
Syntax		DESCRIPTIVES VARIABLES=AGE BMI OnsetAFGrp AFnumber /STATISTICS=MEAN STDDEV RANGE MIN MAX.
Resources	Processor Time	00:00:00.02
	Elapsed Time	00:00:00.01

Descriptive Statistics

	N	Range	Minimum	Maximum	Mean	Std. Deviation
AGE	156	46	39	85	61.44	7.296
BMI	156	24.87	17.60	42.47	27.2331	4.60715
OnsetAFGrp	31	1	0	1	.58	.502
AFnumber	32	5	1	6	1.94	1.390
Valid N (listwise)	31					

```
FREQUENCIES VARIABLES=AFnumber OnsetAFGrp
  /NTILES=4
  /STATISTICS=STDDEV MEAN
  /ORDER=ANALYSIS.
```

Frequencies

Notes

Output Created		01-MAR-2021 14:34:43
Comments		
Input	Data	C:\Users\user\Downloads\ImAnisah Statistic & SPSS\TOCO T3\Data toco.sav
	Active Dataset	DataSet1
	Filter	<none>
	Weight	<none>
	Split File	<none>
	N of Rows in Working Data File	156
Missing Value Handling	Definition of Missing	User-defined missing values are treated as missing.
	Cases Used	Statistics are based on all cases with valid data.
Syntax		FREQUENCIES VARIABLES=AFnumber OnsetAFGrp<br>/NTILES=4<br>/STATISTICS=STDDEV MEAN<br>/ORDER=ANALYSIS.
Resources	Processor Time	00:00:00.02
	Elapsed Time	00:00:00.02

Statistics

		AFnumber	OnsetAFGrp
N	Valid	32	31
	Missing	124	125
Mean		1.94	.58
Std. Deviation		1.390	.502
Percentiles	25	1.00	.00
	50	1.00	1.00
	75	2.00	1.00

Frequency Table

AFnumber

		Frequency	Percent	Valid Percent	Cumulative Percent
Valid	1	17	10.9	53.1	53.1
	2	9	5.8	28.1	81.3
	3	1	.6	3.1	84.4
	4	2	1.3	6.3	90.6
	5	2	1.3	6.3	96.9
	6	1	.6	3.1	100.0
	Total	32	20.5	100.0	
Missing	System	124	79.5		
Total		156	100.0		

OnsetAFGrp

		Frequency	Percent	Valid Percent	Cumulative Percent
Valid	<48	13	8.3	41.9	41.9
	>48	18	11.5	58.1	100.0
	Total	31	19.9	100.0	
Missing	System	125	80.1		
Total		156	100.0		

```
CROSSTABS
 /TABLES=BMIGroup NYHA BY AF
 /FORMAT=AVALUE TABLES
 /STATISTICS=CHISQ
 /CELLS=COUNT ROW COLUMN
 /COUNT ROUND CELL.
```

Crosstabs

Notes

Output Created		01-MAR-2021 14:35:42
Comments		
Input	Data	C:\Users\user\Downloads\ImAnisah Statistic & SPSS\TOCO T3\Data toco.sav
	Active Dataset	DataSet1
	Filter	<none>
	Weight	<none>
	Split File	<none>
	N of Rows in Working Data File	156
Missing Value Handling	Definition of Missing	User-defined missing values are treated as missing.
	Cases Used	Statistics for each table are based on all the cases with valid data in the specified range(s) for all variables in each table.
Syntax		CROSSTABS<br>/TABLES=BMIGroup NYHA BY AF<br>/FORMAT=AVALUE TABLES<br>/STATISTICS=CHISQ<br>/CELLS=COUNT ROW COLUMN<br>/COUNT ROUND CELL.
Resources	Processor Time	00:00:00.03
	Elapsed Time	00:00:00.03
	Dimensions Requested	2
	Cells Available	524245

Case Processing Summary

	Cases					
	Valid		Missing		Total	
	N	Percent	N	Percent	N	Percent
BMIGroup * AF	156	100.0%	0	0.0%	156	100.0%
NYHA * AF	108	69.2%	48	30.8%	156	100.0%

BMIGroup * AF

Crosstab

			AF		Total
			No	Yes	
BMIGroup	Underweight	Count	0	1	1
		% within BMIGroup	0.0%	100.0%	100.0%
		% within AF	0.0%	2.6%	0.6%
	Normal	Count	20	9	29
		% within BMIGroup	69.0%	31.0%	100.0%
		% within AF	16.9%	23.7%	18.6%
	Overweight	Count	66	17	83
		% within BMIGroup	79.5%	20.5%	100.0%
		% within AF	55.9%	44.7%	53.2%
	Obese	Count	32	11	43
		% within BMIGroup	74.4%	25.6%	100.0%
		% within AF	27.1%	28.9%	27.6%
Total		Count	118	38	156
		% within BMIGroup	75.6%	24.4%	100.0%
		% within AF	100.0%	100.0%	100.0%

Chi-Square Tests

	Value	df	Asymptotic Significance (2-sided)
Pearson Chi-Square	4.519[a]	3	.211
Likelihood Ratio	4.227	3	.238
Linear-by-Linear Association	.614	1	.433
N of Valid Cases	156		

a. 2 cells (25.0%) have expected count less than 5. The minimum expected count is .24.

NYHA * AF

Crosstab

			AF		Total
			No	Yes	
NYHA	1	Count	43	15	58
		% within NYHA	74.1%	25.9%	100.0%
		% within AF	56.6%	46.9%	53.7%
	2	Count	32	16	48
		% within NYHA	66.7%	33.3%	100.0%
		% within AF	42.1%	50.0%	44.4%
	3	Count	1	1	2
		% within NYHA	50.0%	50.0%	100.0%
		% within AF	1.3%	3.1%	1.9%
Total		Count	76	32	108
		% within NYHA	70.4%	29.6%	100.0%
		% within AF	100.0%	100.0%	100.0%

Chi-Square Tests

	Value	df	Asymptotic Significance (2-sided)
Pearson Chi-Square	1.109[a]	2	.574
Likelihood Ratio	1.077	2	.584
Linear-by-Linear Association	1.032	1	.310
N of Valid Cases	108		

a. 2 cells (33.3%) have expected count less than 5. The minimum expected count is .59.

```
DATASET ACTIVATE DataSet1.

SAVE OUTFILE='C:\Users\user\Downloads\ImAnisah Statistic & SPSS\TOCO T3\Dat
a toco.sav'
  /COMPRESSED.
EXAMINE VARIABLES=LEFTAT_SIZE RIGHTATsize EF BY AF
  /PLOT BOXPLOT STEMLEAF NPPLOT
  /COMPARE GROUPS
  /STATISTICS DESCRIPTIVES
  /CINTERVAL 95
  /MISSING LISTWISE
  /NOTOTAL.
```

Explore

Notes

Output Created		01-MAR-2021 14:39:24
Comments		
Input	Data	C:\Users\user\Downloads\ImAnisah Statistic & SPSS\TOCO T3\Data toco.sav
	Active Dataset	DataSet1
	Filter	<none>
	Weight	<none>
	Split File	<none>
	N of Rows in Working Data File	156
Missing Value Handling	Definition of Missing	User-defined missing values for dependent variables are treated as missing.
	Cases Used	Statistics are based on cases with no missing values for any dependent variable or factor used.
Syntax		EXAMINE VARIABLES=LEFTAT_SIZE RIGHTATsize EF BY AF<br>/PLOT BOXPLOT STEMLEAF NPPLOT<br>/COMPARE GROUPS<br>/STATISTICS DESCRIPTIVES<br>/CINTERVAL 95<br>/MISSING LISTWISE<br>/NOTOTAL.
Resources	Processor Time	00:00:06.44
	Elapsed Time	00:00:06.32

AF

Case Processing Summary

		Cases					
		Valid		Missing		Total	
	AF	N	Percent	N	Percent	N	Percent
LEFTAT_SIZE	No	66	55.9%	52	44.1%	118	100.0%
	Yes	30	78.9%	8	21.1%	38	100.0%
RIGHTATsize	No	66	55.9%	52	44.1%	118	100.0%
	Yes	30	78.9%	8	21.1%	38	100.0%
EF	No	66	55.9%	52	44.1%	118	100.0%
	Yes	30	78.9%	8	21.1%	38	100.0%

Descriptives

	AF			Statistic	Std. Error
LEFTAT_SIZE	No	Mean		18.142	.6128
		95% Confidence Interval for Mean	Lower Bound	16.919	
			Upper Bound	19.366	
		5% Trimmed Mean		17.754	
		Median		17.050	
		Variance		24.787	
		Std. Deviation		4.9787	
		Minimum		11.0	
		Maximum		34.4	
		Range		23.4	
		Interquartile Range		6.2	
		Skewness		1.171	.295
		Kurtosis		1.591	.582
	Yes	Mean		18.837	.7504
		95% Confidence Interval for Mean	Lower Bound	17.302	
			Upper Bound	20.371	
		5% Trimmed Mean		18.957	
		Median		18.600	
		Variance		16.891	
		Std. Deviation		4.1099	
		Minimum		10.0	
		Maximum		26.0	
		Range		16.0	
		Interquartile Range		7.1	
		Skewness		-.305	.427
		Kurtosis		-.288	.833
RIGHTATsize	No	Mean		14.011	.3964
		95% Confidence Interval for Mean	Lower Bound	13.219	
			Upper Bound	14.802	
		5% Trimmed Mean		13.779	
		Median		13.550	
		Variance		10.372	
		Std. Deviation		3.2205	
		Minimum		8.0	
		Maximum		25.0	
		Range		17.0	
		Interquartile Range		4.2	
		Skewness		1.167	.295
		Kurtosis		2.036	.582
	Yes	Mean		14.843	.5780
		95% Confidence Interval for Mean	Lower Bound	13.661	
			Upper Bound	16.025	
		5% Trimmed Mean		14.572	

Descriptives

AF				Statistic	Std. Error
		Median		14.500	
		Variance		10.022	
		Std. Deviation		3.1657	
		Minimum		10.0	
		Maximum		27.3	
		Range		17.3	
		Interquartile Range		3.4	
		Skewness		2.075	.427
		Kurtosis		7.455	.833
EF	No	Mean		49.802	1.1634
		95% Confidence Interval for Mean	Lower Bound	47.478	
			Upper Bound	52.125	
		5% Trimmed Mean		49.951	
		Median		52.500	
		Variance		89.339	
		Std. Deviation		9.4519	
		Minimum		30.0	
		Maximum		67.0	
		Range		37.0	
		Interquartile Range		13.3	
		Skewness		-.366	.295
		Kurtosis		-.778	.582
	Yes	Mean		49.427	1.7542
		95% Confidence Interval for Mean	Lower Bound	45.839	
			Upper Bound	53.014	
		5% Trimmed Mean		49.493	
		Median		50.000	
		Variance		92.317	
		Std. Deviation		9.6081	
		Minimum		34.0	
		Maximum		64.0	
		Range		30.0	
		Interquartile Range		16.0	
		Skewness		-.152	.427
		Kurtosis		-1.235	.833

Tests of Normality

	AF	Kolmogorov-Smirnov[a]			Shapiro-Wilk		
		Statistic	df	Sig.	Statistic	df	Sig.
LEFTAT_SIZE	No	.133	66	.006	.918	66	.000
	Yes	.111	30	.200*	.958	30	.280
RIGHTATsize	No	.123	66	.015	.926	66	.001
	Yes	.214	30	.001	.826	30	.000
EF	No	.132	66	.006	.960	66	.031
	Yes	.120	30	.200*	.939	30	.087

*. This is a lower bound of the true significance.

a. Lilliefors Significance Correction

LEFTAT_SIZE

Stem-and-Leaf Plots

```
LEFTAT_SIZE Stem-and-Leaf Plot for
AF= No

 Frequency    Stem &  Leaf

     2.00       11 .  00
     5.00       12 .  01346
     4.00       13 .  0004
     5.00       14 .  00034
     9.00       15 .  000006669
     4.00       16 .  0004
     6.00       17 .  000019
     7.00       18 .  0000006
     5.00       19 .  00005
     2.00       20 .  00
     4.00       21 .  0037
     2.00       22 .  01
     3.00       23 .  009
     1.00       24 .  0
     2.00       25 .  67
     2.00       26 .  00
      .00       27 .
      .00       28 .
      .00       29 .
     1.00       30 .  0
     2.00 Extremes    (>=33.0)

 Stem width:       1.0
```

```
 Each leaf:        1 case(s)



LEFTAT_SIZE Stem-and-Leaf Plot for
AF= Yes

 Frequency    Stem &  Leaf

     2.00        1 .  00
     1.00        1 .  2
     4.00        1 .  5555
     4.00        1 .  6777
     6.00        1 .  888899
     5.00        2 .  00001
     3.00        2 .  333
     4.00        2 .  4444
     1.00        2 .  6

 Stem width:      10.0
 Each leaf:        1 case(s)
```

Normal Q-Q Plots

Normal Q-Q Plot of LEFTAT_SIZE

for AF= No

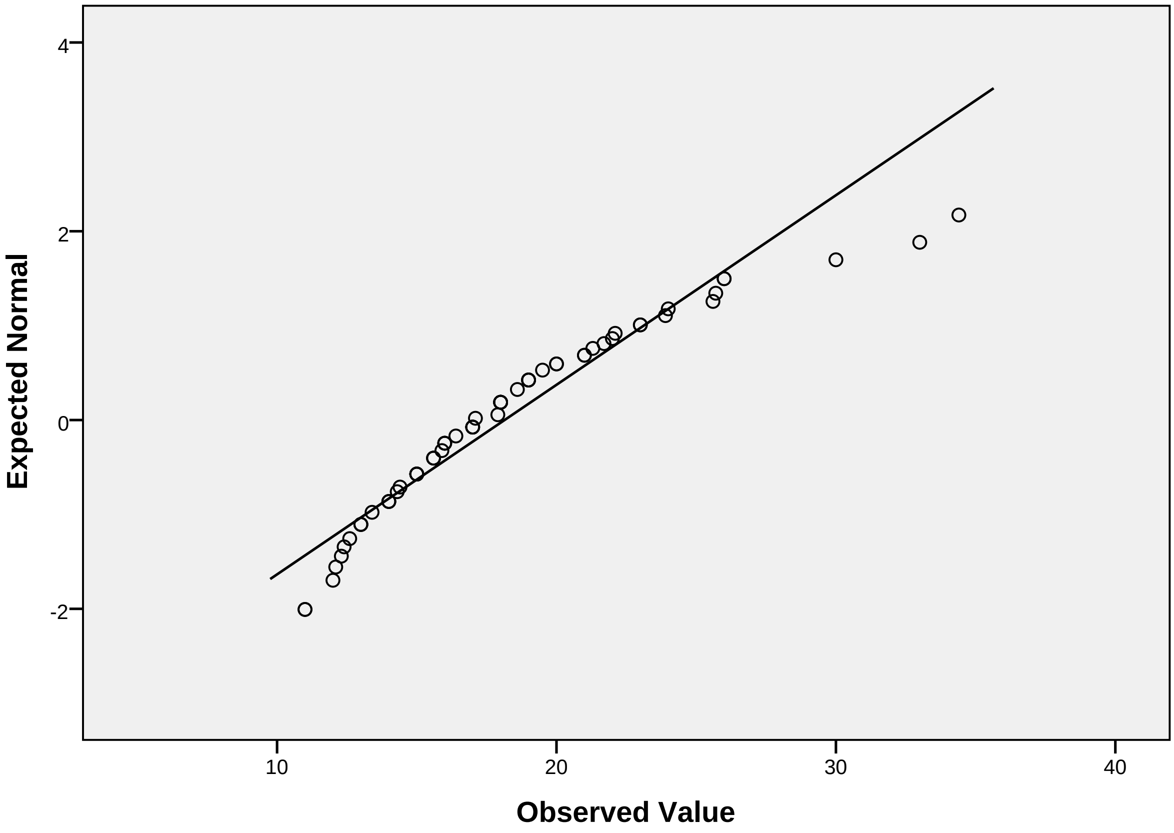

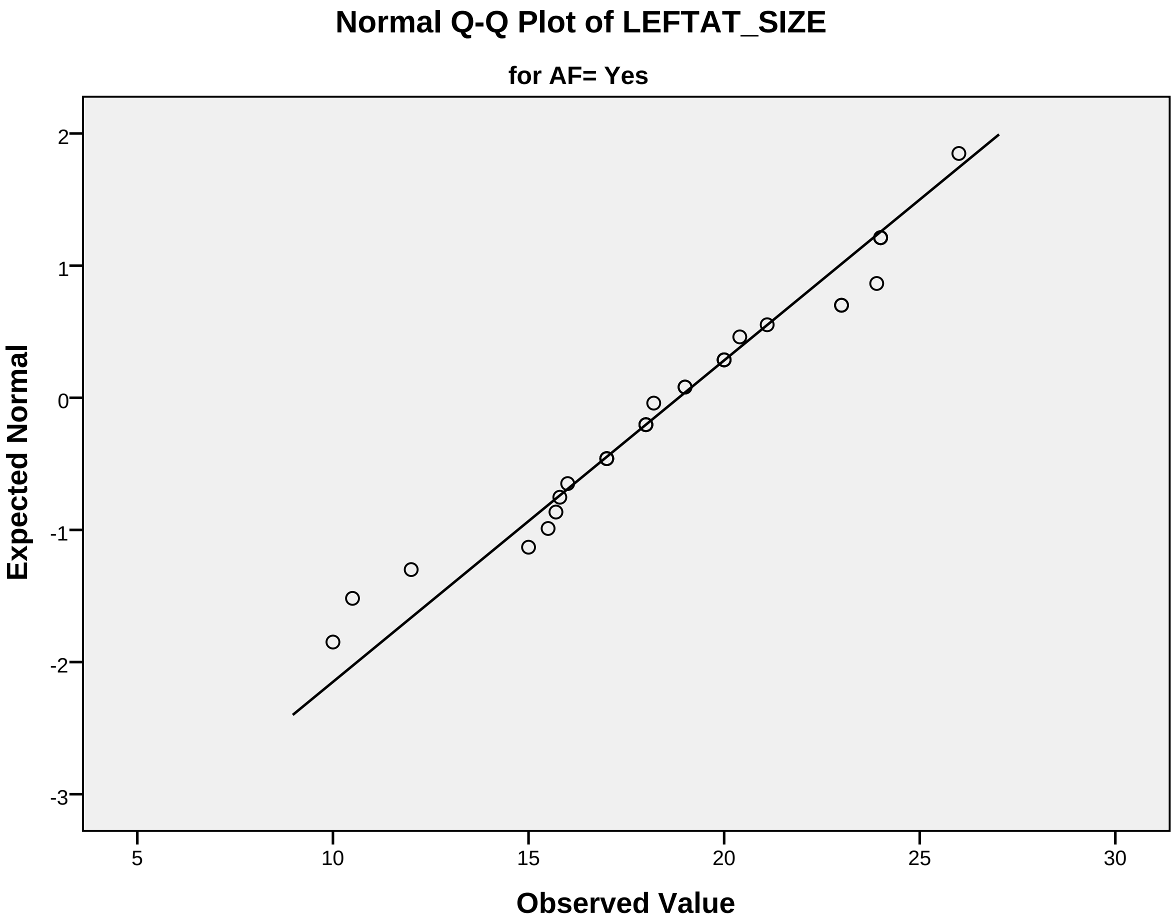

Detrended Normal Q-Q Plots

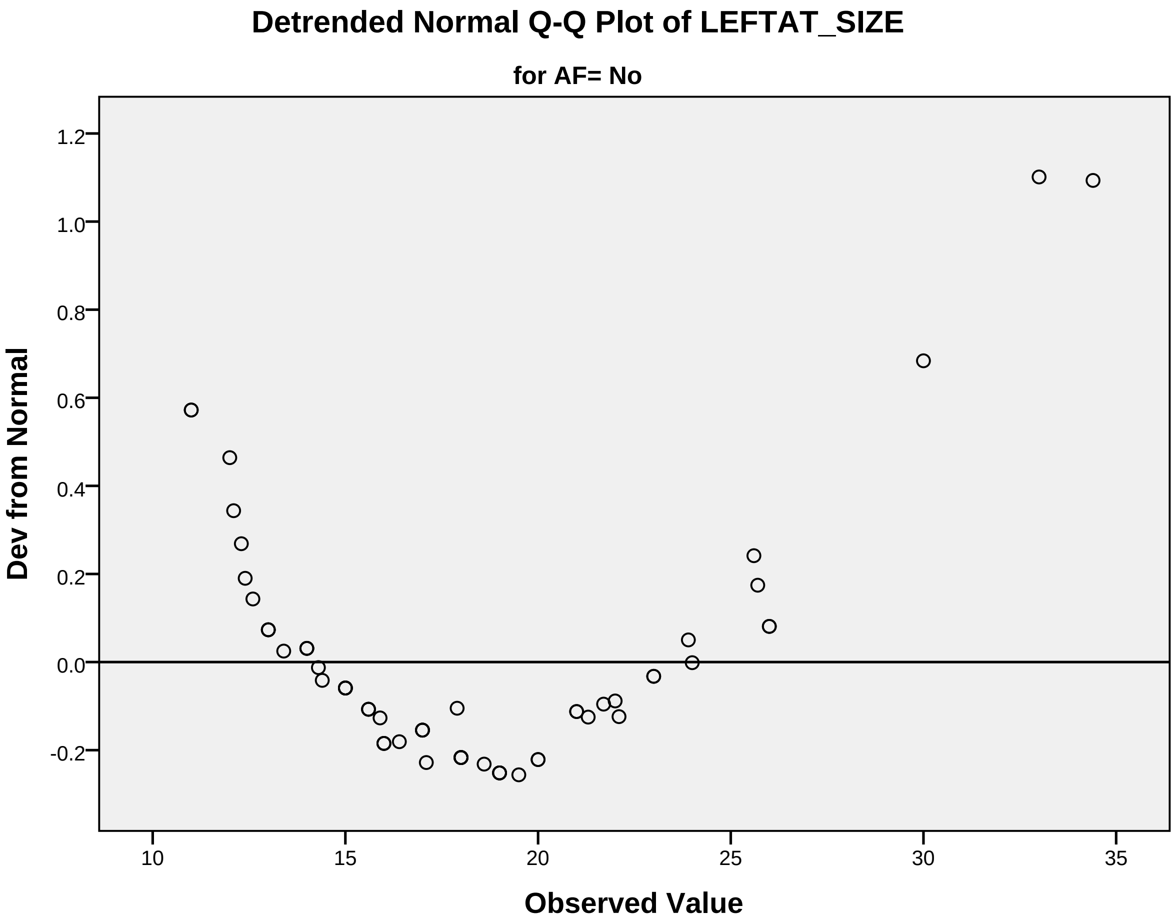
Detrended Normal Q-Q Plot of LEFTAT_SIZE
for AF= No
Dev from Normal
1.2
1.0
0.8
0.6
0.4
0.2
0.0
-0.2
10
15
20
25
30
35
Observed Value

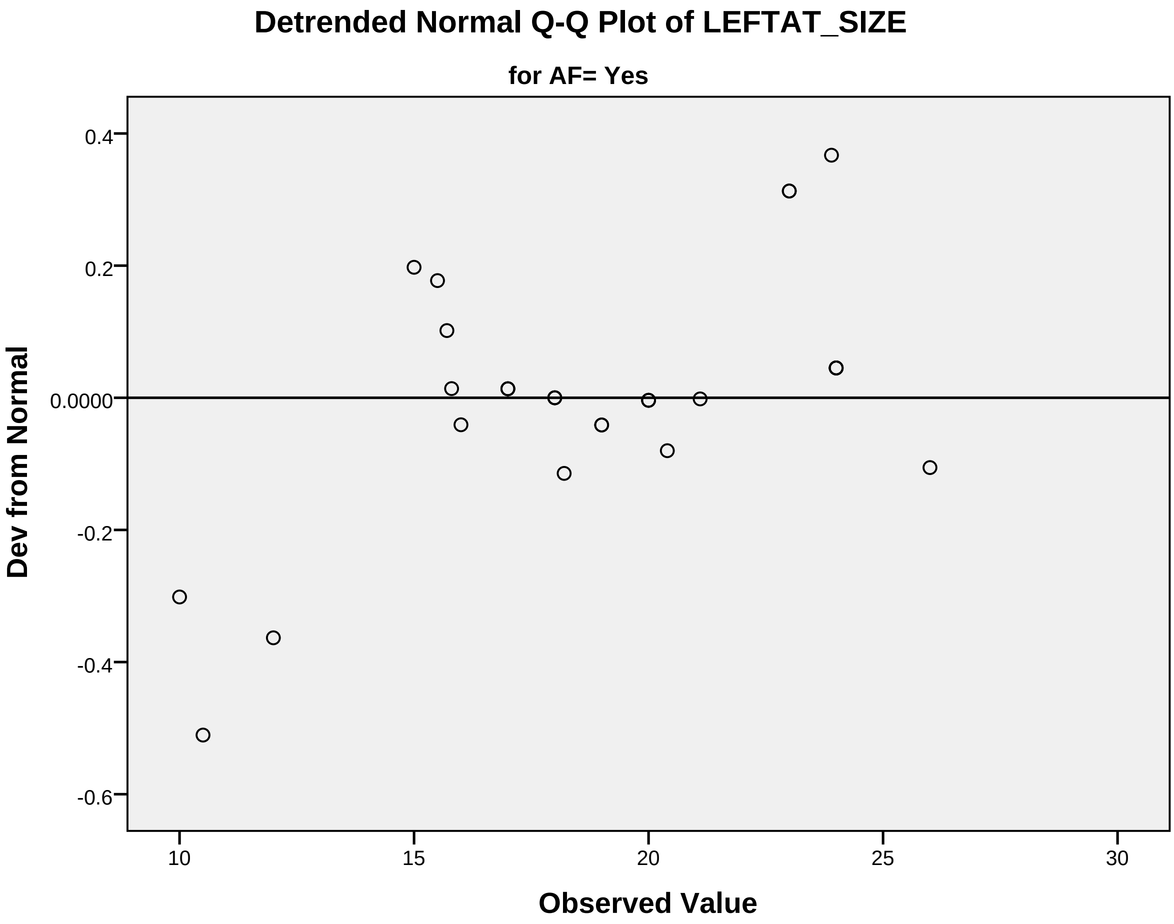
Detrended Normal Q-Q Plot of LEFTAT_SIZE
for AF= Yes
Dev from Normal
0.4
0.2
0.0000
-0.2
-0.4
-0.6
10
15
20
25
30
Observed Value

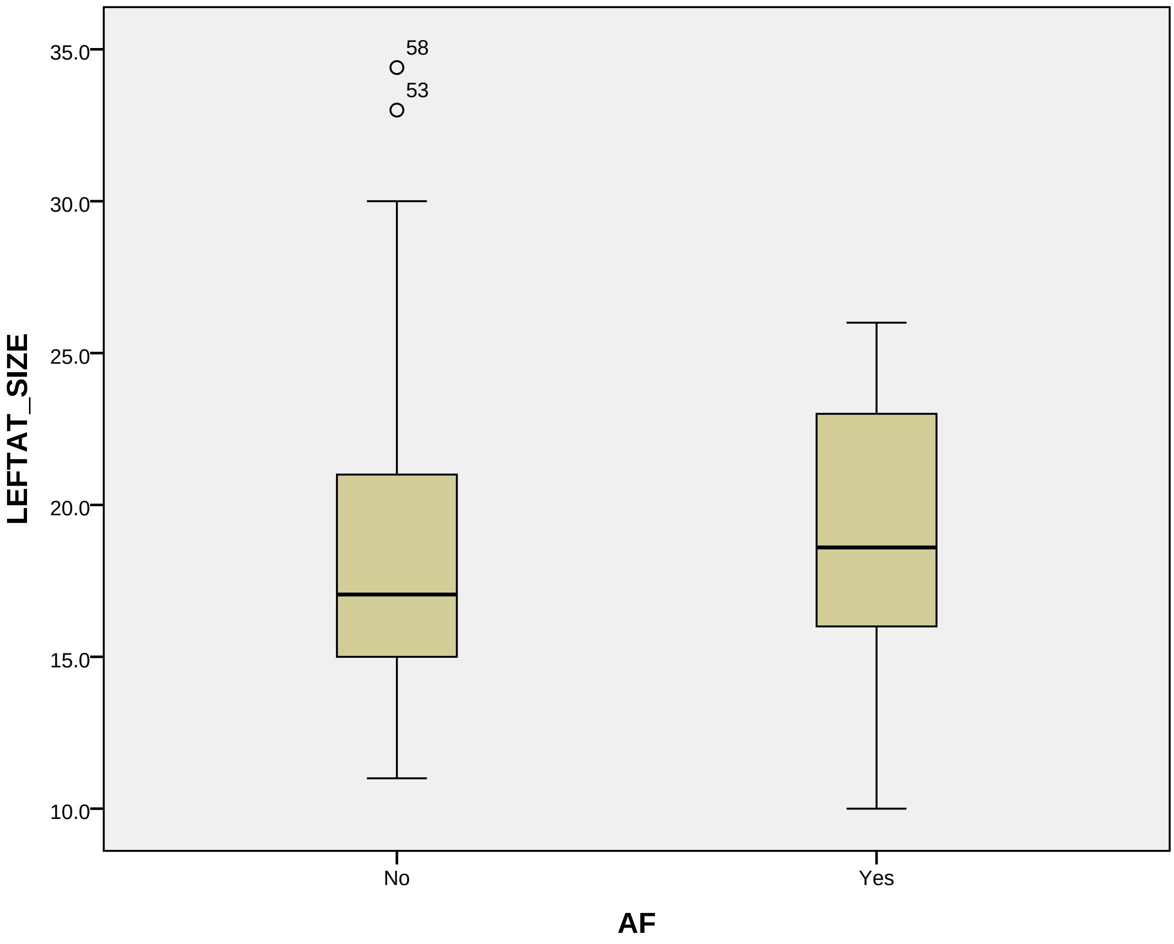

RIGHTATsize

Stem-and-Leaf Plots

```
RIGHTATsize Stem-and-Leaf Plot for
AF= No

 Frequency    Stem &  Leaf

     1.00        8 .  0
     2.00        9 .  89
     3.00       10 .  003
    11.00       11 .  00000035678
    10.00       12 .  0000003459
     7.00       13 .  0000447
     9.00       14 .  000000078
     6.00       15 .  000025
     6.00       16 .  000004
     4.00       17 .  0000
     1.00       18 .  0
     3.00       19 .  045
```

```
    1.00       20 .  7
    2.00 Extremes    (>=24.0)

Stem width:       1.0
Each leaf:        1 case(s)
```

```
RIGHTATsize Stem-and-Leaf Plot for
AF= Yes

 Frequency    Stem &  Leaf

     1.00       10 .  0
     1.00       11 .  1
     5.00       12 .  00004
     3.00       13 .  009
     5.00       14 .  00000
     7.00       15 .  0000000
     2.00       16 .  09
     2.00       17 .  07
     3.00       18 .  000
     1.00 Extremes    (>=27.3)

 Stem width:       1.0
 Each leaf:        1 case(s)
```

Normal Q-Q Plots

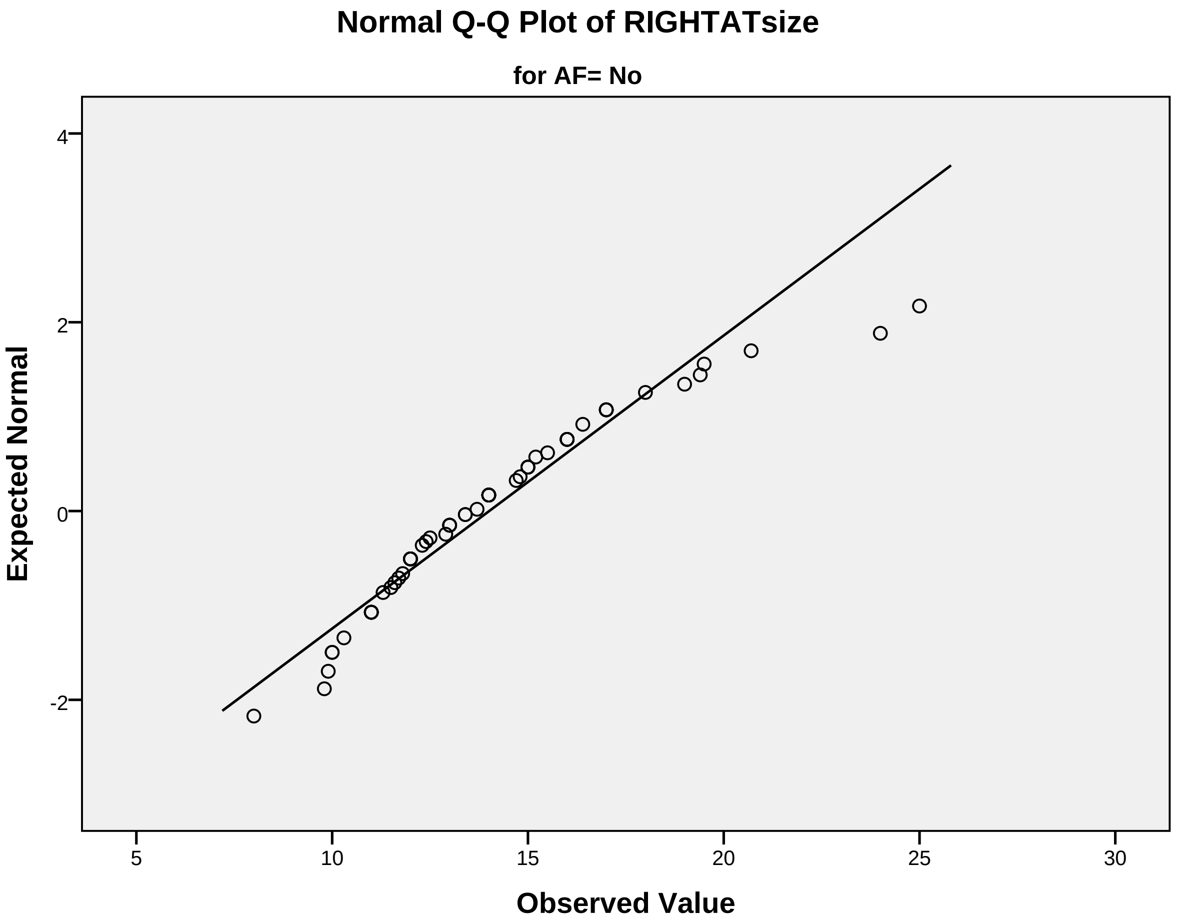

Normal Q-Q Plot of RIGHTATsize
for AF= No
Expected Normal
4
2
0
-2
5
10
15
20
25
30
Observed Value

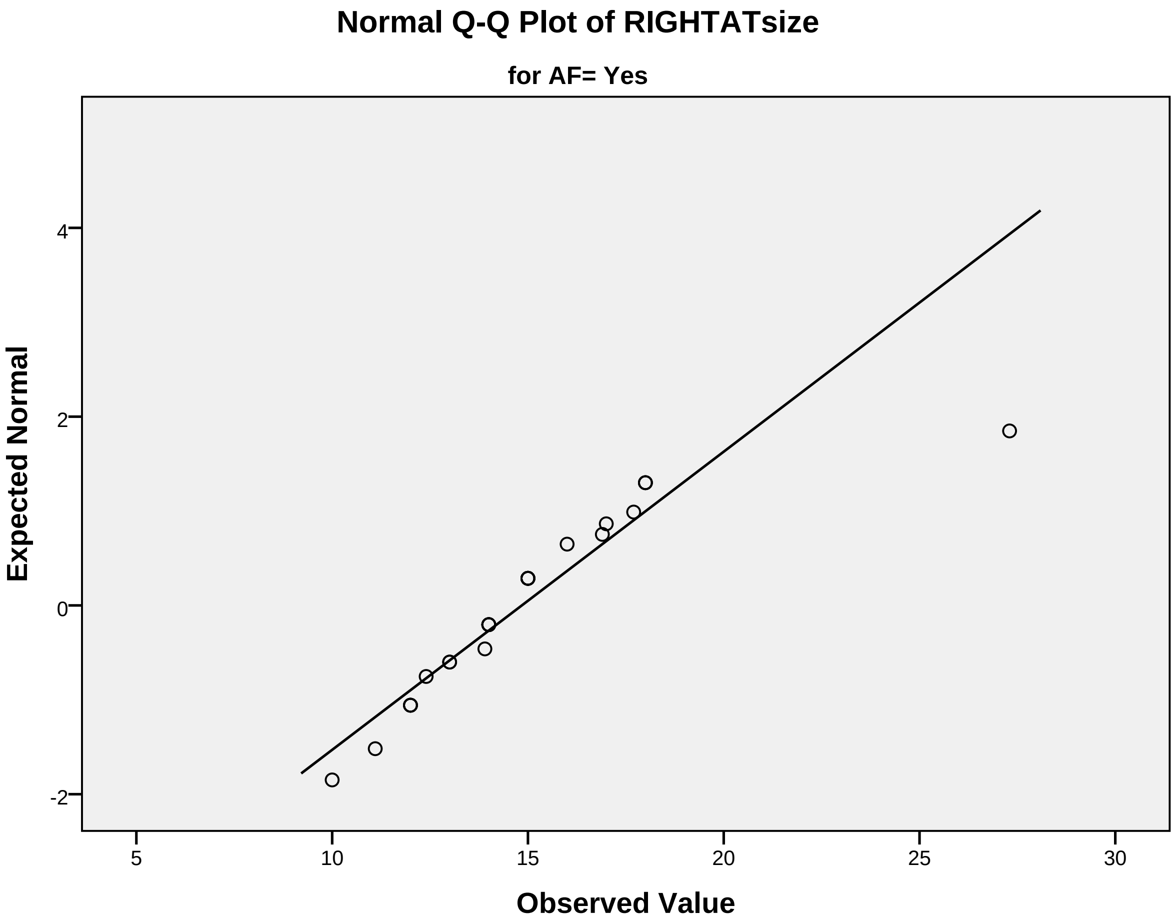

Detrended Normal Q-Q Plots

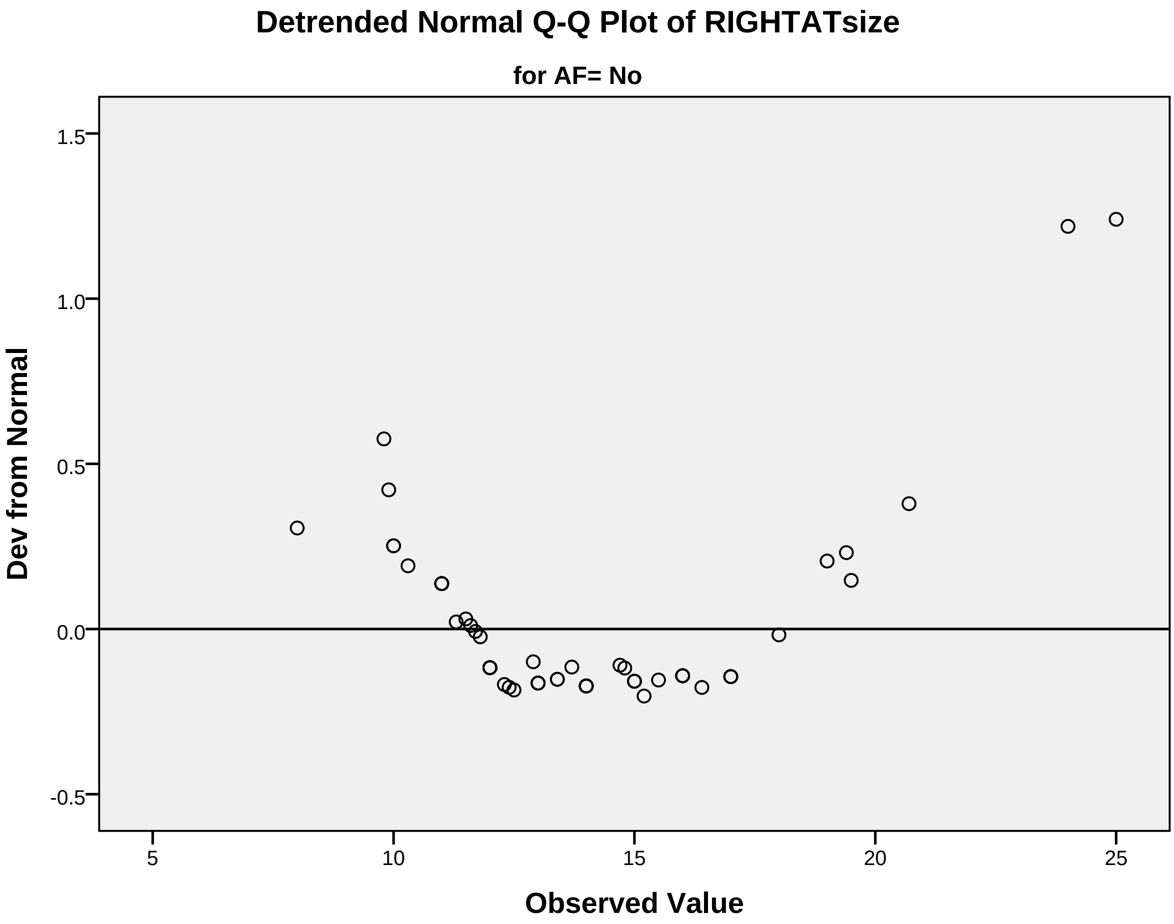

Detrended Normal Q-Q Plot of RIGHTATsize
for AF= No
Dev from Normal
1.5
1.0
0.5
0.0
-0.5
5
10
15
20
25
Observed Value

Detrended Normal Q-Q Plot of RIGHTATsize

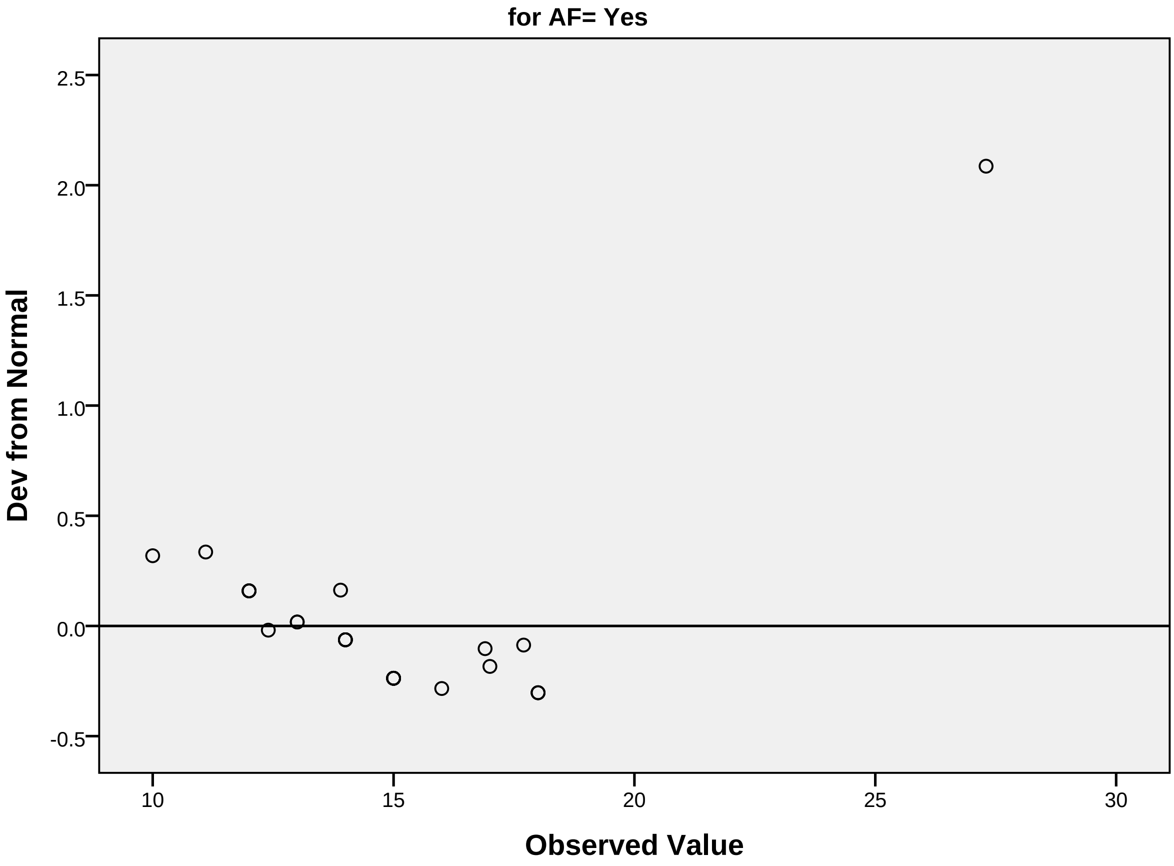

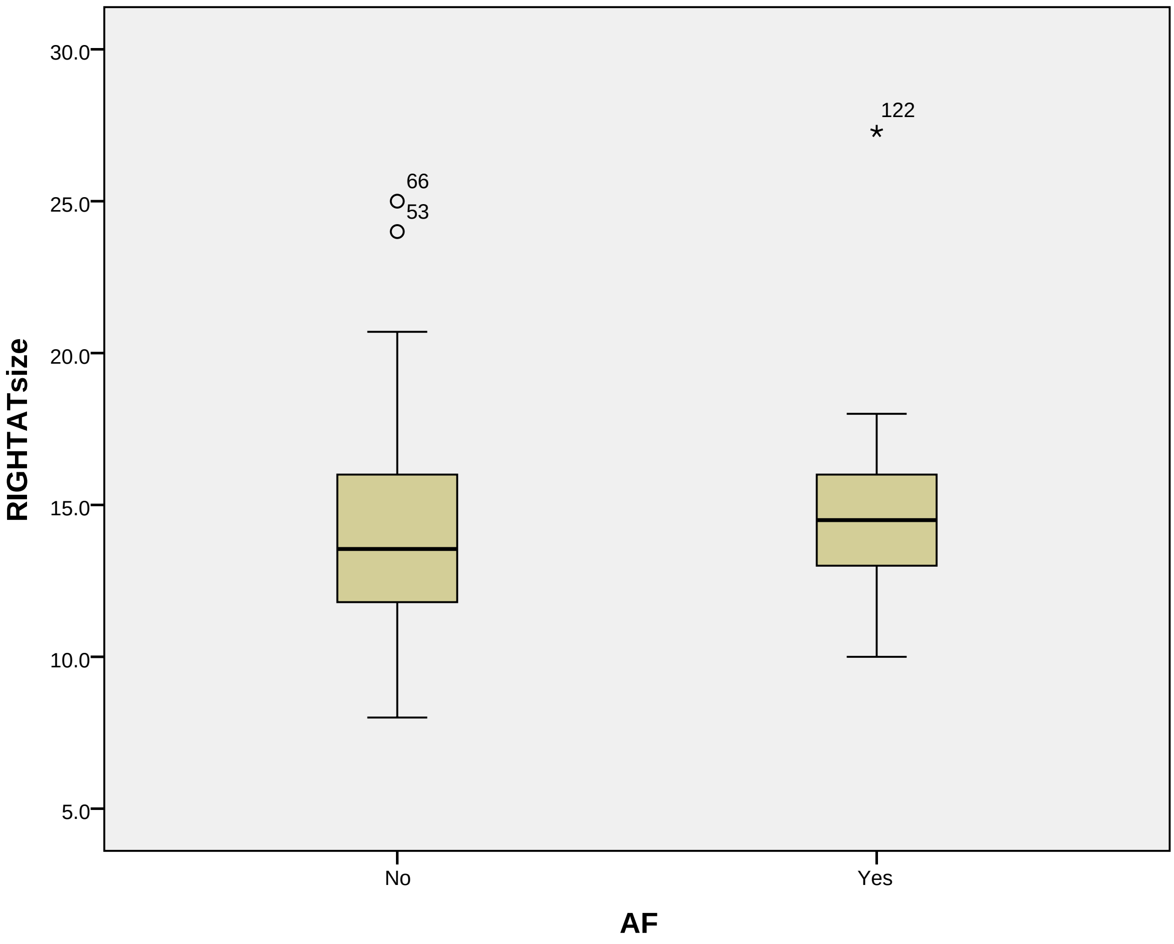

EF

Stem-and-Leaf Plots

```
EF Stem-and-Leaf Plot for
AF= No

 Frequency    Stem &  Leaf

     2.00        3 .  01
     2.00        3 .  23
     3.00        3 .  455
     2.00        3 .  67
     4.00        3 .  8999
     1.00        4 .  1
     2.00        4 .  23
     7.00        4 .  4455555
     4.00        4 .  6777
     3.00        4 .  888
     2.00        5 .  01
     5.00        5 .  23333
```

```
     5.00        5 .  44555
    11.00        5 .  66666777777
     3.00        5 .  888
     5.00        6 .  00001
     2.00        6 .  23
     1.00        6 .  5
     2.00        6 .  67

 Stem width:      10.0
 Each leaf:        1 case(s)
```

```
EF Stem-and-Leaf Plot for
AF= Yes

 Frequency    Stem &  Leaf

     2.00        3 .  44
     5.00        3 .  66779
     4.00        4 .  2344
     3.00        4 .  789
     5.00        5 .  00223
     6.00        5 .  667789
     5.00        6 .  02334

 Stem width:      10.0
 Each leaf:        1 case(s)
```

Normal Q-Q Plots

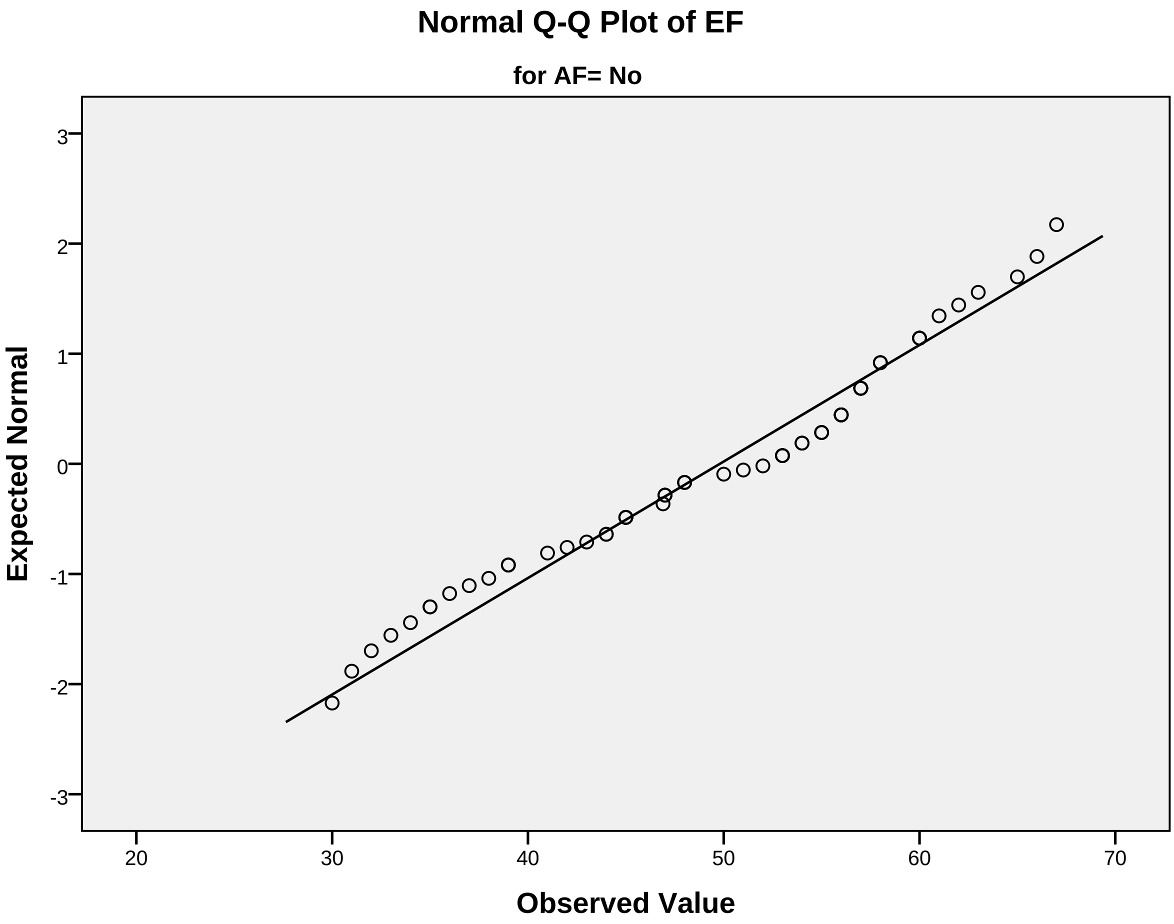
Normal Q-Q Plot of EF
for AF= No
Expected Normal
Observed Value
3
2
1
0
-1
-2
-3
20
30
40
50
60
70

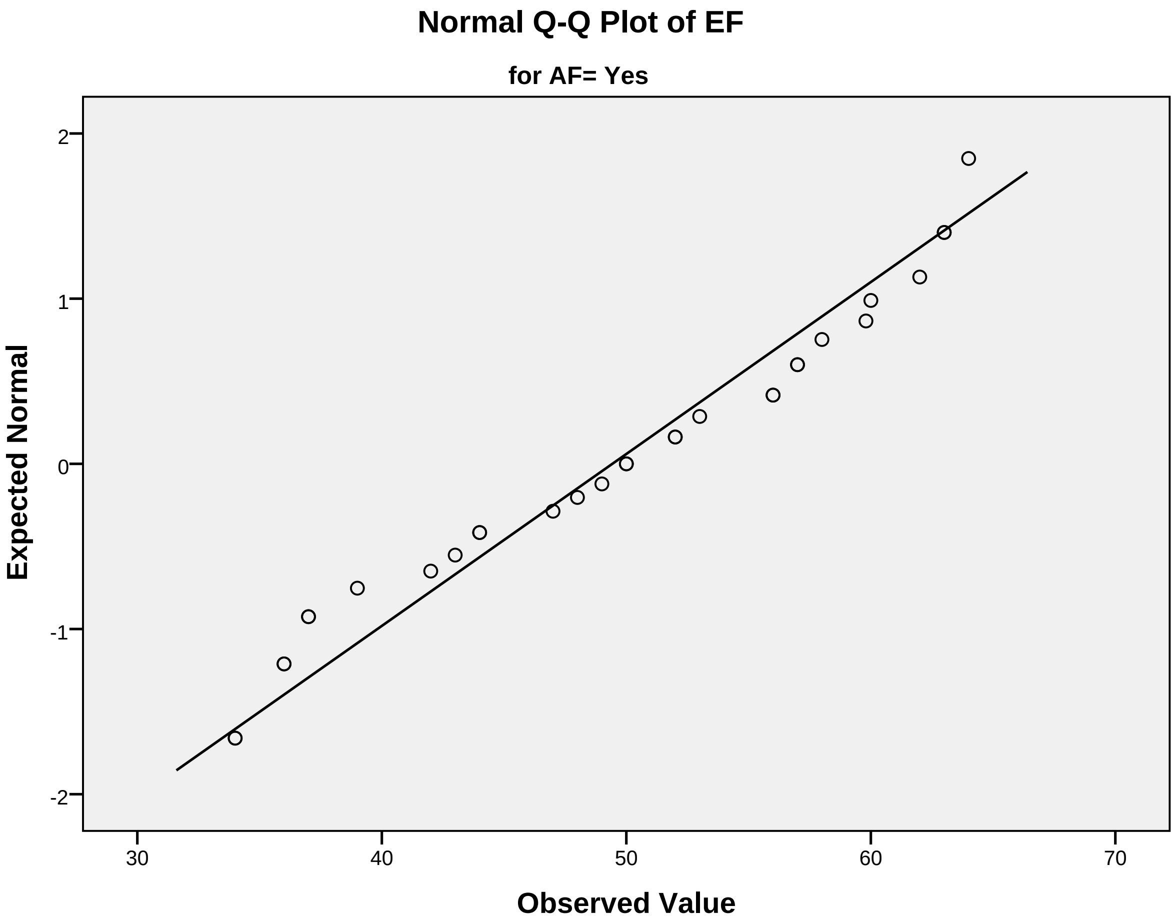

Detrended Normal Q-Q Plots

Detrended Normal Q-Q Plot of EF

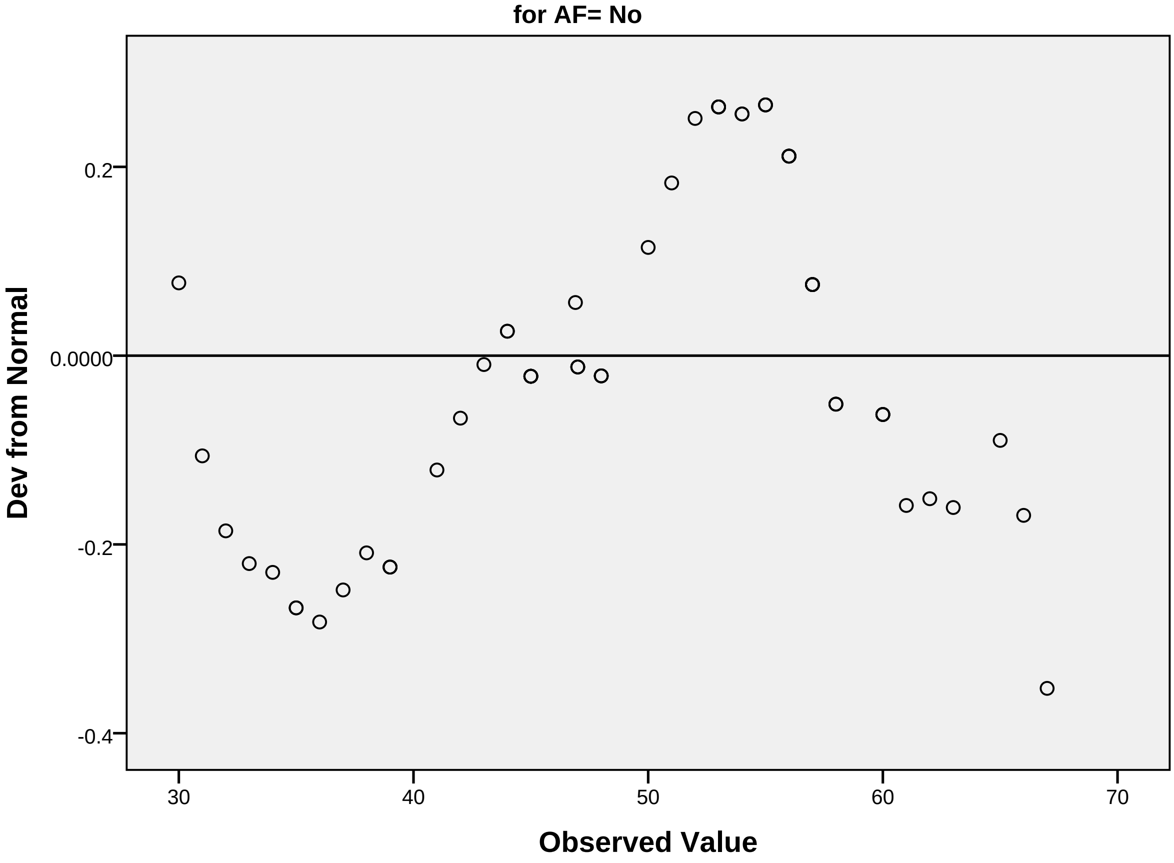

Detrended Normal Q-Q Plot of EF

for AF= Yes

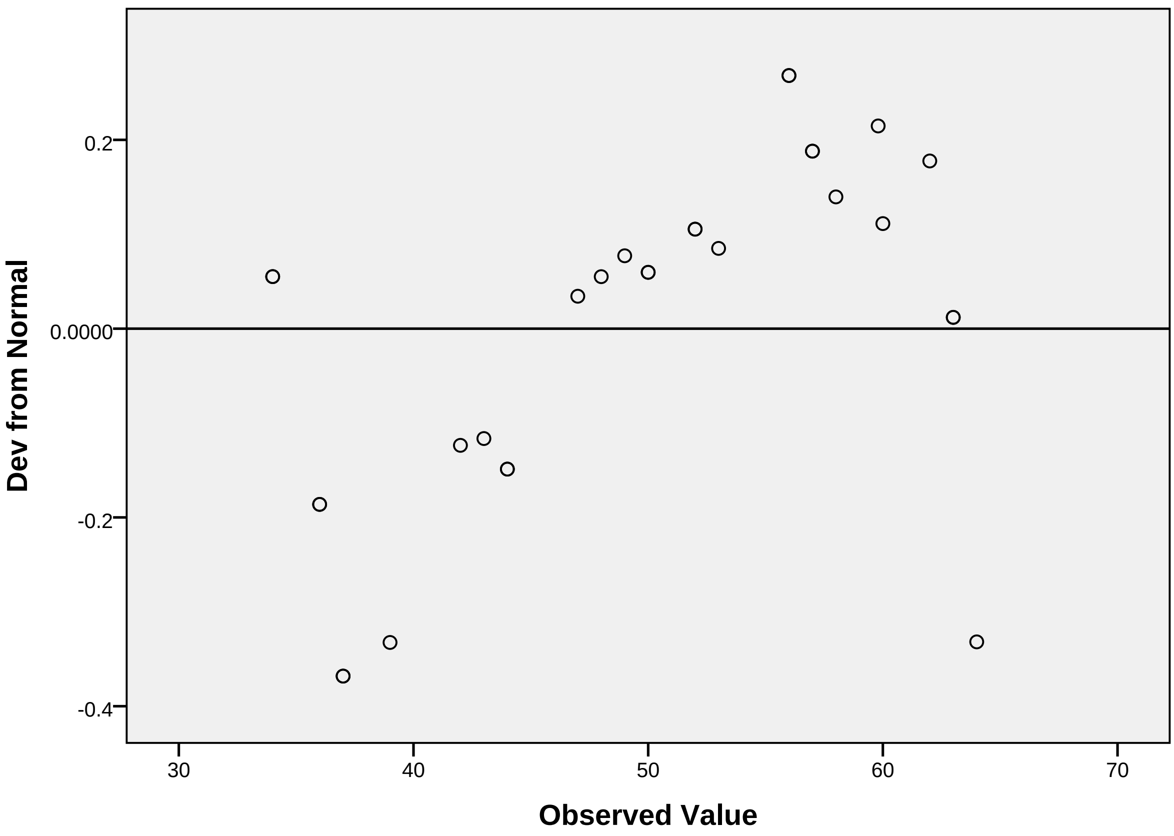

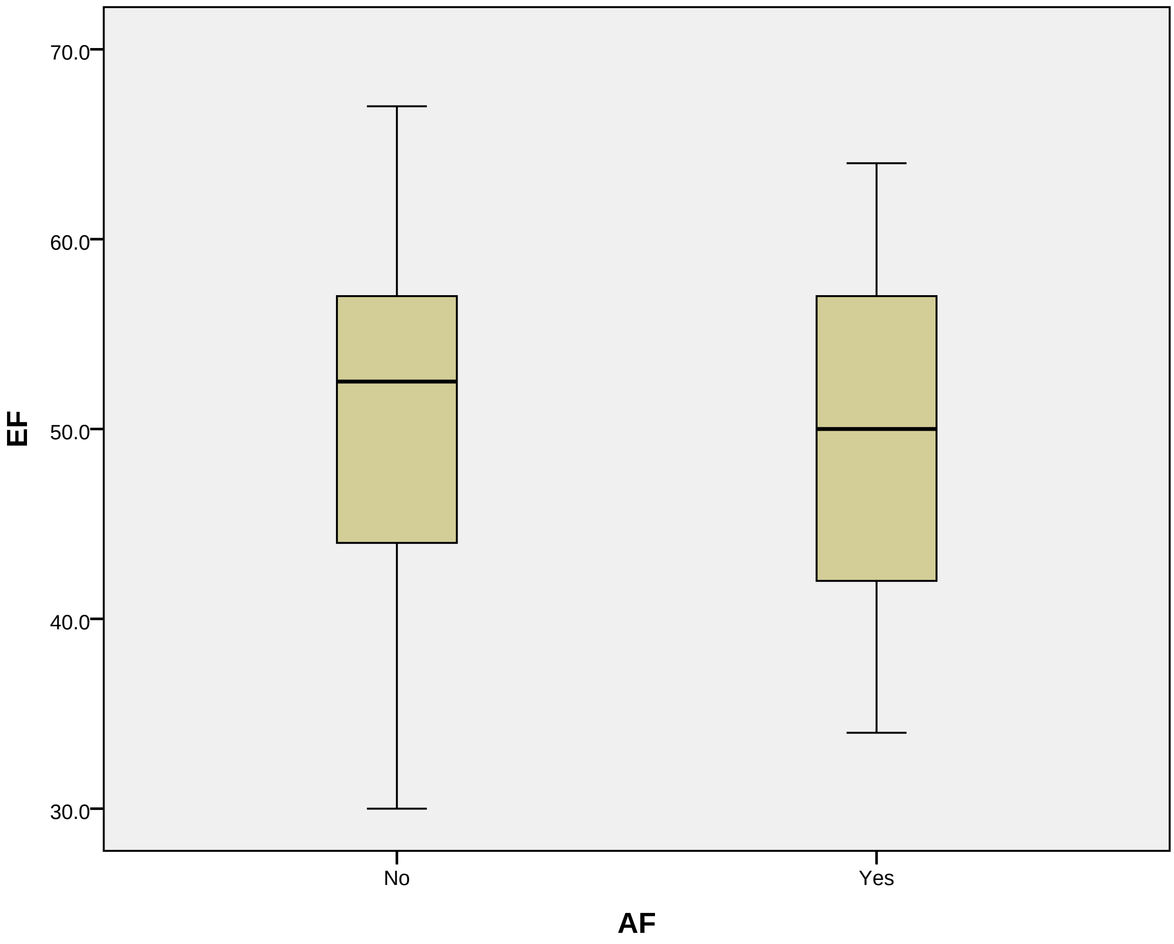


```
T-TEST GROUPS=AF(0 1)
 /MISSING=ANALYSIS
 /VARIABLES=EF
 /CRITERIA=CI(.95).
```

T-Test

Notes

Output Created		01-MAR-2021 14:39:59
Comments		
Input	Data	C:\Users\user\Downloads\ImAnisah Statistic & SPSS\TOCO T3\Data toco.sav
	Active Dataset	DataSet1
	Filter	<none>
	Weight	<none>
	Split File	<none>
	N of Rows in Working Data File	156
Missing Value Handling	Definition of Missing	User defined missing values are treated as missing.
	Cases Used	Statistics for each analysis are based on the cases with no missing or out-of-range data for any variable in the analysis.
Syntax		T-TEST GROUPS=AF(0 1) /MISSING=ANALYSIS /VARIABLES=EF /CRITERIA=CI(.95).
Resources	Processor Time	00:00:00.00
	Elapsed Time	00:00:00.02

Group Statistics

	AF	N	Mean	Std. Deviation	Std. Error Mean
EF	No	78	50.255	9.3082	1.0540
	Yes	33	48.903	9.7985	1.7057

Independent Samples Test

		Levene's Test for Equality of Variances		t-test for Equality of Means						
									95% Confidence Interval of the Difference	
		F	Sig.	t	df	Sig. (2-tailed)	Mean Difference	Std. Error Difference	Lower	Upper
EF	Equal variances assumed	.078	.780	.689	109	.493	1.3521	1.9634	-2.5393	5.2435
	Equal variances not assumed			.674	57.610	.503	1.3521	2.0050	-2.6620	5.3662

```
NPAR TESTS
 /M-W= RIGHTATsize LEFTAT_SIZE BY AF(0 1)
 /STATISTICS=DESCRIPTIVES
 /MISSING ANALYSIS.
```

NPar Tests

Notes

Output Created		01-MAR-2021 14:40:19
Comments		
Input	Data	C:\Users\user\Downloads\ImAnisah Statistic & SPSS\TOCO T3\Data toco.sav
	Active Dataset	DataSet1
	Filter	<none>
	Weight	<none>
	Split File	<none>
	N of Rows in Working Data File	156
Missing Value Handling	Definition of Missing	User-defined missing values are treated as missing.
	Cases Used	Statistics for each test are based on all cases with valid data for the variable(s) used in that test.
Syntax		NPAR TESTS<br>/M-W= RIGHTATsize LEFTAT_SIZE BY AF(0 1)<br>/STATISTICS=DESCRIPTIVES<br>/MISSING ANALYSIS.
Resources	Processor Time	00:00:00.03
	Elapsed Time	00:00:00.02
	Number of Cases Allowed[a]	393216

a. Based on availability of workspace memory.

Descriptive Statistics

	N	Mean	Std. Deviation	Minimum	Maximum
RIGHTATsize	96	14.271	3.2103	8.0	27.3
LEFTAT_SIZE	100	18.615	5.1879	10.0	39.0
AF	156	.24	.431	0	1

Mann-Whitney Test

Ranks

	AF	N	Mean Rank	Sum of Ranks
RIGHTATsize	No	66	45.59	3009.00
	Yes	30	54.90	1647.00
	Total	96		
LEFTAT_SIZE	No	69	47.53	3279.50
	Yes	31	57.11	1770.50
	Total	100		

Test Statistics[a]

	RIGHTATsize	LEFTAT_SIZE
Mann-Whitney U	798.000	864.500
Wilcoxon W	3009.000	3279.500
Z	-1.522	-1.529
Asymp. Sig. (2-tailed)	.128	.126

a. Grouping Variable: AF

```
CROSSTABS
 /TABLES=DM HPT ASTHMA COPD CKD HYPERCHOL Smokergrp alcoholgrp BY AF
 /FORMAT=AVALUE TABLES
 /STATISTICS=CHISQ
 /CELLS=COUNT ROW COLUMN
 /COUNT ROUND CELL.
```

Crosstabs

Notes

Output Created		01-MAR-2021 14:42:19
Comments		
Input	Data	C:\Users\user\Downloads\ImAnisah Statistic & SPSS\TOCO T3\Data toco.sav
	Active Dataset	DataSet1
	Filter	<none>
	Weight	<none>
	Split File	<none>
	N of Rows in Working Data File	156
Missing Value Handling	Definition of Missing	User-defined missing values are treated as missing.
	Cases Used	Statistics for each table are based on all the cases with valid data in the specified range(s) for all variables in each table.
Syntax		CROSSTABS /TABLES=DM HPT ASTHMA COPD CKD HYPERCHOL Smokergrp alcoholgrp BY AF /FORMAT=AVALUE TABLES /STATISTICS=CHISQ /CELLS=COUNT ROW COLUMN /COUNT ROUND CELL.
Resources	Processor Time	00:00:00.03
	Elapsed Time	00:00:00.06

Notes

Dimensions Requested	2
Cells Available	524245

Case Processing Summary

	Cases					
	Valid		Missing		Total	
	N	Percent	N	Percent	N	Percent
DM * AF	117	75.0%	39	25.0%	156	100.0%
HPT * AF	116	74.4%	40	25.6%	156	100.0%
ASTHMA * AF	116	74.4%	40	25.6%	156	100.0%
COPD * AF	116	74.4%	40	25.6%	156	100.0%
CKD * AF	115	73.7%	41	26.3%	156	100.0%
HYPERCHOL * AF	114	73.1%	42	26.9%	156	100.0%
Smokergrp * AF	107	68.6%	49	31.4%	156	100.0%
alcoholgrp * AF	99	63.5%	57	36.5%	156	100.0%

DM * AF

Crosstab

			AF		Total
			No	Yes	
DM	No	Count	22	13	35
		% within DM	62.9%	37.1%	100.0%
		% within AF	27.2%	36.1%	29.9%
	Yes	Count	59	23	82
		% within DM	72.0%	28.0%	100.0%
		% within AF	72.8%	63.9%	70.1%
Total		Count	81	36	117
		% within DM	69.2%	30.8%	100.0%
		% within AF	100.0%	100.0%	100.0%

Chi-Square Tests

	Value	df	Asymptotic Significance (2-sided)	Exact Sig. (2-sided)	Exact Sig. (1-sided)
Pearson Chi-Square	.952[a]	1	.329		
Continuity Correction[b]	.573	1	.449		
Likelihood Ratio	.935	1	.334		
Fisher's Exact Test				.384	.223
Linear-by-Linear Association	.944	1	.331		
N of Valid Cases	117				

a. 0 cells (0.0%) have expected count less than 5. The minimum expected count is 10.77.

b. Computed only for a 2x2 table

HPT * AF

Crosstab

			AF		Total
			No	Yes	
HPT	No	Count	14	6	20
		% within HPT	70.0%	30.0%	100.0%
		% within AF	17.3%	17.1%	17.2%
	Yes	Count	67	29	96
		% within HPT	69.8%	30.2%	100.0%
		% within AF	82.7%	82.9%	82.8%
Total		Count	81	35	116
		% within HPT	69.8%	30.2%	100.0%
		% within AF	100.0%	100.0%	100.0%

Chi-Square Tests

	Value	df	Asymptotic Significance (2-sided)	Exact Sig. (2-sided)	Exact Sig. (1-sided)
Pearson Chi-Square	.000[a]	1	.985		
Continuity Correction[b]	.000	1	1.000		
Likelihood Ratio	.000	1	.985		
Fisher's Exact Test				1.000	.607
Linear-by-Linear Association	.000	1	.985		
N of Valid Cases	116				

a. 0 cells (0.0%) have expected count less than 5. The minimum expected count is 6.03.

b. Computed only for a 2x2 table

ASTHMA * AF

Crosstab

			AF		
			No	Yes	Total
ASTHMA	No	Count	80	35	115
		% within ASTHMA	69.6%	30.4%	100.0%
		% within AF	98.8%	100.0%	99.1%
	Yes	Count	1	0	1
		% within ASTHMA	100.0%	0.0%	100.0%
		% within AF	1.2%	0.0%	0.9%
Total		Count	81	35	116
		% within ASTHMA	69.8%	30.2%	100.0%
		% within AF	100.0%	100.0%	100.0%

Chi-Square Tests

	Value	df	Asymptotic Significance (2-sided)	Exact Sig. (2-sided)	Exact Sig. (1-sided)
Pearson Chi-Square	.436[a]	1	.509		
Continuity Correction[b]	.000	1	1.000		
Likelihood Ratio	.722	1	.395		
Fisher's Exact Test				1.000	.698
Linear-by-Linear Association	.432	1	.511		
N of Valid Cases	116				

a. 2 cells (50.0%) have expected count less than 5. The minimum expected count is .30.

b. Computed only for a 2x2 table

COPD * AF

Crosstab

			AF		
			No	Yes	Total
COPD	No	Count	79	35	114
		% within COPD	69.3%	30.7%	100.0%
		% within AF	97.5%	100.0%	98.3%
	Yes	Count	2	0	2
		% within COPD	100.0%	0.0%	100.0%
		% within AF	2.5%	0.0%	1.7%
Total		Count	81	35	116
		% within COPD	69.8%	30.2%	100.0%
		% within AF	100.0%	100.0%	100.0%

Chi-Square Tests

	Value	df	Asymptotic Significance (2-sided)	Exact Sig. (2-sided)	Exact Sig. (1-sided)
Pearson Chi-Square	.879[a]	1	.348		
Continuity Correction[b]	.026	1	.872		
Likelihood Ratio	1.452	1	.228		
Fisher's Exact Test				1.000	.486
Linear-by-Linear Association	.872	1	.350		
N of Valid Cases	116				

a. 2 cells (50.0%) have expected count less than 5. The minimum expected count is .60.

b. Computed only for a 2x2 table

CKD * AF

Crosstab

			AF		Total
			No	Yes	
CKD	No	Count	70	30	100
		% within CKD	70.0%	30.0%	100.0%
		% within AF	87.5%	85.7%	87.0%
	Yes	Count	10	5	15
		% within CKD	66.7%	33.3%	100.0%
		% within AF	12.5%	14.3%	13.0%
Total		Count	80	35	115
		% within CKD	69.6%	30.4%	100.0%
		% within AF	100.0%	100.0%	100.0%

Chi-Square Tests

	Value	df	Asymptotic Significance (2-sided)	Exact Sig. (2-sided)	Exact Sig. (1-sided)
Pearson Chi-Square	.068[a]	1	.794		
Continuity Correction[b]	.000	1	1.000		
Likelihood Ratio	.067	1	.795		
Fisher's Exact Test				.771	.504
Linear-by-Linear Association	.068	1	.794		
N of Valid Cases	115				

a. 1 cells (25.0%) have expected count less than 5. The minimum expected count is 4.57.

b. Computed only for a 2x2 table

HYPERCHOL * AF

Crosstab

			AF		Total
			No	Yes	
HYPERCHOL	No	Count	10	7	17
		% within HYPERCHOL	58.8%	41.2%	100.0%
		% within AF	12.7%	20.0%	14.9%
	Yes	Count	69	28	97
		% within HYPERCHOL	71.1%	28.9%	100.0%
		% within AF	87.3%	80.0%	85.1%
Total		Count	79	35	114
		% within HYPERCHOL	69.3%	30.7%	100.0%
		% within AF	100.0%	100.0%	100.0%

Chi-Square Tests

	Value	df	Asymptotic Significance (2-sided)	Exact Sig. (2-sided)	Exact Sig. (1-sided)
Pearson Chi-Square	1.030[a]	1	.310		
Continuity Correction[b]	.533	1	.465		
Likelihood Ratio	.988	1	.320		
Fisher's Exact Test				.393	.229
Linear-by-Linear Association	1.021	1	.312		
N of Valid Cases	114				

a. 0 cells (0.0%) have expected count less than 5. The minimum expected count is 5.22.

b. Computed only for a 2x2 table

Smokergrp * AF

Crosstab

			AF		Total
			No	Yes	
Smokergrp	No	Count	38	6	44
		% within Smokergrp	86.4%	13.6%	100.0%
		% within AF	48.1%	21.4%	41.1%
	Yes	Count	41	22	63
		% within Smokergrp	65.1%	34.9%	100.0%
		% within AF	51.9%	78.6%	58.9%
Total		Count	79	28	107
		% within Smokergrp	73.8%	26.2%	100.0%
		% within AF	100.0%	100.0%	100.0%

Chi-Square Tests

	Value	df	Asymptotic Significance (2-sided)	Exact Sig. (2-sided)	Exact Sig. (1-sided)
Pearson Chi-Square	6.074[a]	1	.014		
Continuity Correction[b]	5.023	1	.025		
Likelihood Ratio	6.442	1	.011		
Fisher's Exact Test				.015	.011
Linear-by-Linear Association	6.018	1	.014		
N of Valid Cases	107				

a. 0 cells (0.0%) have expected count less than 5. The minimum expected count is 11.51.

b. Computed only for a 2x2 table

alcoholgrp * AF

Crosstab

			AF		Total
			No	Yes	
alcoholgrp	No	Count	70	23	93
		% within alcoholgrp	75.3%	24.7%	100.0%
		% within AF	95.9%	88.5%	93.9%
	Yes	Count	3	3	6
		% within alcoholgrp	50.0%	50.0%	100.0%
		% within AF	4.1%	11.5%	6.1%
Total		Count	73	26	99
		% within alcoholgrp	73.7%	26.3%	100.0%
		% within AF	100.0%	100.0%	100.0%

Chi-Square Tests

	Value	df	Asymptotic Significance (2-sided)	Exact Sig. (2-sided)	Exact Sig. (1-sided)
Pearson Chi-Square	1.858[a]	1	.173		
Continuity Correction[b]	.783	1	.376		
Likelihood Ratio	1.646	1	.199		
Fisher's Exact Test				.184	.184
Linear-by-Linear Association	1.840	1	.175		
N of Valid Cases	99				

a. 2 cells (50.0%) have expected count less than 5. The minimum expected count is 1.58.

b. Computed only for a 2x2 table

```
FREQUENCIES VARIABLES=ISOLATED
  /NTILES=4
  /STATISTICS=STDDEV MEAN
```

Frequencies

Notes

Output Created		01-MAR-2021 14:44:40
Comments		
Input	Data	C:\Users\user\Downloads\ImAnisah Statistic & SPSS\TOCO T3\Data toco.sav
	Active Dataset	DataSet1
	Filter	<none>
	Weight	<none>
	Split File	<none>
	N of Rows in Working Data File	156
Missing Value Handling	Definition of Missing	User-defined missing values are treated as missing.
	Cases Used	Statistics are based on all cases with valid data.
Syntax		FREQUENCIES VARIABLES=ISOLATED /NTILES=4 /STATISTICS=STDDEV MEAN /ORDER=ANALYSIS.
Resources	Processor Time	00:00:00.00
	Elapsed Time	00:00:00.01

Statistics

ISOLATED

N	Valid	142
	Missing	14
Mean		.92
Std. Deviation		.279
Percentiles	25	1.00
	50	1.00
	75	1.00

ISOLATED

		Frequency	Percent	Valid Percent	Cumulative Percent
Valid	CABG+valve	12	7.7	8.5	8.5
	CABG	130	83.3	91.5	100.0
	Total	142	91.0	100.0	
Missing	9	14	9.0		
Total		156	100.0		

```
CROSSTABS
  /TABLES=ISOLATED BY AF
  /FORMAT=AVALUE TABLES
  /STATISTICS=CHISQ
  /CELLS=COUNT ROW COLUMN
  /COUNT ROUND CELL.
```

Crosstabs

Notes

Output Created		01-MAR-2021 14:45:55
Comments		
Input	Data	C:\Users\user\Downloads\ImAnisah Statistic & SPSS\TOCO T3\Data toco.sav
	Active Dataset	DataSet1
	Filter	<none>
	Weight	<none>
	Split File	<none>
	N of Rows in Working Data File	156
Missing Value Handling	Definition of Missing	User-defined missing values are treated as missing.
	Cases Used	Statistics for each table are based on all the cases with valid data in the specified range(s) for all variables in each table.
Syntax		CROSSTABS /TABLES=ISOLATED BY AF /FORMAT=AVALUE TABLES /STATISTICS=CHISQ /CELLS=COUNT ROW COLUMN /COUNT ROUND CELL.
Resources	Processor Time	00:00:00.03
	Elapsed Time	00:00:00.04
	Dimensions Requested	2
	Cells Available	524245

Case Processing Summary

	Cases					
	Valid		Missing		Total	
	N	Percent	N	Percent	N	Percent
ISOLATED * AF	142	91.0%	14	9.0%	156	100.0%

ISOLATED * AF Crosstabulation

			AF		Total
			No	Yes	
ISOLATED	CABG+valve	Count	8	4	12
		% within ISOLATED	66.7%	33.3%	100.0%
		% within AF	7.7%	10.5%	8.5%
	CABG	Count	96	34	130
		% within ISOLATED	73.8%	26.2%	100.0%
		% within AF	92.3%	89.5%	91.5%
Total		Count	104	38	142
		% within ISOLATED	73.2%	26.8%	100.0%
		% within AF	100.0%	100.0%	100.0%

Chi-Square Tests

	Value	df	Asymptotic Significance (2-sided)	Exact Sig. (2-sided)	Exact Sig. (1-sided)
Pearson Chi-Square	.289[a]	1	.591		
Continuity Correction[b]	.039	1	.844		
Likelihood Ratio	.277	1	.599		
Fisher's Exact Test				.734	.405
Linear-by-Linear Association	.287	1	.592		
N of Valid Cases	142				

a. 1 cells (25.0%) have expected count less than 5. The minimum expected count is 3.21.

b. Computed only for a 2x2 table

```
T-TEST GROUPS=AF(0 1)
 /MISSING=ANALYSIS
 /VARIABLES=XCLAMPTIME BYPASSTIME
 /CRITERIA=CI(.95).
```

T-Test

Notes

Output Created		01-MAR-2021 14:46:22
Comments		
Input	Data	C:\Users\user\Downloads\ImAnisah Statistic & SPSS\TOCO T3\Data toco.sav
	Active Dataset	DataSet1
	Filter	<none>
	Weight	<none>
	Split File	<none>
	N of Rows in Working Data File	156
Missing Value Handling	Definition of Missing	User defined missing values are treated as missing.
	Cases Used	Statistics for each analysis are based on the cases with no missing or out-of-range data for any variable in the analysis.
Syntax		T-TEST GROUPS=AF(0 1)<br>/MISSING=ANALYSIS<br>/VARIABLES=XCLAMPTIME BYPASSTIME<br>/CRITERIA=CI(.95).
Resources	Processor Time	00:00:00.02
	Elapsed Time	00:00:00.07

Group Statistics

	AF	N	Mean	Std. Deviation	Std. Error Mean
XCLAMPTIME	No	38	70.95	36.027	5.844
	Yes	21	73.19	31.020	6.769
BYPASSTIME	No	20	96.75	57.230	12.797
	Yes	21	94.33	38.506	8.403

Independent Samples Test

		Levene's Test for Equality of Variances		t-test for Equality of Means						
									95% Confidence Interval of the Difference	
		F	Sig.	t	df	Sig. (2-tailed)	Mean Difference	Std. Error Difference	Lower	Upper
XCLAMPTIME	Equal variances assumed	.011	.916	-.240	57	.811	-2.243	9.341	-20.948	16.462
	Equal variances not assumed			-.251	46.856	.803	-2.243	8.943	-20.236	15.749
BYPASSTIME	Equal variances assumed	.856	.360	.159	39	.874	2.417	15.165	-28.258	33.092
	Equal variances not assumed			.158	33.075	.876	2.417	15.309	-28.727	33.561

```
EXAMINE VARIABLES=BYPASSTIME XCLAMPTIME BY AF
  /PLOT BOXPLOT STEMLEAF NPPLOT
  /COMPARE GROUPS
```

```
/STATISTICS DESCRIPTIVES
/CINTERVAL 95
/MISSING LISTWISE
/NOTOTAL.
```

Explore

Notes

Output Created		01-MAR-2021 14:47:14
Comments		
Input	Data	C:\Users\user\Downloads\ImAnisah Statistic & SPSS\TOCO T3\Data toco.sav
	Active Dataset	DataSet1
	Filter	<none>
	Weight	<none>
	Split File	<none>
	N of Rows in Working Data File	156
Missing Value Handling	Definition of Missing	User-defined missing values for dependent variables are treated as missing.
	Cases Used	Statistics are based on cases with no missing values for any dependent variable or factor used.
Syntax		EXAMINE VARIABLES=BYPASSTIME XCLAMPTIME BY AF<br>/PLOT BOXPLOT STEMLEAF NPPLOT<br>/COMPARE GROUPS<br>/STATISTICS DESCRIPTIVES<br>/CINTERVAL 95<br>/MISSING LISTWISE<br>/NOTOTAL.
Resources	Processor Time	00:00:03.64
	Elapsed Time	00:00:03.70

AF

Case Processing Summary

		Cases					
		Valid		Missing		Total	
	AF	N	Percent	N	Percent	N	Percent
BYPASSTIME	No	16	13.6%	102	86.4%	118	100.0%
	Yes	18	47.4%	20	52.6%	38	100.0%
XCLAMPTIME	No	16	13.6%	102	86.4%	118	100.0%
	Yes	18	47.4%	20	52.6%	38	100.0%

Descriptives

	AF			Statistic	Std. Error
BYPASSTIME	No	Mean		98.56	15.664
		95% Confidence Interval for Mean	Lower Bound	65.18	
			Upper Bound	131.95	
		5% Trimmed Mean		89.85	
		Median		78.50	
		Variance		3925.729	
		Std. Deviation		62.656	
		Minimum		50	
		Maximum		304	
		Range		254	
		Interquartile Range		65	
		Skewness		2.572	.564
		Kurtosis		7.980	1.091
	Yes	Mean		95.56	9.615
		95% Confidence Interval for Mean	Lower Bound	75.27	
			Upper Bound	115.84	
		5% Trimmed Mean		91.40	
		Median		90.50	
		Variance		1664.144	
		Std. Deviation		40.794	
		Minimum		50	
		Maximum		216	
		Range		166	
		Interquartile Range		52	
		Skewness		1.483	.536
		Kurtosis		3.349	1.038
XCLAMPTIME	No	Mean		73.56	12.478
		95% Confidence Interval for Mean	Lower Bound	46.97	
			Upper Bound	100.16	
		5% Trimmed Mean		66.12	
		Median		56.00	
		Variance		2491.063	
		Std. Deviation		49.911	
		Minimum		37	
		Maximum		244	
		Range		207	
		Interquartile Range		43	
		Skewness		2.932	.564
		Kurtosis		9.959	1.091
	Yes	Mean		70.33	7.647
		95% Confidence Interval for Mean	Lower Bound	54.20	
			Upper Bound	86.47	
		5% Trimmed Mean		67.59	

Descriptives

AF		Statistic	Std. Error
	Median	64.50	
	Variance	1052.706	
	Std. Deviation	32.445	
	Minimum	22	
	Maximum	168	
	Range	146	
	Interquartile Range	37	
	Skewness	1.522	.536
	Kurtosis	3.994	1.038

Tests of Normality

		Kolmogorov-Smirnov[a]			Shapiro-Wilk		
	AF	Statistic	df	Sig.	Statistic	df	Sig.
BYPASSTIME	No	.219	16	.039	.705	16	.000
	Yes	.132	18	.200*	.872	18	.020
XCLAMPTIME	No	.246	16	.011	.650	16	.000
	Yes	.161	18	.200*	.880	18	.026

*. This is a lower bound of the true significance.

a. Lilliefors Significance Correction

BYPASSTIME

Stem-and-Leaf Plots

```
BYPASSTIME Stem-and-Leaf Plot for
AF= No

 Frequency    Stem &  Leaf

     2.00        5 .  00
     2.00        5 .  79
     1.00        6 .  1
     2.00        6 .  57
     1.00        7 .  1
      .00        7 .
      .00        8 .
     2.00        8 .  66
      .00        9 .
      .00        9 .
      .00       10 .
      .00       10 .
     2.00       11 .  00
```

```
      .00       11 .
      .00       12 .
     1.00       12 .  9
     1.00       13 .  0
      .00       13 .
     1.00       14 .  2
     1.00 Extremes    (>=304)

 Stem width:        10
 Each leaf:        1 case(s)
```

```
BYPASSTIME Stem-and-Leaf Plot for
AF= Yes

 Frequency    Stem &  Leaf

     4.00        5 .  0259
     2.00        6 .  28
     1.00        7 .  5
     2.00        8 .  07
     1.00        9 .  4
     3.00       10 .  889
     2.00       11 .  28
     1.00       12 .  8
     1.00       13 .  9
     1.00 Extremes    (>=216)

 Stem width:        10
 Each leaf:        1 case(s)
```

Normal Q-Q Plots

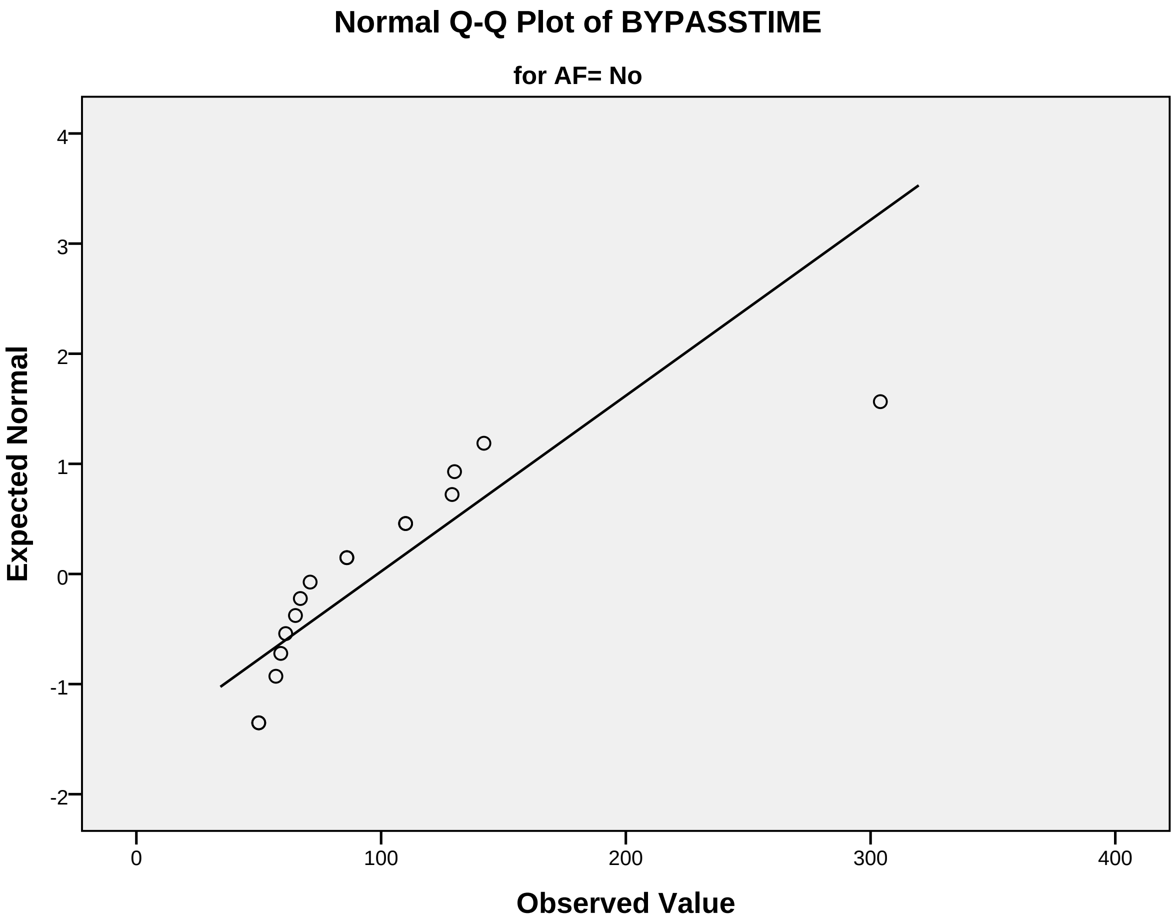
Normal Q-Q Plot of BYPASSTIME
for AF= No
Expected Normal
Observed Value
4
3
2
1
0
-1
-2
0
100
200
300
400

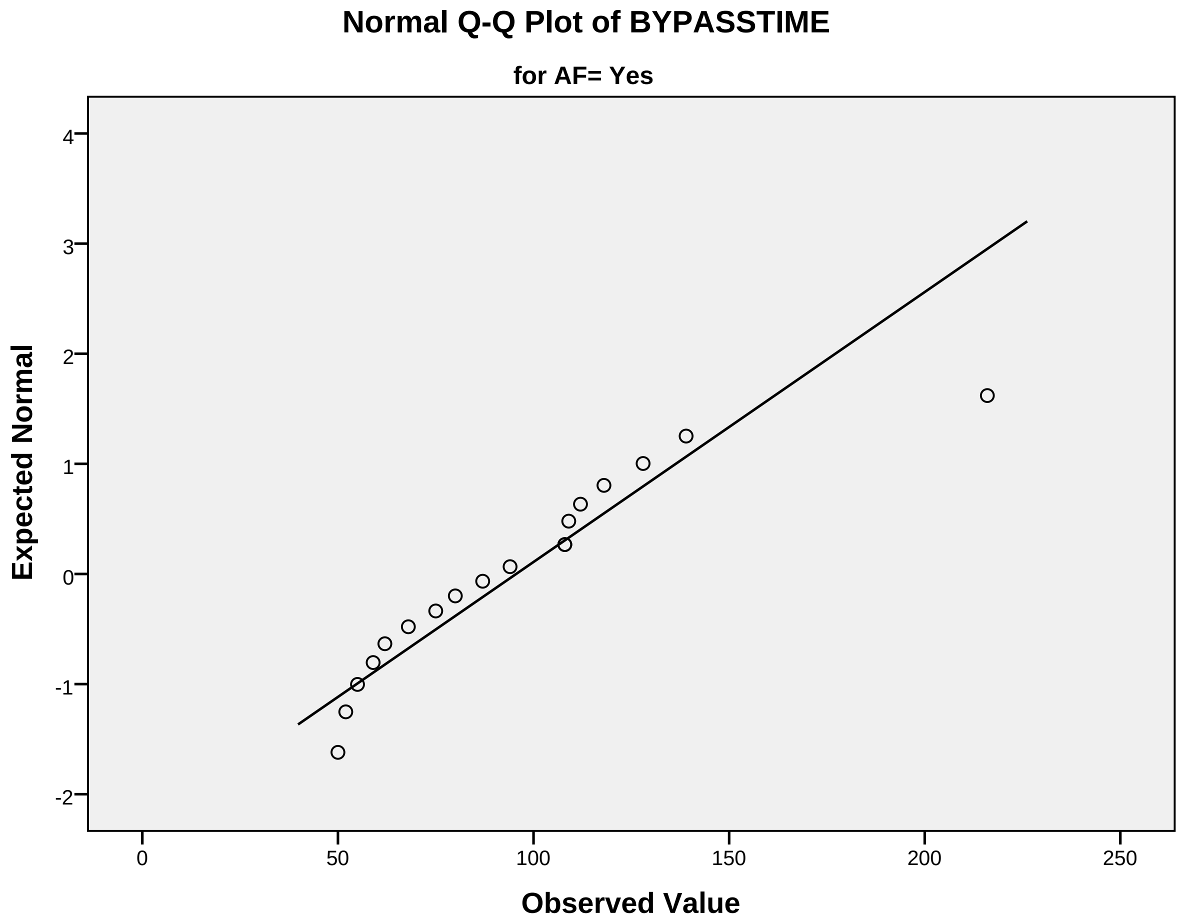

Detrended Normal Q-Q Plots

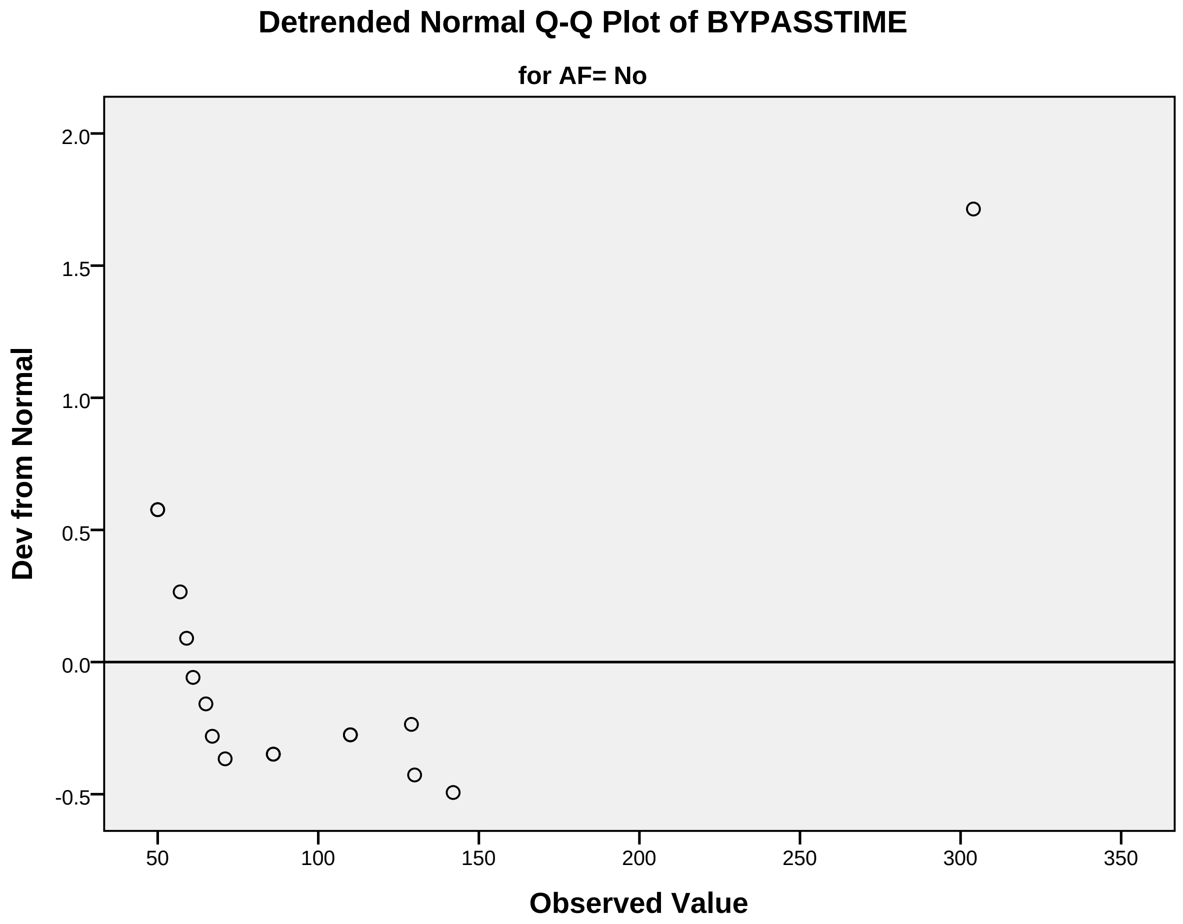
Detrended Normal Q-Q Plot of BYPASSTIME
for AF= No
Dev from Normal
2.0
1.5
1.0
0.5
0.0
-0.5
50
100
150
200
250
300
350
Observed Value

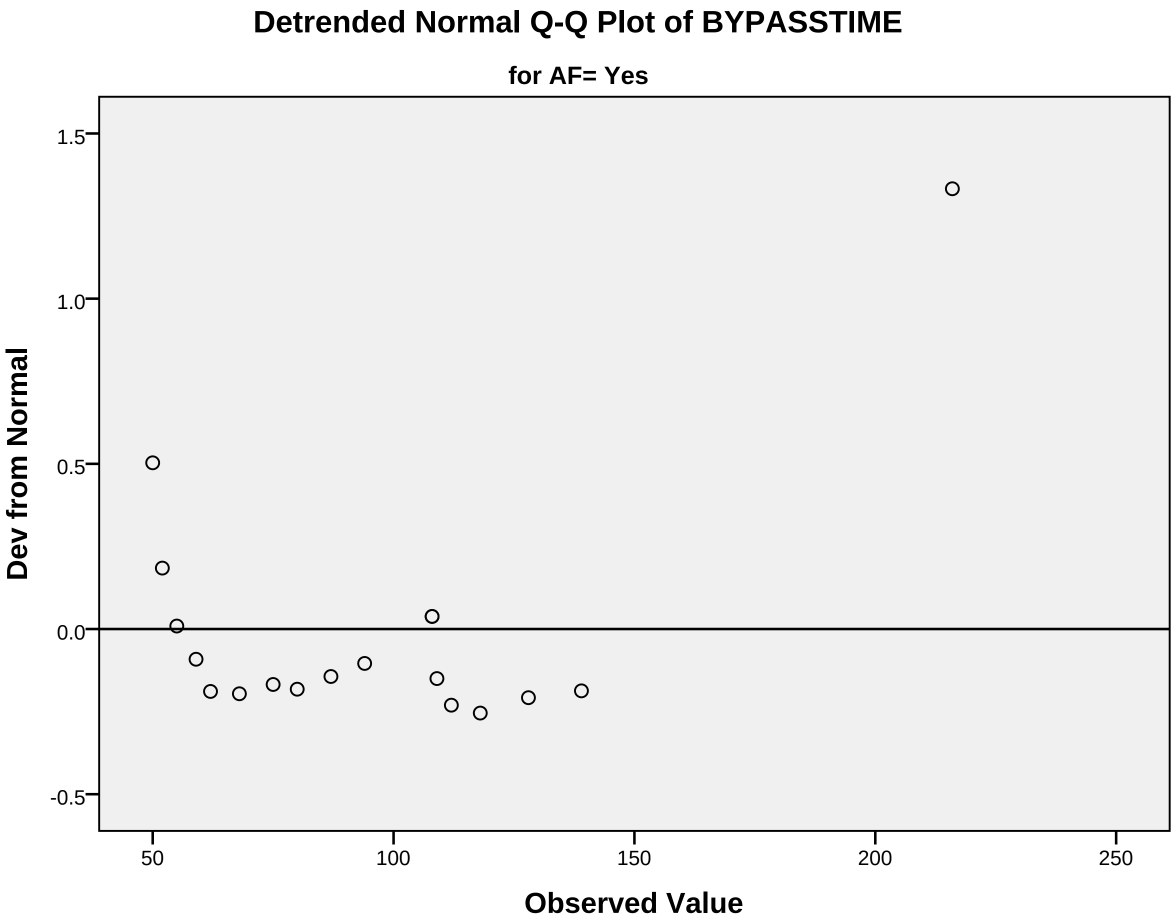
Detrended Normal Q-Q Plot of BYPASSTIME
for AF= Yes
Dev from Normal
1.5
1.0
0.5
0.0
-0.5
50
100
150
200
250
Observed Value

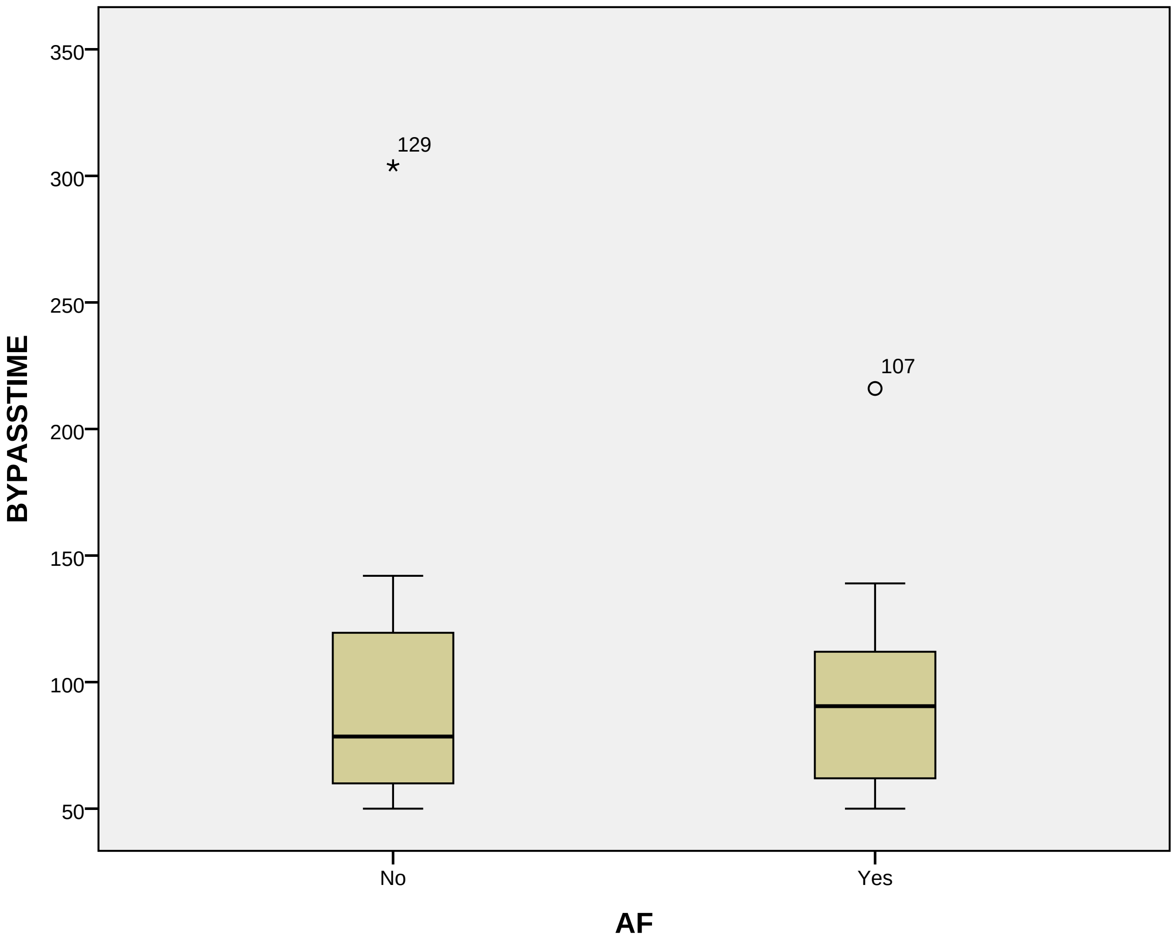

XCLAMPTIME

Stem-and-Leaf Plots

```
XCLAMPTIME Stem-and-Leaf Plot for
AF= No

 Frequency    Stem &  Leaf

     2.00        3 .  78
     2.00        4 .  12
      .00        4 .
     3.00        5 .  014
     2.00        5 .  57
      .00        6 .
     1.00        6 .  5
      .00        7 .
     1.00        7 .  9
     1.00        8 .  1
     1.00        8 .  9
     1.00        9 .  0
```

```
      .00        9 .
     1.00       10 .  4
     1.00 Extremes    (>=244)

 Stem width:        10
 Each leaf:        1 case(s)
```

```
XCLAMPTIME Stem-and-Leaf Plot for
AF= Yes

 Frequency    Stem &  Leaf

     1.00        2 .  2
      .00        3 .
     4.00        4 .  0267
     2.00        5 .  12
     3.00        6 .  345
     1.00        7 .  7
     4.00        8 .  3335
     1.00        9 .  0
     1.00       10 .  5
     1.00 Extremes    (>=168)

 Stem width:        10
 Each leaf:        1 case(s)
```

Normal Q-Q Plots

Normal Q-Q Plot of XCLAMPTIME

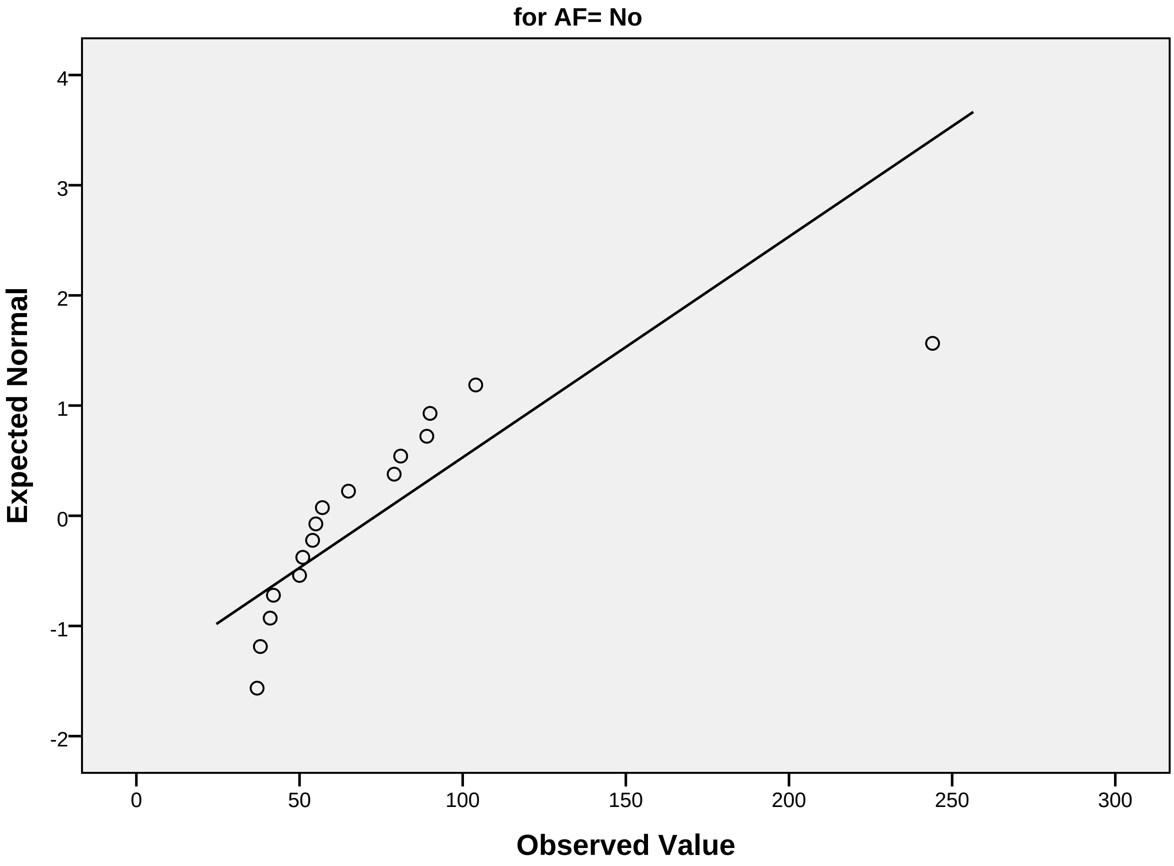

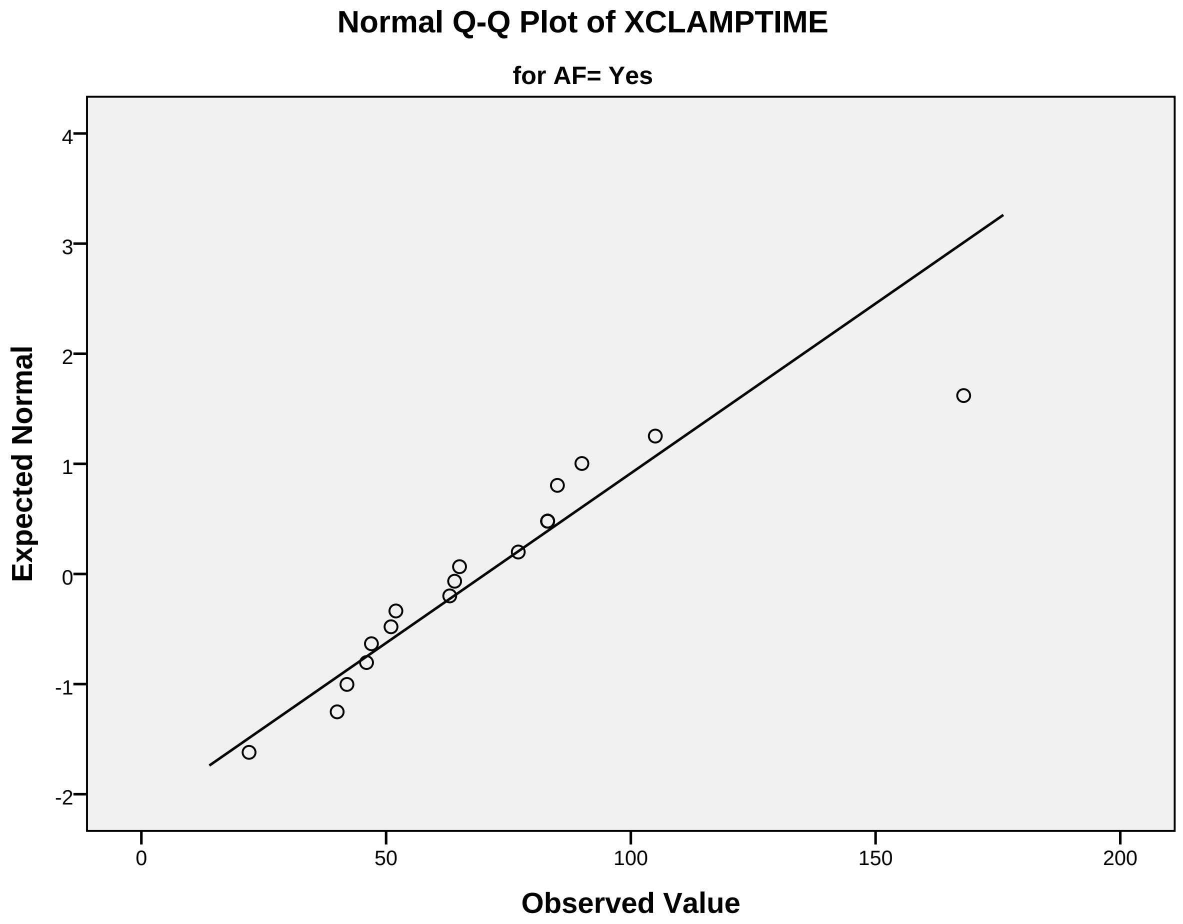

Detrended Normal Q-Q Plots

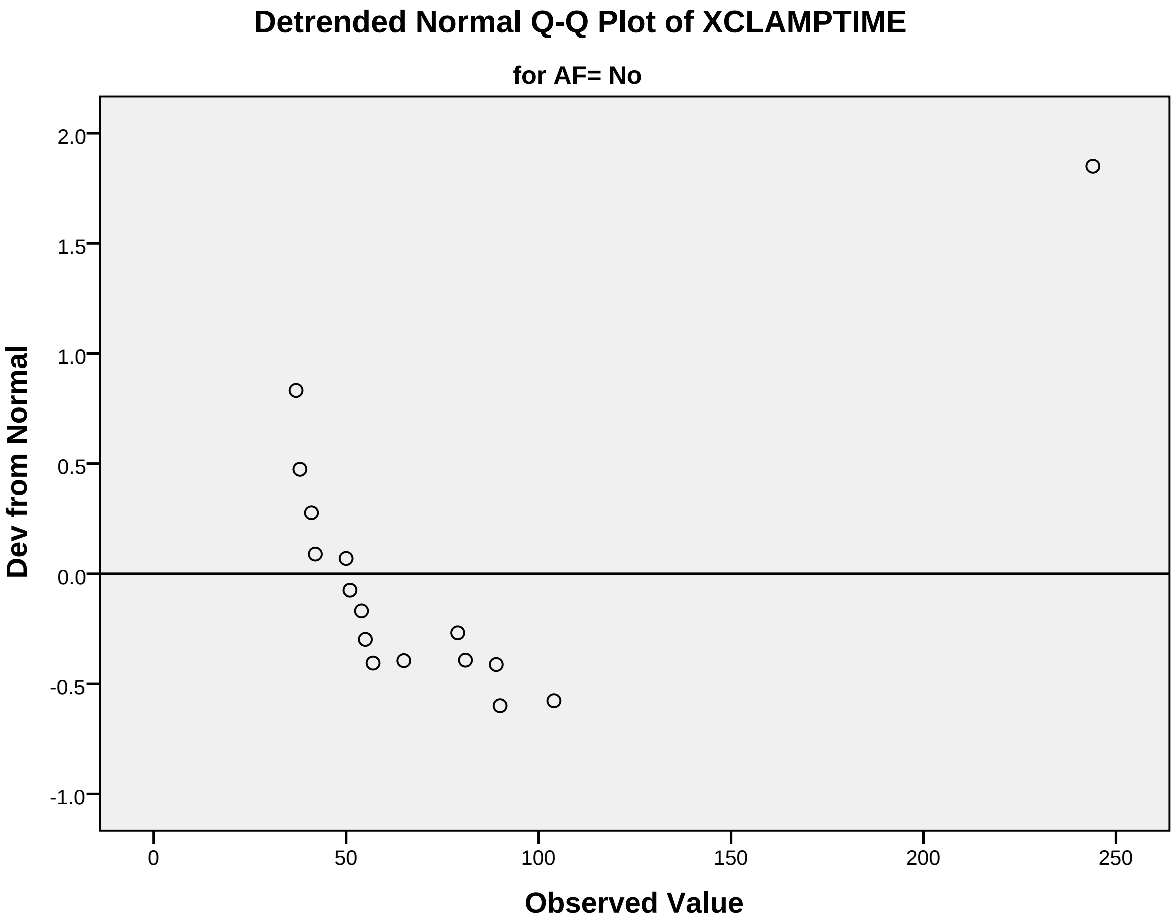
Detrended Normal Q-Q Plot of XCLAMPTIME
for AF= No
Dev from Normal
2.0
1.5
1.0
0.5
0.0
-0.5
-1.0
0
50
100
150
200
250
Observed Value

Detrended Normal Q-Q Plot of XCLAMPTIME

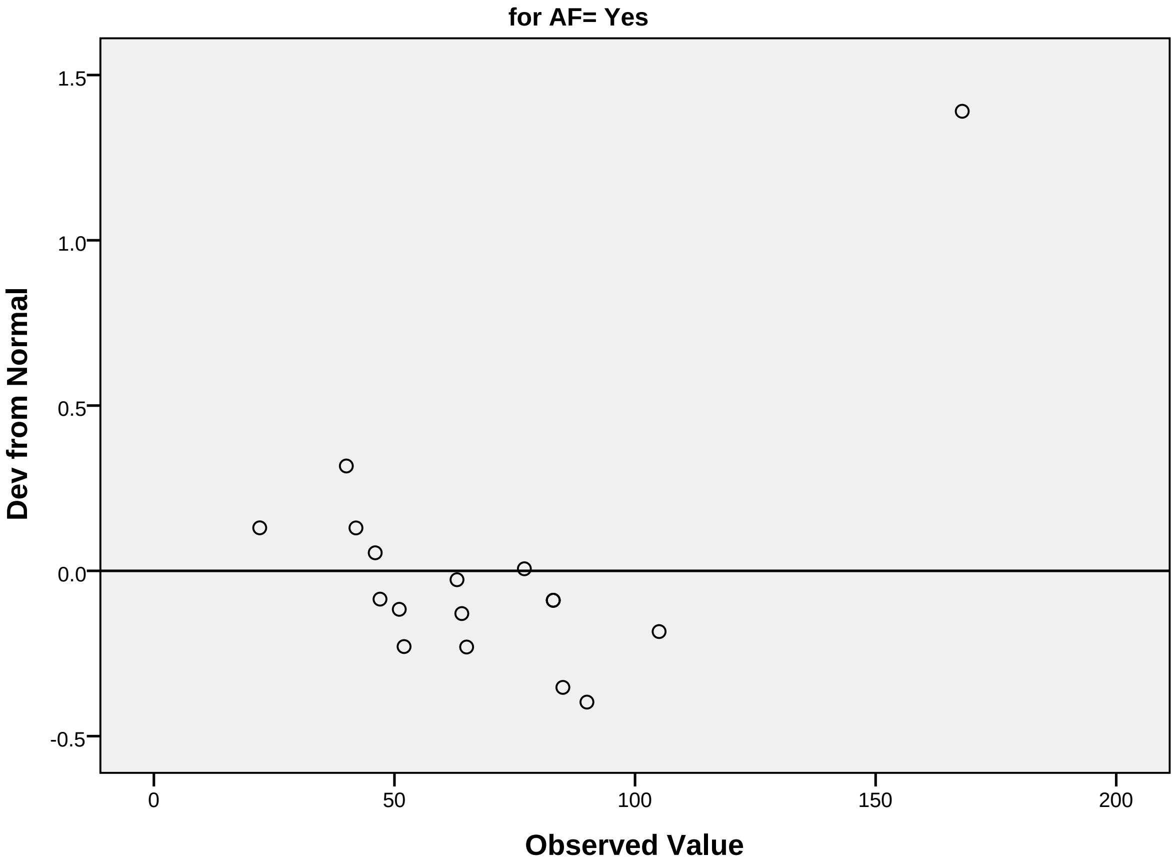

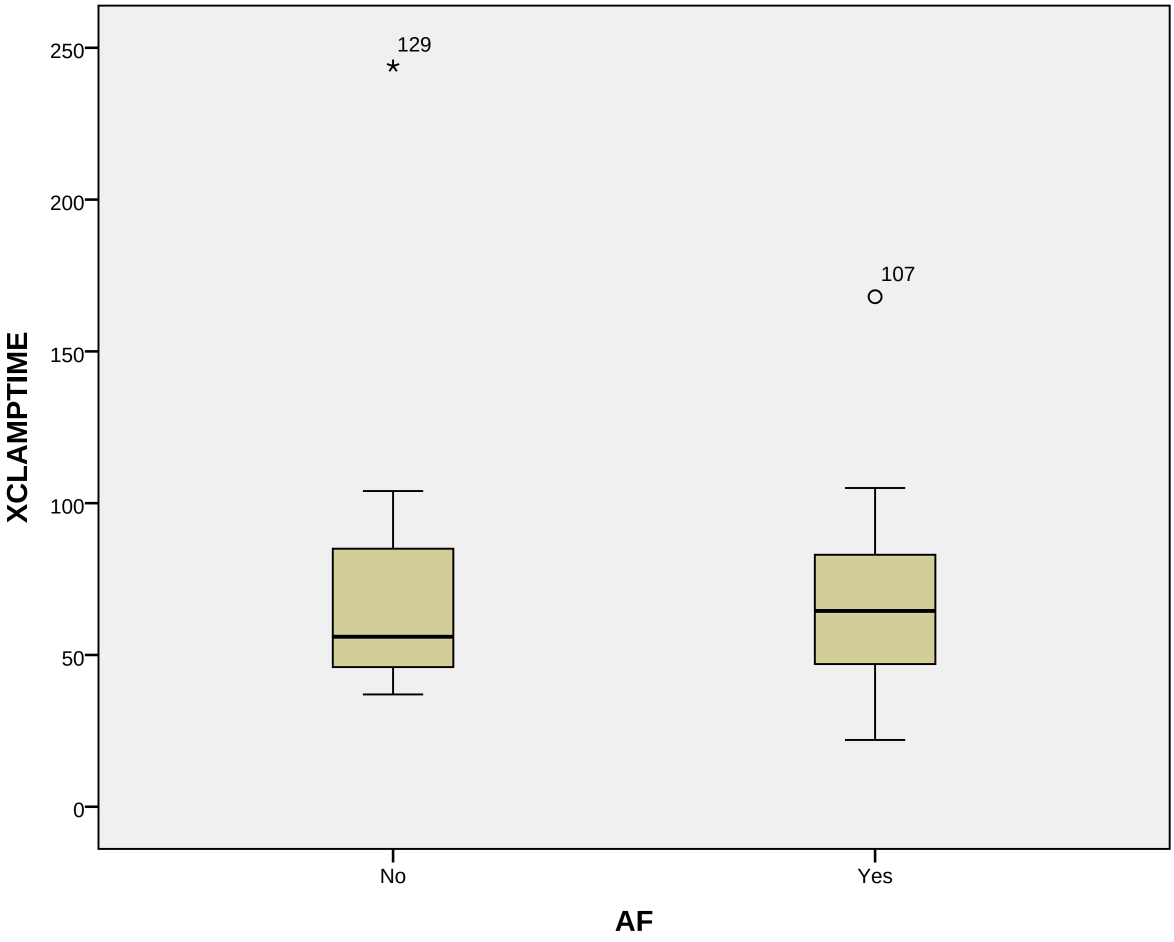


```
 CROSSTABS
  /TABLES=Stroke Sternalinfect Respiratory Renalfailure Endocrine PleuralEf
fusion Tamponade Fever
    Hyperkal others Death BY AF
  /FORMAT=AVALUE TABLES
  /STATISTICS=CHISQ
  /CELLS=COUNT ROW COLUMN
  /COUNT ROUND CELL.
```

Crosstabs

Notes

Output Created		01-MAR-2021 14:48:58
Comments		
Input	Data	C:\Users\user\Downloads\ImAnisah Statistic & SPSS\TOCO T3\Data toco.sav
	Active Dataset	DataSet1
	Filter	<none>
	Weight	<none>
	Split File	<none>
	N of Rows in Working Data File	156
Missing Value Handling	Definition of Missing	User-defined missing values are treated as missing.
	Cases Used	Statistics for each table are based on all the cases with valid data in the specified range(s) for all variables in each table.
Syntax		CROSSTABS /TABLES=Stroke Sternalinfect Respiratory Renalfailure Endocrine PleuralEffusion Tamponade Fever Hyperkal others Death BY AF /FORMAT=AVALUE TABLES /STATISTICS=CHISQ /CELLS=COUNT ROW COLUMN /COUNT ROUND CELL.
Resources	Processor Time	00:00:00.08
	Elapsed Time	00:00:00.12
	Dimensions Requested	2
	Cells Available	524245

Case Processing Summary

	Cases					
	Valid		Missing		Total	
	N	Percent	N	Percent	N	Percent
Stroke * AF	101	64.7%	55	35.3%	156	100.0%
Sternalinfect * AF	101	64.7%	55	35.3%	156	100.0%
Respiratory * AF	101	64.7%	55	35.3%	156	100.0%
Renalfailure * AF	101	64.7%	55	35.3%	156	100.0%
Endocrine * AF	101	64.7%	55	35.3%	156	100.0%
PleuralEffusion * AF	101	64.7%	55	35.3%	156	100.0%
Tamponade * AF	101	64.7%	55	35.3%	156	100.0%
Fever * AF	101	64.7%	55	35.3%	156	100.0%
Hyperkal * AF	101	64.7%	55	35.3%	156	100.0%
others * AF	101	64.7%	55	35.3%	156	100.0%
Death * AF	101	64.7%	55	35.3%	156	100.0%

Stroke * AF

Crosstab

			AF		
			No	Yes	Total
Stroke	No	Count	69	28	97
		% within Stroke	71.1%	28.9%	100.0%
		% within AF	97.2%	93.3%	96.0%
	Yes	Count	2	2	4
		% within Stroke	50.0%	50.0%	100.0%
		% within AF	2.8%	6.7%	4.0%
Total		Count	71	30	101
		% within Stroke	70.3%	29.7%	100.0%
		% within AF	100.0%	100.0%	100.0%

Chi-Square Tests

	Value	df	Asymptotic Significance (2-sided)	Exact Sig. (2-sided)	Exact Sig. (1-sided)
Pearson Chi-Square	.822[a]	1	.365		
Continuity Correction[b]	.121	1	.728		
Likelihood Ratio	.753	1	.386		
Fisher's Exact Test				.580	.342
Linear-by-Linear Association	.814	1	.367		
N of Valid Cases	101				

a. 2 cells (50.0%) have expected count less than 5. The minimum expected count is 1.19.

b. Computed only for a 2x2 table

Sternalinfect * AF

Crosstab

			AF		
			No	Yes	Total
Sternalinfect	No	Count	68	30	98
		% within Sternalinfect	69.4%	30.6%	100.0%
		% within AF	95.8%	100.0%	97.0%
	Yes	Count	3	0	3
		% within Sternalinfect	100.0%	0.0%	100.0%
		% within AF	4.2%	0.0%	3.0%
Total		Count	71	30	101
		% within Sternalinfect	70.3%	29.7%	100.0%
		% within AF	100.0%	100.0%	100.0%

Chi-Square Tests

	Value	df	Asymptotic Significance (2-sided)	Exact Sig. (2-sided)	Exact Sig. (1-sided)
Pearson Chi-Square	1.306[a]	1	.253		
Continuity Correction[b]	.252	1	.616		
Likelihood Ratio	2.153	1	.142		
Fisher's Exact Test				.553	.343
Linear-by-Linear Association	1.293	1	.255		
N of Valid Cases	101				

a. 2 cells (50.0%) have expected count less than 5. The minimum expected count is .89.

b. Computed only for a 2x2 table

Respiratory * AF

Crosstab

			AF		Total
			No	Yes	
Respiratory	No	Count	68	27	95
		% within Respiratory	71.6%	28.4%	100.0%
		% within AF	95.8%	90.0%	94.1%
	Yes	Count	3	3	6
		% within Respiratory	50.0%	50.0%	100.0%
		% within AF	4.2%	10.0%	5.9%
Total		Count	71	30	101
		% within Respiratory	70.3%	29.7%	100.0%
		% within AF	100.0%	100.0%	100.0%

Chi-Square Tests

	Value	df	Asymptotic Significance (2-sided)	Exact Sig. (2-sided)	Exact Sig. (1-sided)
Pearson Chi-Square	1.259[a]	1	.262		
Continuity Correction[b]	.437	1	.508		
Likelihood Ratio	1.156	1	.282		
Fisher's Exact Test				.358	.245
Linear-by-Linear Association	1.246	1	.264		
N of Valid Cases	101				

a. 2 cells (50.0%) have expected count less than 5. The minimum expected count is 1.78.

b. Computed only for a 2x2 table

Renalfailure * AF

Crosstab

			AF		Total
			No	Yes	
Renalfailure	No	Count	67	29	96
		% within Renalfailure	69.8%	30.2%	100.0%
		% within AF	94.4%	96.7%	95.0%
	Yes	Count	4	1	5
		% within Renalfailure	80.0%	20.0%	100.0%
		% within AF	5.6%	3.3%	5.0%
Total		Count	71	30	101
		% within Renalfailure	70.3%	29.7%	100.0%
		% within AF	100.0%	100.0%	100.0%

Chi-Square Tests

	Value	df	Asymptotic Significance (2-sided)	Exact Sig. (2-sided)	Exact Sig. (1-sided)
Pearson Chi-Square	.237[a]	1	.626		
Continuity Correction[b]	.000	1	1.000		
Likelihood Ratio	.255	1	.614		
Fisher's Exact Test				1.000	.532
Linear-by-Linear Association	.235	1	.628		
N of Valid Cases	101				

a. 2 cells (50.0%) have expected count less than 5. The minimum expected count is 1.49.

b. Computed only for a 2x2 table

Endocrine * AF

Crosstab

			AF		Total
			No	Yes	
Endocrine	No	Count	70	30	100
		% within Endocrine	70.0%	30.0%	100.0%
		% within AF	98.6%	100.0%	99.0%
	Yes	Count	1	0	1
		% within Endocrine	100.0%	0.0%	100.0%
		% within AF	1.4%	0.0%	1.0%
Total		Count	71	30	101
		% within Endocrine	70.3%	29.7%	100.0%
		% within AF	100.0%	100.0%	100.0%

Chi-Square Tests

	Value	df	Asymptotic Significance (2-sided)	Exact Sig. (2-sided)	Exact Sig. (1-sided)
Pearson Chi-Square	.427[a]	1	.514		
Continuity Correction[b]	.000	1	1.000		
Likelihood Ratio	.709	1	.400		
Fisher's Exact Test				1.000	.703
Linear-by-Linear Association	.423	1	.516		
N of Valid Cases	101				

a. 2 cells (50.0%) have expected count less than 5. The minimum expected count is .30.

b. Computed only for a 2x2 table

PleuralEffusion * AF

Crosstab

			AF		Total
			No	Yes	
PleuralEffusion	No	Count	68	28	96
		% within PleuralEffusion	70.8%	29.2%	100.0%
		% within AF	95.8%	93.3%	95.0%
	Yes	Count	3	2	5
		% within PleuralEffusion	60.0%	40.0%	100.0%
		% within AF	4.2%	6.7%	5.0%
Total		Count	71	30	101
		% within PleuralEffusion	70.3%	29.7%	100.0%
		% within AF	100.0%	100.0%	100.0%

Chi-Square Tests

	Value	df	Asymptotic Significance (2-sided)	Exact Sig. (2-sided)	Exact Sig. (1-sided)
Pearson Chi-Square	.267[a]	1	.605		
Continuity Correction[b]	.000	1	.988		
Likelihood Ratio	.253	1	.615		
Fisher's Exact Test				.632	.468
Linear-by-Linear Association	.264	1	.607		
N of Valid Cases	101				

a. 2 cells (50.0%) have expected count less than 5. The minimum expected count is 1.49.

b. Computed only for a 2x2 table

Tamponade * AF

Crosstab

			AF		Total
			No	Yes	
Tamponade	No	Count	58	29	87
		% within Tamponade	66.7%	33.3%	100.0%
		% within AF	81.7%	96.7%	86.1%
	Yes	Count	13	1	14
		% within Tamponade	92.9%	7.1%	100.0%
		% within AF	18.3%	3.3%	13.9%
Total		Count	71	30	101
		% within Tamponade	70.3%	29.7%	100.0%
		% within AF	100.0%	100.0%	100.0%

Chi-Square Tests

	Value	df	Asymptotic Significance (2-sided)	Exact Sig. (2-sided)	Exact Sig. (1-sided)
Pearson Chi-Square	3.962[a]	1	.047		
Continuity Correction[b]	2.807	1	.094		
Likelihood Ratio	4.924	1	.026		
Fisher's Exact Test				.059	.039
Linear-by-Linear Association	3.922	1	.048		
N of Valid Cases	101				

a. 1 cells (25.0%) have expected count less than 5. The minimum expected count is 4.16.

b. Computed only for a 2x2 table

Fever * AF

Crosstab

			AF		Total
			No	Yes	
Fever	No	Count	67	28	95
		% within Fever	70.5%	29.5%	100.0%
		% within AF	94.4%	93.3%	94.1%
	Yes	Count	4	2	6
		% within Fever	66.7%	33.3%	100.0%
		% within AF	5.6%	6.7%	5.9%
Total		Count	71	30	101
		% within Fever	70.3%	29.7%	100.0%
		% within AF	100.0%	100.0%	100.0%

Chi-Square Tests

	Value	df	Asymptotic Significance (2-sided)	Exact Sig. (2-sided)	Exact Sig. (1-sided)
Pearson Chi-Square	.040[a]	1	.841		
Continuity Correction[b]	.000	1	1.000		
Likelihood Ratio	.039	1	.843		
Fisher's Exact Test				1.000	.579
Linear-by-Linear Association	.040	1	.842		
N of Valid Cases	101				

a. 2 cells (50.0%) have expected count less than 5. The minimum expected count is 1.78.

b. Computed only for a 2x2 table

Hyperkal * AF

Crosstab

			AF		Total
			No	Yes	
Hyperkal	No	Count	68	29	97
		% within Hyperkal	70.1%	29.9%	100.0%
		% within AF	95.8%	96.7%	96.0%
	Yes	Count	3	1	4
		% within Hyperkal	75.0%	25.0%	100.0%
		% within AF	4.2%	3.3%	4.0%
Total		Count	71	30	101
		% within Hyperkal	70.3%	29.7%	100.0%
		% within AF	100.0%	100.0%	100.0%

Chi-Square Tests

	Value	df	Asymptotic Significance (2-sided)	Exact Sig. (2-sided)	Exact Sig. (1-sided)
Pearson Chi-Square	.044[a]	1	.834		
Continuity Correction[b]	.000	1	1.000		
Likelihood Ratio	.046	1	.831		
Fisher's Exact Test				1.000	.658
Linear-by-Linear Association	.044	1	.834		
N of Valid Cases	101				

a. 2 cells (50.0%) have expected count less than 5. The minimum expected count is 1.19.

b. Computed only for a 2x2 table

others * AF

Crosstab

			AF		Total
			No	Yes	
others	No	Count	70	28	98
		% within others	71.4%	28.6%	100.0%
		% within AF	98.6%	93.3%	97.0%
	Yes	Count	1	2	3
		% within others	33.3%	66.7%	100.0%
		% within AF	1.4%	6.7%	3.0%
Total		Count	71	30	101
		% within others	70.3%	29.7%	100.0%
		% within AF	100.0%	100.0%	100.0%

Chi-Square Tests

	Value	df	Asymptotic Significance (2-sided)	Exact Sig. (2-sided)	Exact Sig. (1-sided)
Pearson Chi-Square	2.023[a]	1	.155		
Continuity Correction[b]	.610	1	.435		
Likelihood Ratio	1.802	1	.179		
Fisher's Exact Test				.210	.210
Linear-by-Linear Association	2.003	1	.157		
N of Valid Cases	101				

a. 2 cells (50.0%) have expected count less than 5. The minimum expected count is .89.

b. Computed only for a 2x2 table

Death * AF

Crosstab

			AF		Total
			No	Yes	
Death	No	Count	69	24	93
		% within Death	74.2%	25.8%	100.0%
		% within AF	97.2%	80.0%	92.1%
	Yes	Count	2	6	8
		% within Death	25.0%	75.0%	100.0%
		% within AF	2.8%	20.0%	7.9%
Total		Count	71	30	101
		% within Death	70.3%	29.7%	100.0%
		% within AF	100.0%	100.0%	100.0%

Chi-Square Tests

	Value	df	Asymptotic Significance (2-sided)	Exact Sig. (2-sided)	Exact Sig. (1-sided)
Pearson Chi-Square	8.538[a]	1	.003		
Continuity Correction[b]	6.344	1	.012		
Likelihood Ratio	7.674	1	.006		
Fisher's Exact Test				.008	.008
Linear-by-Linear Association	8.453	1	.004		
N of Valid Cases	101				

a. 1 cells (25.0%) have expected count less than 5. The minimum expected count is 2.38.

b. Computed only for a 2x2 table

```
EXAMINE VARIABLES=VENT CICUstay HDUstay HospStay BY AF
  /PLOT BOXPLOT STEMLEAF NPPLOT
  /COMPARE GROUPS
  /STATISTICS DESCRIPTIVES
  /CINTERVAL 95
  /MISSING LISTWISE
  /NOTOTAL.
```

Explore

Notes

Output Created		01-MAR-2021 14:55:19
Comments		
Input	Data	C:\Users\user\Downloads\ImAnisah Statistic & SPSS\TOCO T3\Data toco.sav
	Active Dataset	DataSet1
	Filter	<none>
	Weight	<none>
	Split File	<none>
	N of Rows in Working Data File	156
Missing Value Handling	Definition of Missing	User-defined missing values for dependent variables are treated as missing.
	Cases Used	Statistics are based on cases with no missing values for any dependent variable or factor used.
Syntax		EXAMINE VARIABLES=VENT CICUstay HDUstay HospStay BY AF<br>/PLOT BOXPLOT STEMLEAF NPPLOT<br>/COMPARE GROUPS<br>/STATISTICS DESCRIPTIVES<br>/CINTERVAL 95<br>/MISSING LISTWISE<br>/NOTOTAL.
Resources	Processor Time	00:00:08.58
	Elapsed Time	00:00:07.88

AF

Case Processing Summary

		Cases					
		Valid		Missing		Total	
	AF	N	Percent	N	Percent	N	Percent
Total hours intubated	No	32	27.1%	86	72.9%	118	100.0%
	Yes	18	47.4%	20	52.6%	38	100.0%
CICUstay	No	32	27.1%	86	72.9%	118	100.0%
	Yes	18	47.4%	20	52.6%	38	100.0%
HDUstay	No	32	27.1%	86	72.9%	118	100.0%
	Yes	18	47.4%	20	52.6%	38	100.0%
HospStay	No	32	27.1%	86	72.9%	118	100.0%
	Yes	18	47.4%	20	52.6%	38	100.0%

Descriptives

	AF			Statistic	Std. Error
Total hours intubated	No	Mean		1413.56	170.067
		95% Confidence Interval for Mean	Lower Bound	1066.71	
			Upper Bound	1760.42	
		5% Trimmed Mean		1257.64	
		Median		1157.50	
		Variance		925533.609	
		Std. Deviation		962.047	
		Minimum		860	
		Maximum		6375	
		Range		5515	
		Interquartile Range		391	
		Skewness		4.707	.414
		Kurtosis		24.432	.809
	Yes	Mean		1902.89	403.849
		95% Confidence Interval for Mean	Lower Bound	1050.84	
			Upper Bound	2754.94	
		5% Trimmed Mean		1669.04	
		Median		1230.00	
		Variance		2935687.399	
		Std. Deviation		1713.385	
		Minimum		635	
		Maximum		7380	
		Range		6745	
		Interquartile Range		875	
		Skewness		2.514	.536
		Kurtosis		6.254	1.038
CICUstay	No	Mean		2649.41	310.869
		95% Confidence Interval for Mean	Lower Bound	2015.39	
			Upper Bound	3283.43	
		5% Trimmed Mean		2516.56	
		Median		1742.00	
		Variance		3092457.926	
		Std. Deviation		1758.539	
		Minimum		750	
		Maximum		7420	
		Range		6670	
		Interquartile Range		2354	
		Skewness		1.177	.414
		Kurtosis		.463	.809
	Yes	Mean		5075.00	888.902
		95% Confidence Interval for Mean	Lower Bound	3199.58	
			Upper Bound	6950.42	
		5% Trimmed Mean		4741.67	

Descriptives

AF				Statistic	Std. Error
		Median		3847.50	
		Variance		14222626.47	
		Std. Deviation		3771.290	
		Minimum		690	
		Maximum		15460	
		Range		14770	
		Interquartile Range		4773	
		Skewness		1.259	.536
		Kurtosis		1.940	1.038
HDUstay	No	Mean		2243.59	299.573
		95% Confidence Interval for Mean	Lower Bound	1632.61	
			Upper Bound	2854.58	
		5% Trimmed Mean		2040.52	
		Median		1480.00	
		Variance		2871800.378	
		Std. Deviation		1694.639	
		Minimum		330	
		Maximum		7610	
		Range		7280	
		Interquartile Range		1514	
		Skewness		2.081	.414
		Kurtosis		4.228	.809
	Yes	Mean		2396.67	360.014
		95% Confidence Interval for Mean	Lower Bound	1637.10	
			Upper Bound	3156.23	
		5% Trimmed Mean		2319.63	
		Median		1755.00	
		Variance		2332976.471	
		Std. Deviation		1527.408	
		Minimum		405	
		Maximum		5775	
		Range		5370	
		Interquartile Range		2743	
		Skewness		.918	.536
		Kurtosis		-.328	1.038
HospStay	No	Mean		7.91	.441
		95% Confidence Interval for Mean	Lower Bound	7.01	
			Upper Bound	8.81	
		5% Trimmed Mean		7.54	
		Median		7.00	
		Variance		6.217	
		Std. Deviation		2.493	
		Minimum		6	

Descriptives

AF				Statistic	Std. Error
		Maximum		19	
		Range		13	
		Interquartile Range		3	
		Skewness		3.097	.414
		Kurtosis		12.477	.809
	Yes	Mean		9.78	.823
		95% Confidence Interval for Mean	Lower Bound	8.04	
			Upper Bound	11.51	
		5% Trimmed Mean		9.36	
		Median		9.00	
		Variance		12.183	
		Std. Deviation		3.490	
		Minimum		6	
		Maximum		21	
		Range		15	
		Interquartile Range		3	
		Skewness		2.067	.536
		Kurtosis		5.778	1.038

Tests of Normality

		Kolmogorov-Smirnov[a]			Shapiro-Wilk		
	AF	Statistic	df	Sig.	Statistic	df	Sig.
Total hours intubated	No	.320	32	.000	.446	32	.000
	Yes	.334	18	.000	.628	18	.000
CICUstay	No	.215	32	.001	.843	32	.000
	Yes	.187	18	.097	.888	18	.036
HDUstay	No	.300	32	.000	.712	32	.000
	Yes	.292	18	.000	.861	18	.013
HospStay	No	.236	32	.000	.667	32	.000
	Yes	.252	18	.004	.791	18	.001

a. Lilliefors Significance Correction

Total hours intubated

Stem-and-Leaf Plots

```
Total hours intubated Stem-and-Leaf Plot for
AF= No

 Frequency    Stem &  Leaf

     3.00        8 .  688
```

```
     1.00        9 .  0
     2.00        9 .  79
     2.00       10 .  02
     4.00       10 .  7889
     4.00       11 .  2334
     1.00       11 .  7
     1.00       12 .  0
     4.00       12 .  9999
      .00       13 .
     2.00       13 .  56
     1.00       14 .  4
     2.00       14 .  67
      .00       15 .
      .00       15 .
     1.00       16 .  4
     4.00 Extremes    (>=2048)

 Stem width:       100
 Each leaf:        1 case(s)
```

```
Total hours intubated Stem-and-Leaf Plot for
AF= Yes

 Frequency    Stem &  Leaf

     2.00        0 .  69
    11.00        1 .  00001122344
     1.00        1 .  6
      .00        2 .
     1.00        2 .  6
     3.00 Extremes    (>=2750)

 Stem width:      1000
 Each leaf:        1 case(s)
```

Normal Q-Q Plots

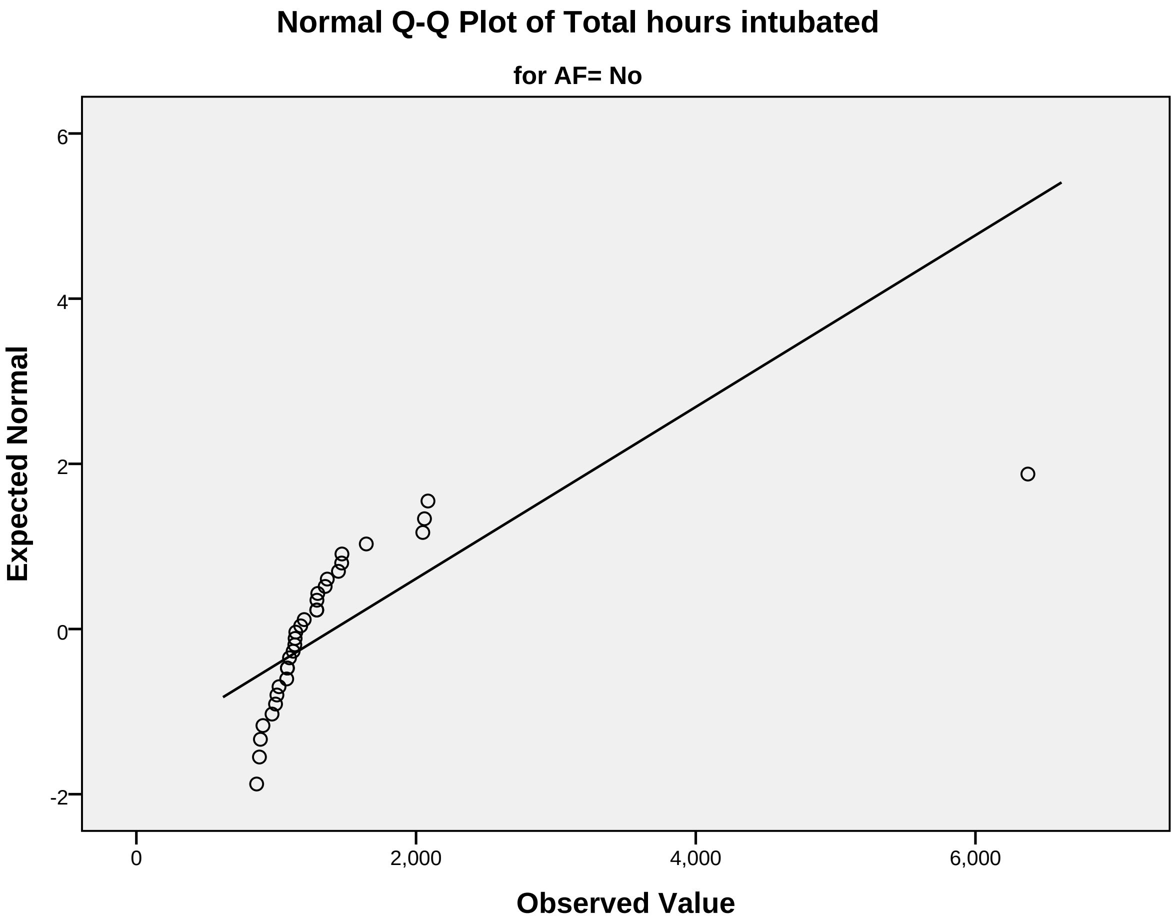

Normal Q-Q Plot of Total hours intubated
for AF= No
Expected Normal
6
4
2
0
-2
0
2,000
4,000
6,000
Observed Value

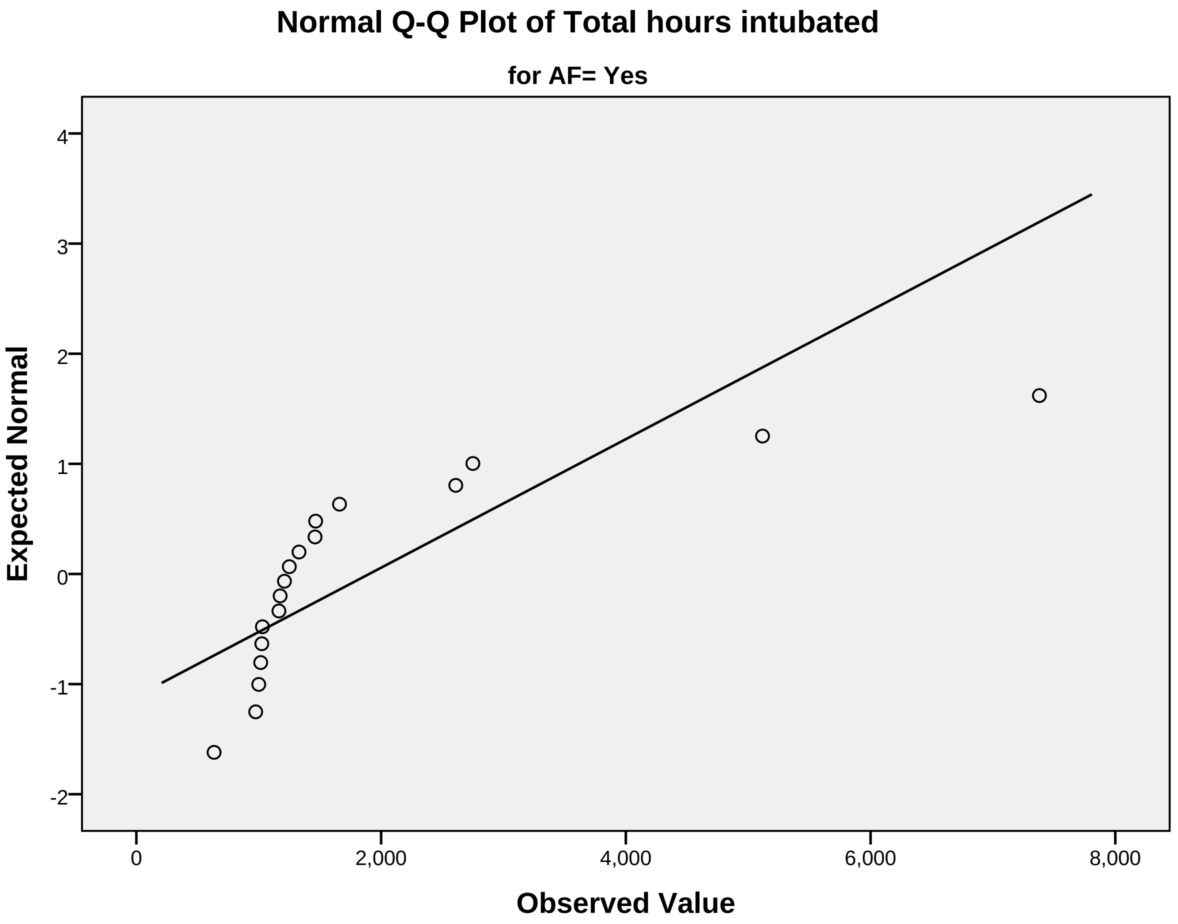

Detrended Normal Q-Q Plots

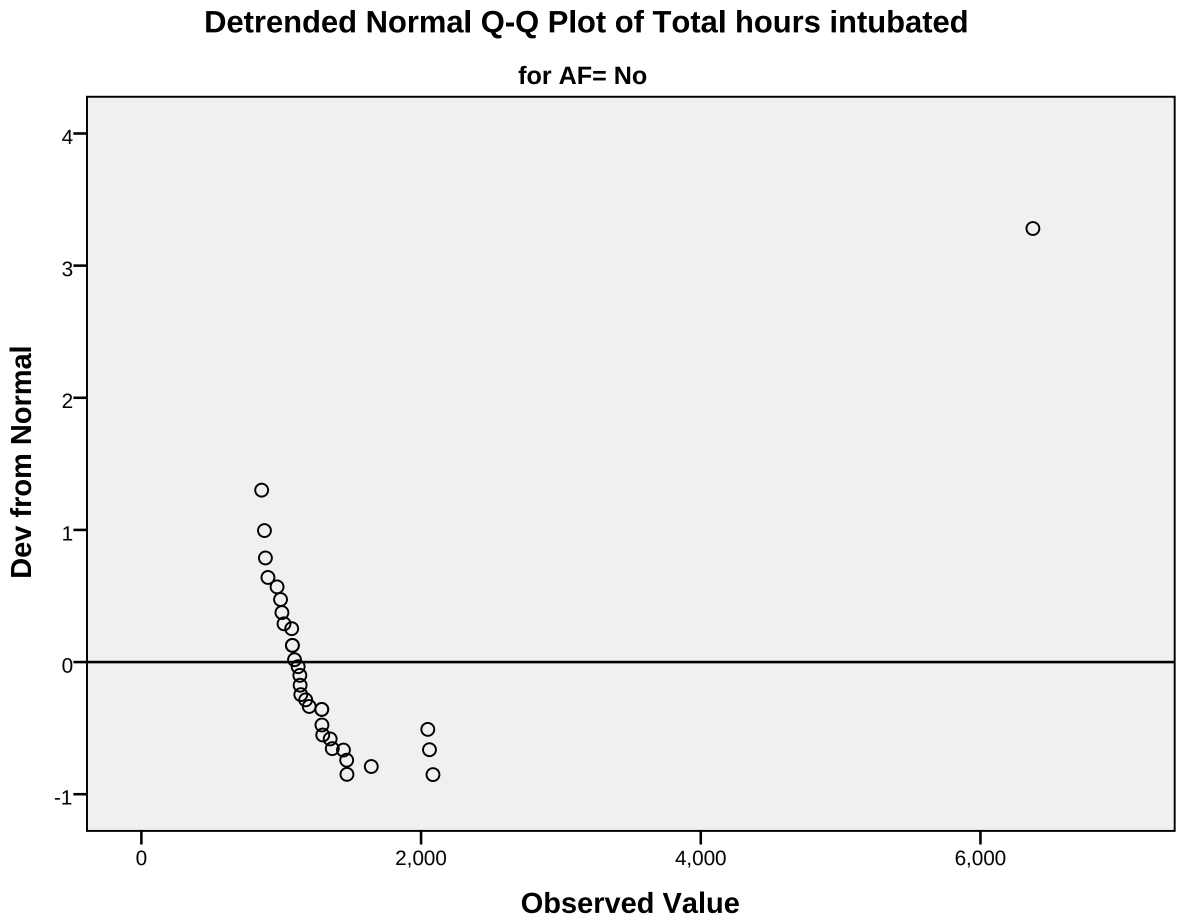
Detrended Normal Q-Q Plot of Total hours intubated
for AF= No
Dev from Normal
4
3
2
1
0
-1
0
2,000
4,000
6,000
Observed Value

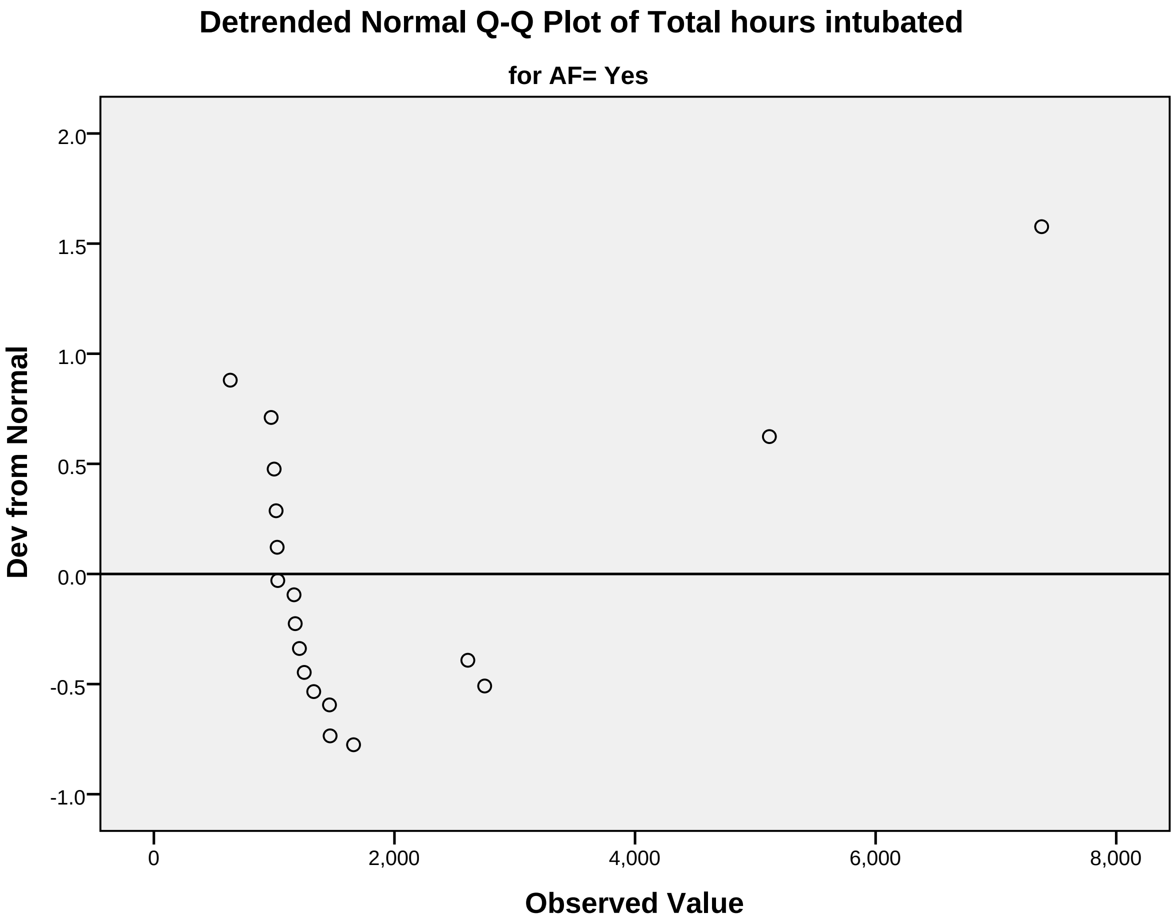
Detrended Normal Q-Q Plot of Total hours intubated
for AF= Yes
Dev from Normal
2.0
1.5
1.0
0.5
0.0
-0.5
-1.0
0
2,000
4,000
6,000
8,000
Observed Value

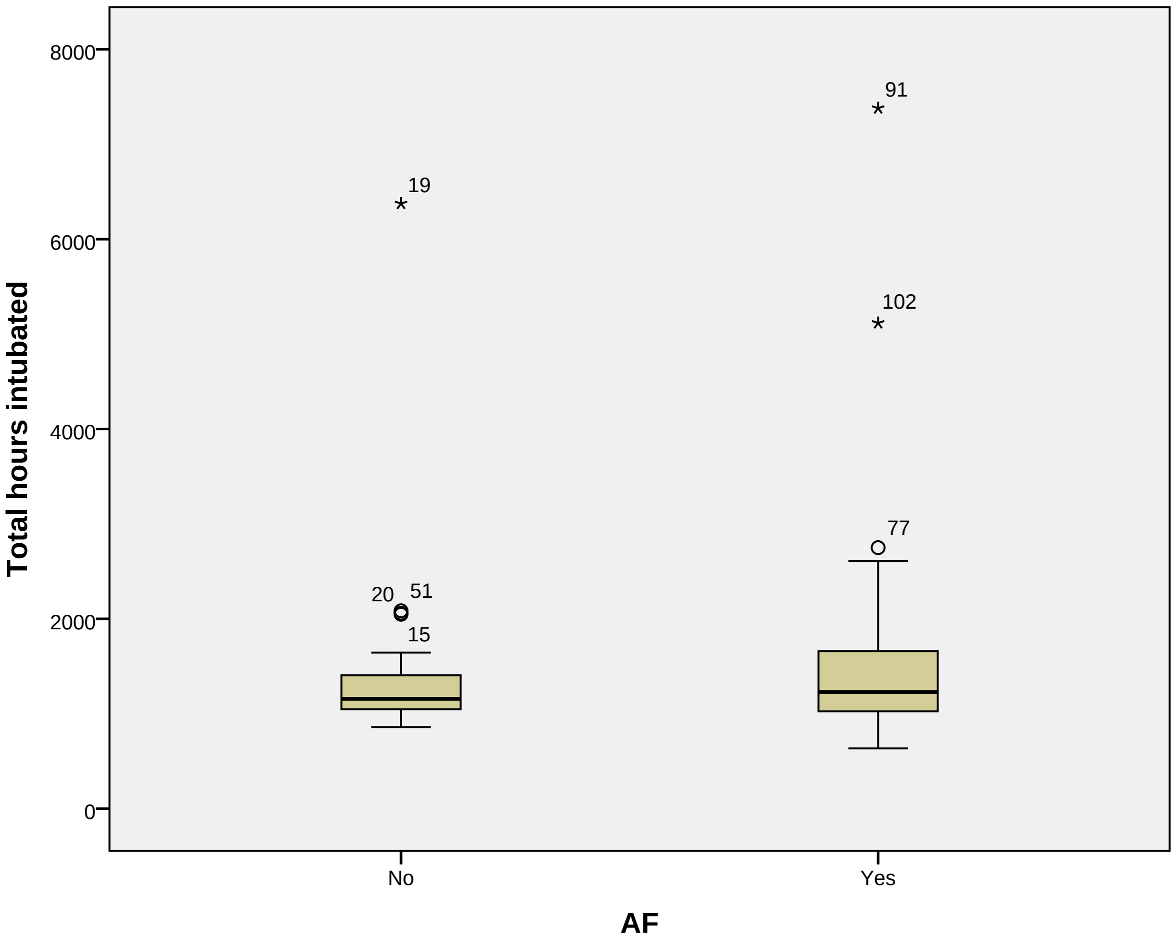

CICUstay

Stem-and-Leaf Plots

```
CICUstay Stem-and-Leaf Plot for
AF= No

 Frequency    Stem &  Leaf

     2.00        0 .  79
    10.00        1 .  0222333444
     5.00        1 .  55668
     1.00        2 .  1
     5.00        2 .  77888
     1.00        3 .  0
     2.00        3 .  89
      .00        4 .
     1.00        4 .  6
     2.00        5 .  34
     2.00        5 .  69
     1.00 Extremes    (>=7420)
```

```
 Stem width:      1000
 Each leaf:        1 case(s)
```

```
CICUstay Stem-and-Leaf Plot for
AF= Yes

 Frequency    Stem &  Leaf

     1.00        0 .  6
     3.00        1 .  157
     4.00        2 .  5789
     1.00        3 .  2
     1.00        4 .  4
     1.00        5 .  6
     1.00        6 .  7
     3.00        7 .  001
     1.00        8 .  4
     1.00        9 .  9
     1.00 Extremes    (>=15460)

 Stem width:      1000
 Each leaf:        1 case(s)
```

Normal Q-Q Plots

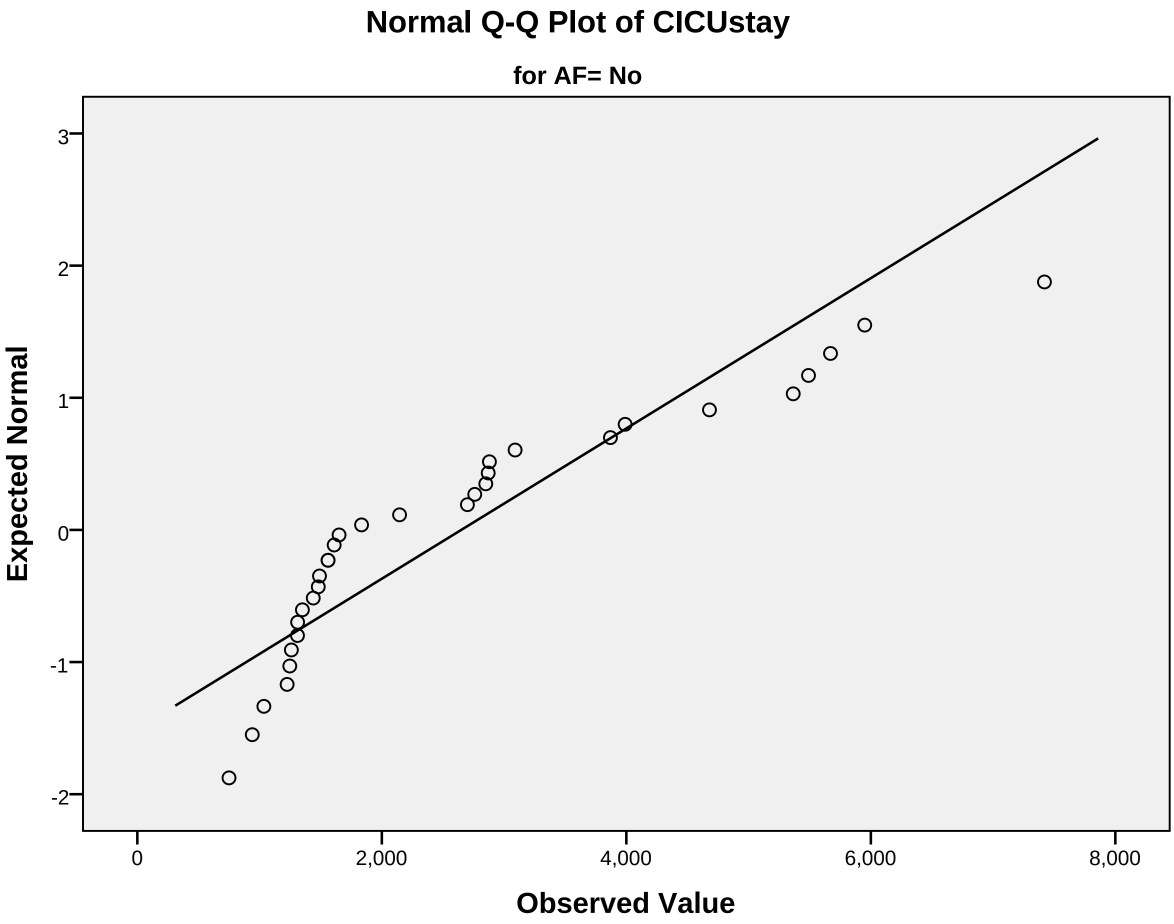
Normal Q-Q Plot of CICUstay
for AF= No
Expected Normal
3
2
1
0
-1
-2
0
2,000
4,000
6,000
8,000
Observed Value

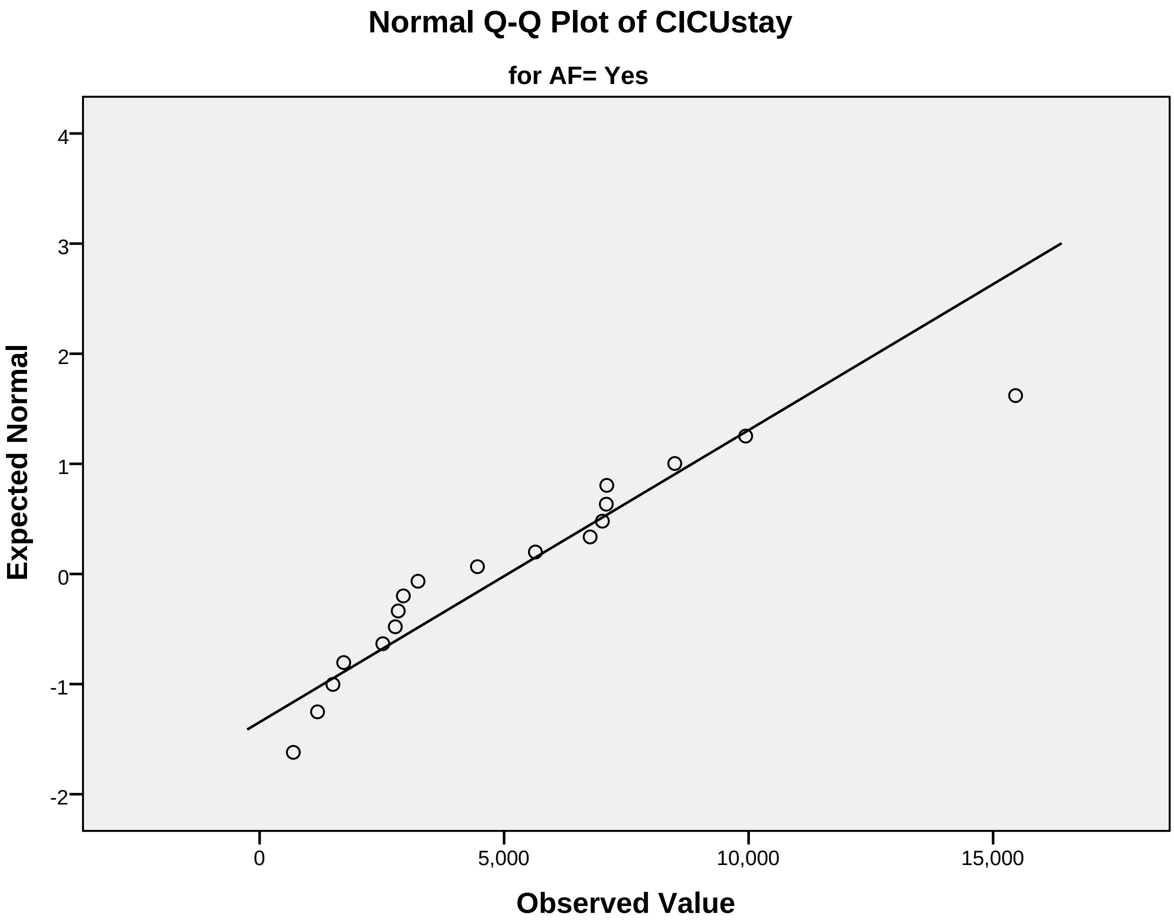

Detrended Normal Q-Q Plots

Detrended Normal Q-Q Plot of CICUstay

for AF= No

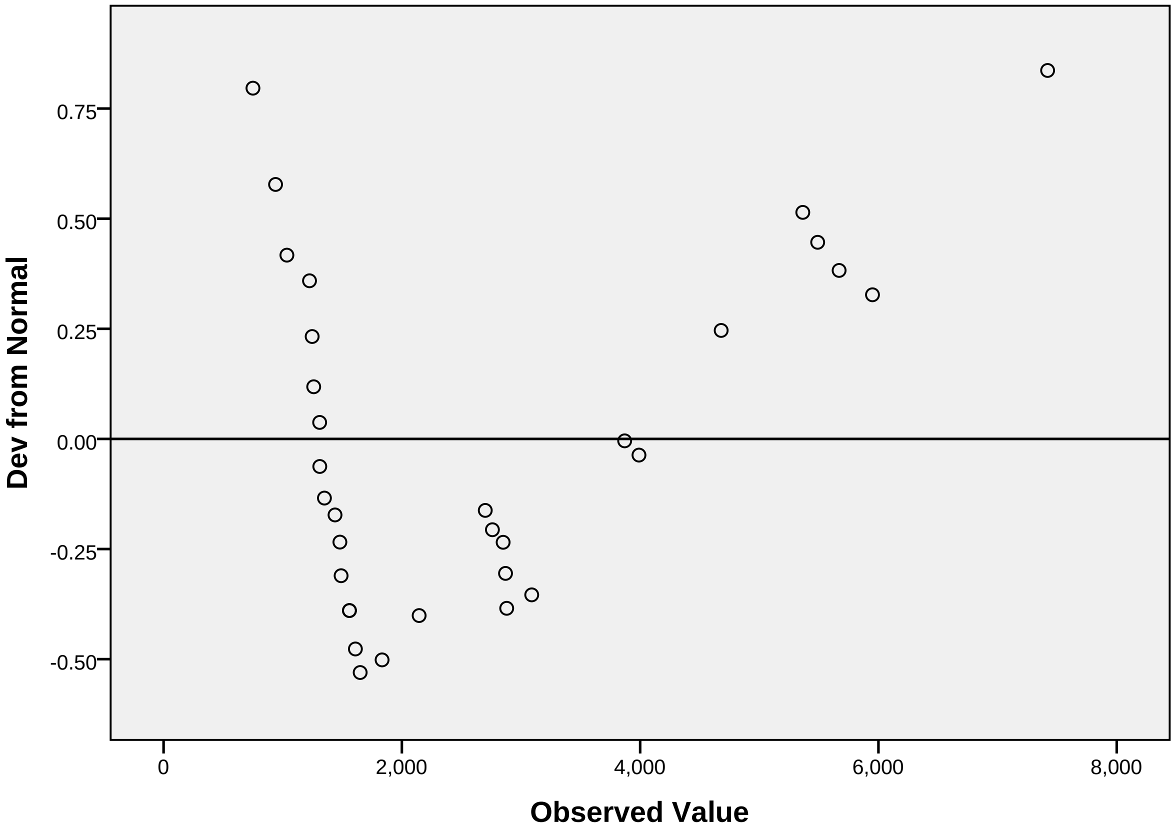

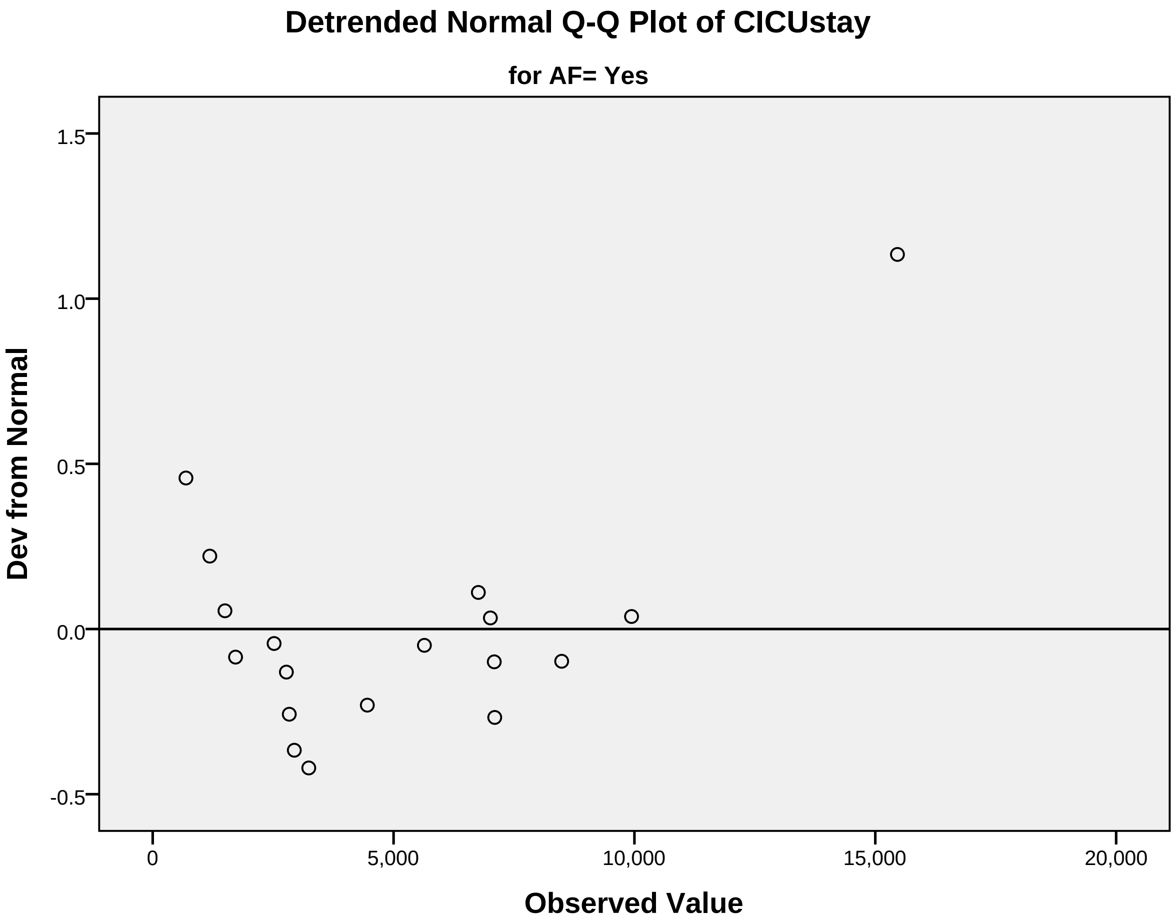
Detrended Normal Q-Q Plot of CICUstay
for AF= Yes
Dev from Normal
1.5
1.0
0.5
0.0
-0.5
0
5,000
10,000
15,000
20,000
Observed Value

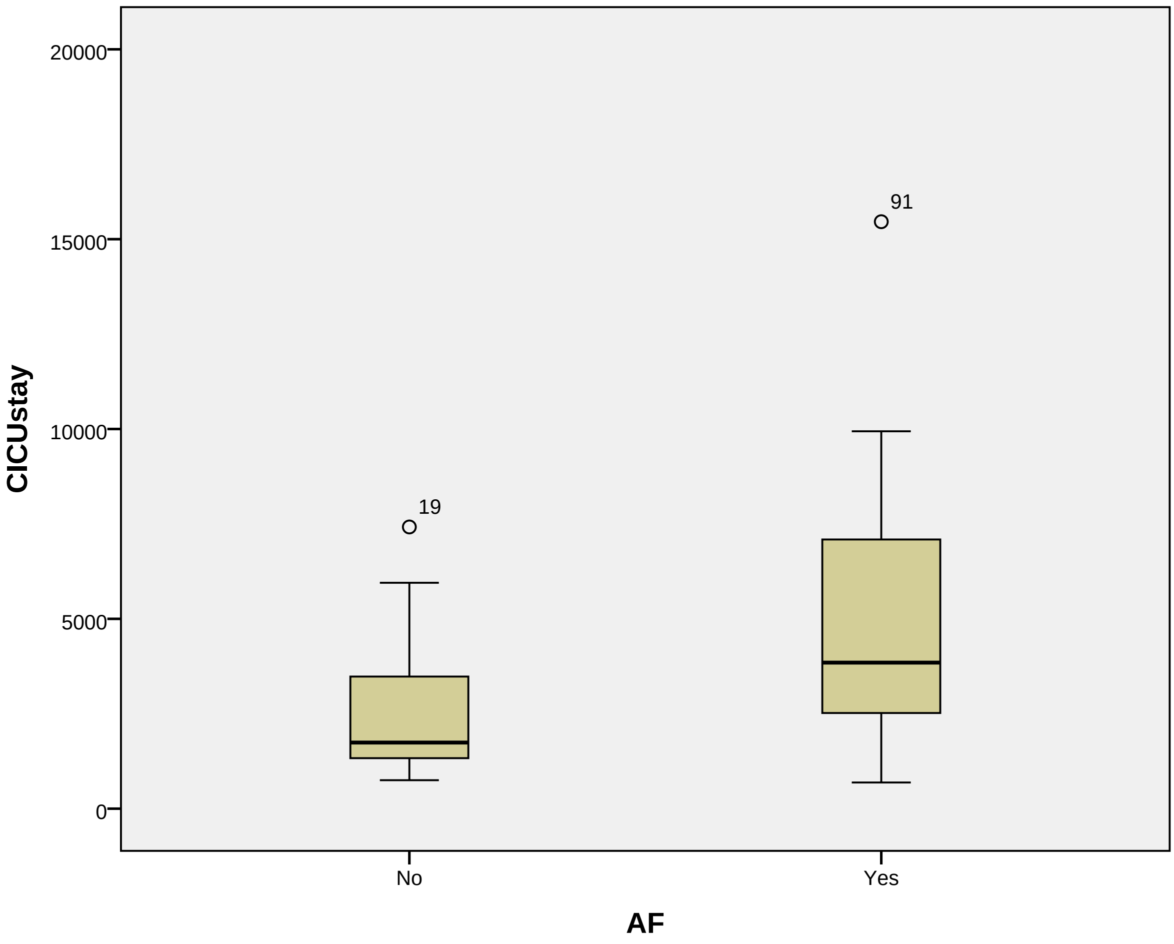

HDUstay

Stem-and-Leaf Plots

```
HDUstay Stem-and-Leaf Plot for
AF= No

 Frequency    Stem &  Leaf

     1.00        0 .  3
      .00        0 .
    16.00        1 .  1112223333344444
     5.00        1 .  55569
      .00        2 .
     4.00        2 .  5789
     1.00        3 .  1
      .00        3 .
     3.00        4 .  234
     2.00 Extremes    (>=7340)

 Stem width:      1000
```

```
 Each leaf:        1 case(s)



HDUstay Stem-and-Leaf Plot for
AF= Yes

 Frequency    Stem &  Leaf

     1.00        0 .  4
     1.00        0 .  9
     4.00        1 .  0344
     6.00        1 .  566889
      .00        2 .
     1.00        2 .  9
      .00        3 .
      .00        3 .
     3.00        4 .  112
     1.00        4 .  6
      .00        5 .
     1.00        5 .  7

 Stem width:      1000
 Each leaf:        1 case(s)
```

Normal Q-Q Plots

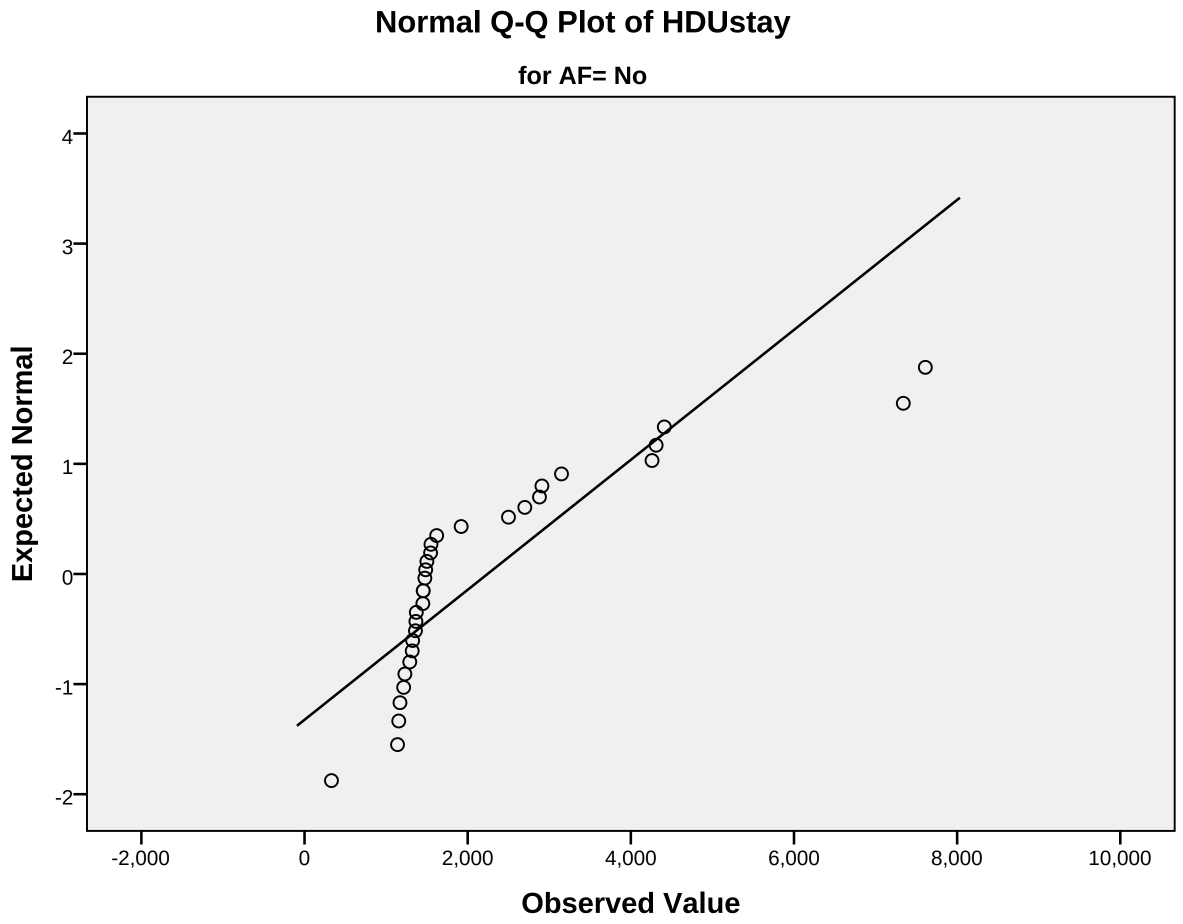
Normal Q-Q Plot of HDUstay
for AF= No
Expected Normal
4
3
2
1
0
-1
-2
-2,000
0
2,000
4,000
6,000
8,000
10,000
Observed Value

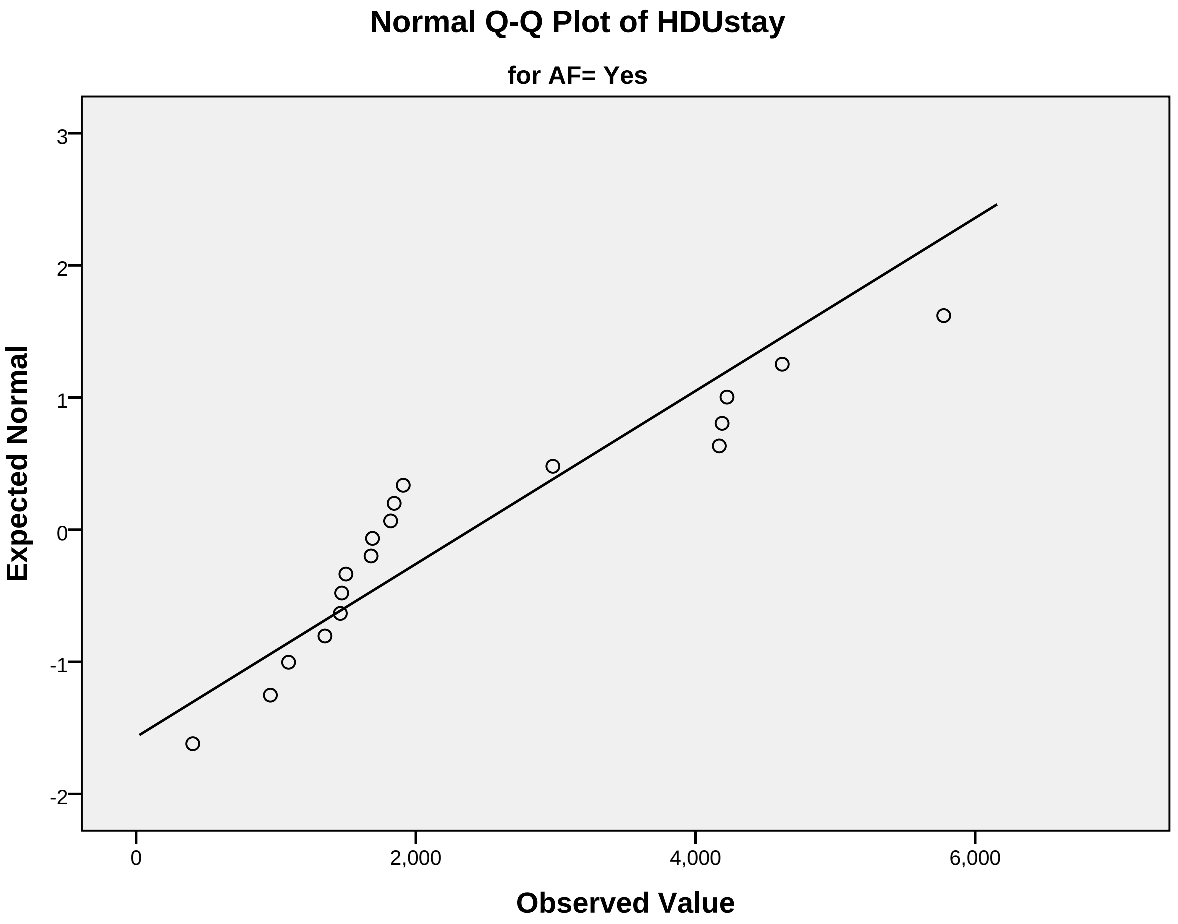

Detrended Normal Q-Q Plots

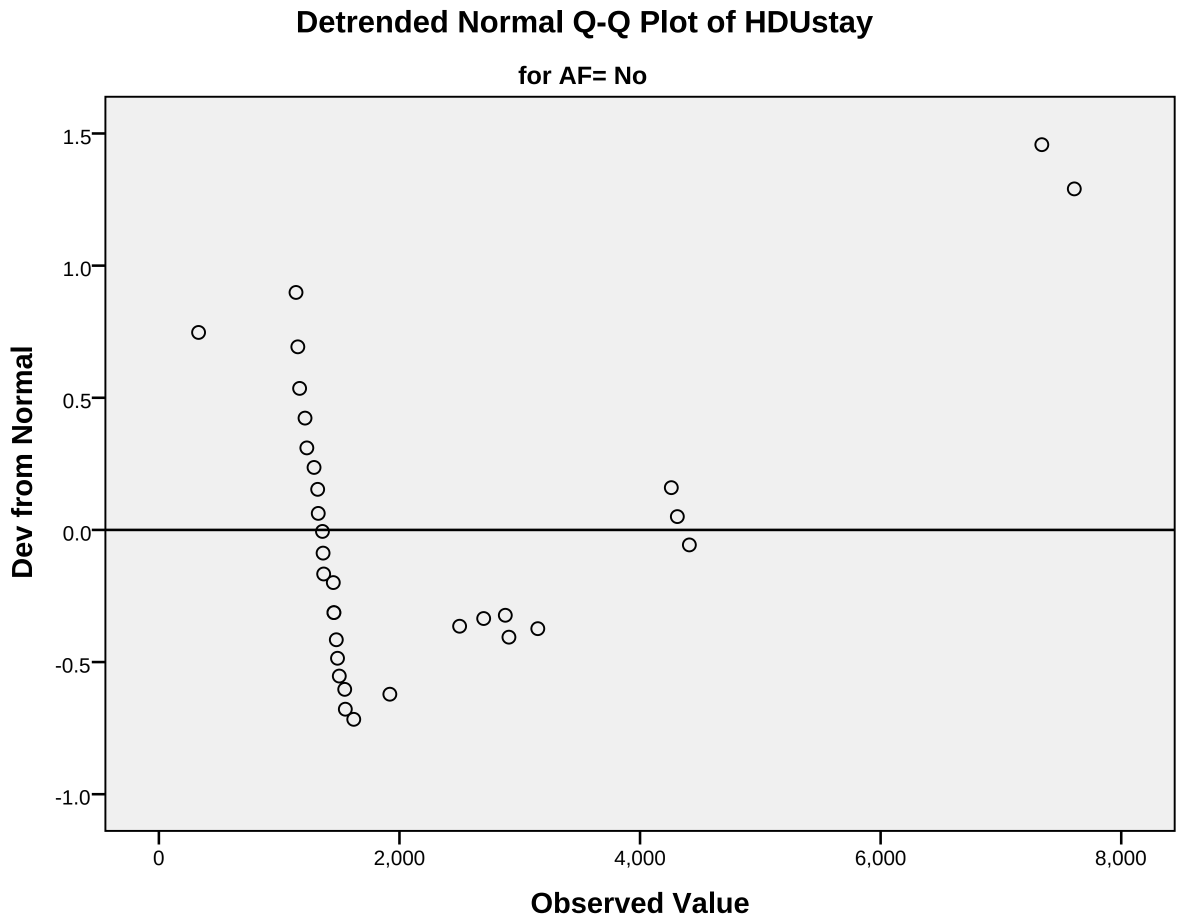

Detrended Normal Q-Q Plot of HDUstay
for AF= No
Dev from Normal
1.5
1.0
0.5
0.0
-0.5
-1.0
0
2,000
4,000
6,000
8,000
Observed Value

Detrended Normal Q-Q Plot of HDUstay

for AF= Yes

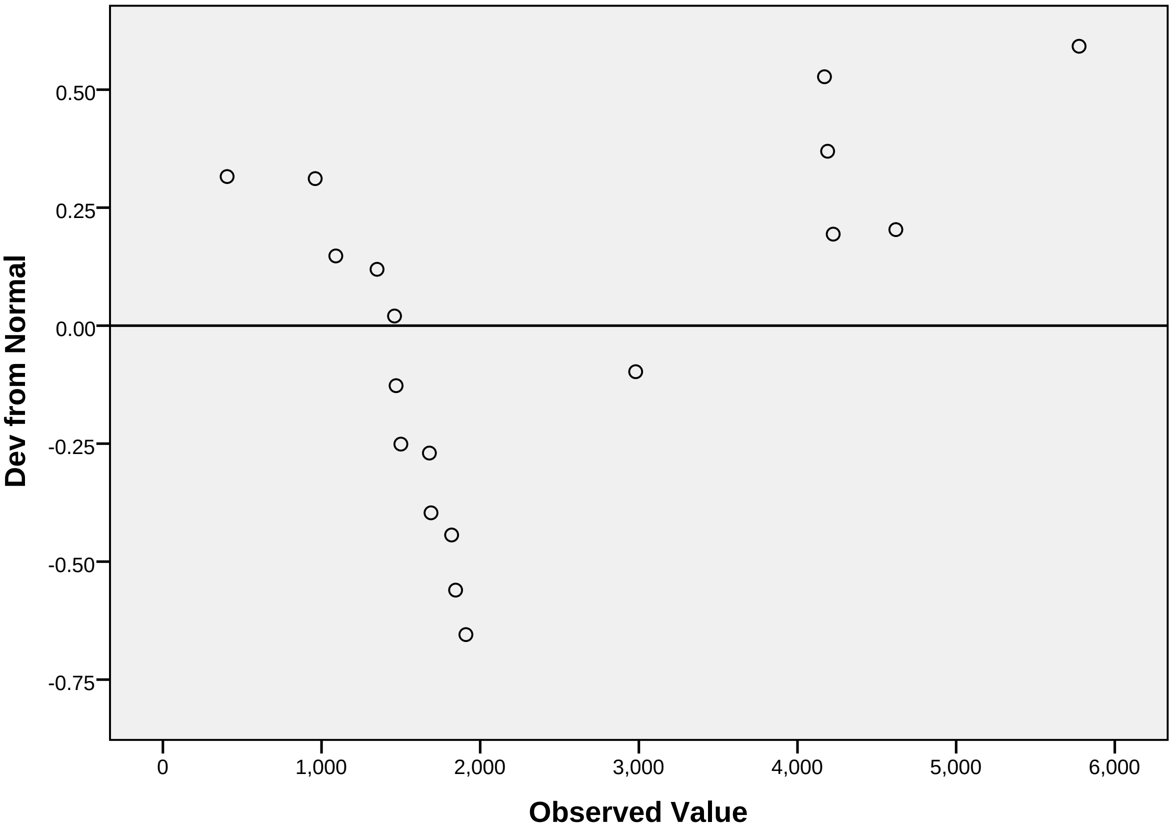

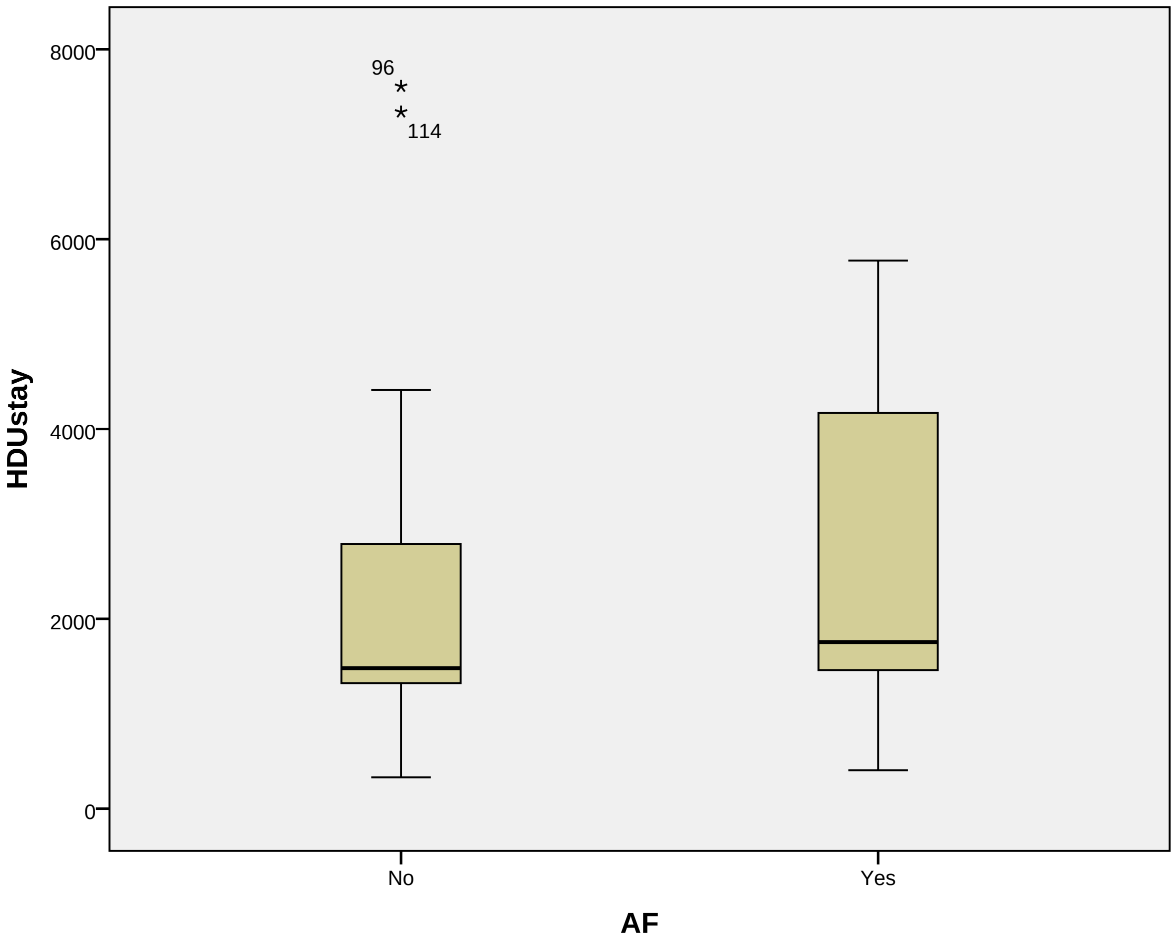

HospStay

Stem-and-Leaf Plots

```
HospStay Stem-and-Leaf Plot for
AF= No

 Frequency    Stem &  Leaf

     8.00        6 .  00000000
      .00        6 .
    11.00        7 .  00000000000
      .00        7 .
     4.00        8 .  0000
      .00        8 .
     5.00        9 .  00000
      .00        9 .
     2.00       10 .  00
      .00       10 .
      .00       11 .
      .00       11 .
```

```
     1.00       12 .  0
     1.00 Extremes    (>=19.0)

 Stem width:         1
 Each leaf:        1 case(s)
```

```
HospStay Stem-and-Leaf Plot for
AF= Yes

 Frequency    Stem &  Leaf

     2.00        6 .  00
     1.00        7 .  0
     6.00        8 .  000000
      .00        9 .
     5.00       10 .  00000
      .00       11 .
     1.00       12 .  0
     2.00       13 .  00
     1.00 Extremes    (>=21.0)

 Stem width:         1
 Each leaf:        1 case(s)
```

Normal Q-Q Plots

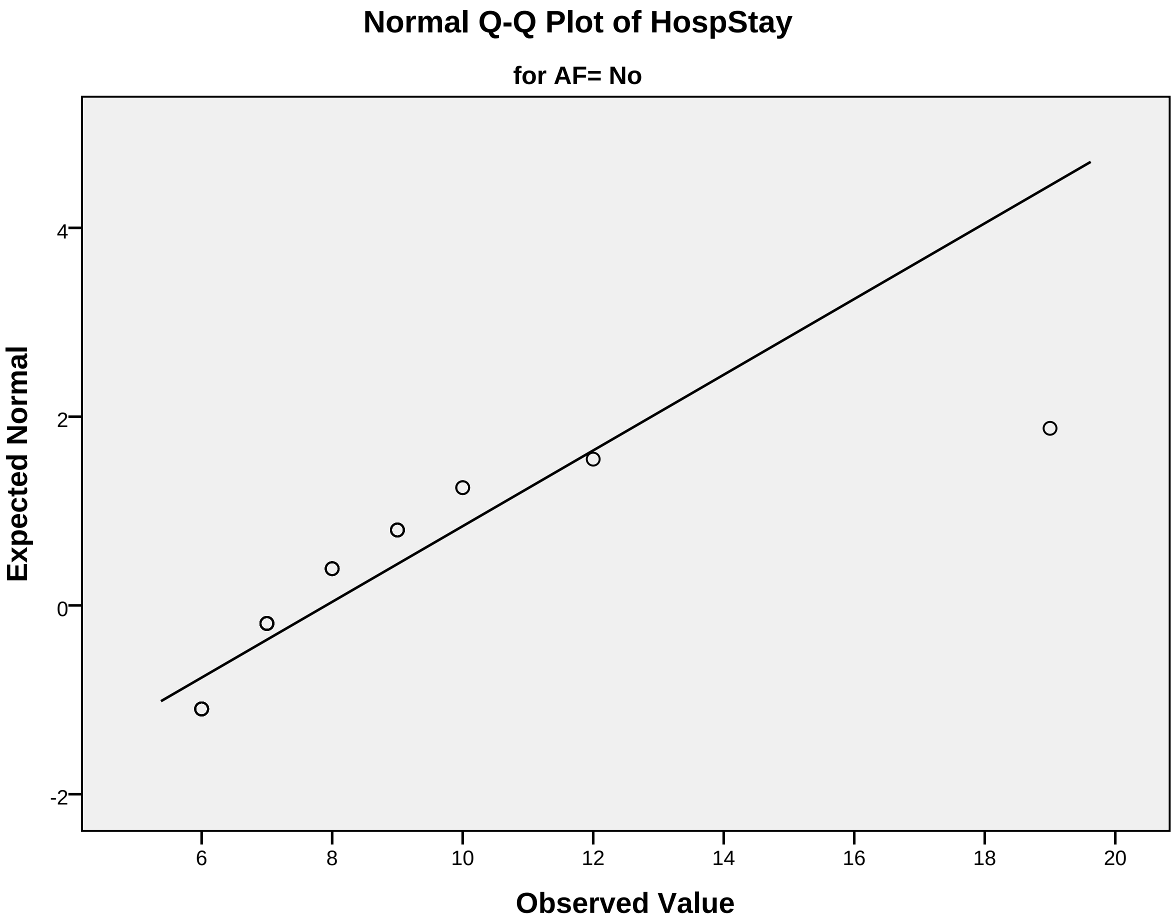
Normal Q-Q Plot of HospStay
for AF= No
Expected Normal
4
2
0
-2
6
8
10
12
14
16
18
20
Observed Value

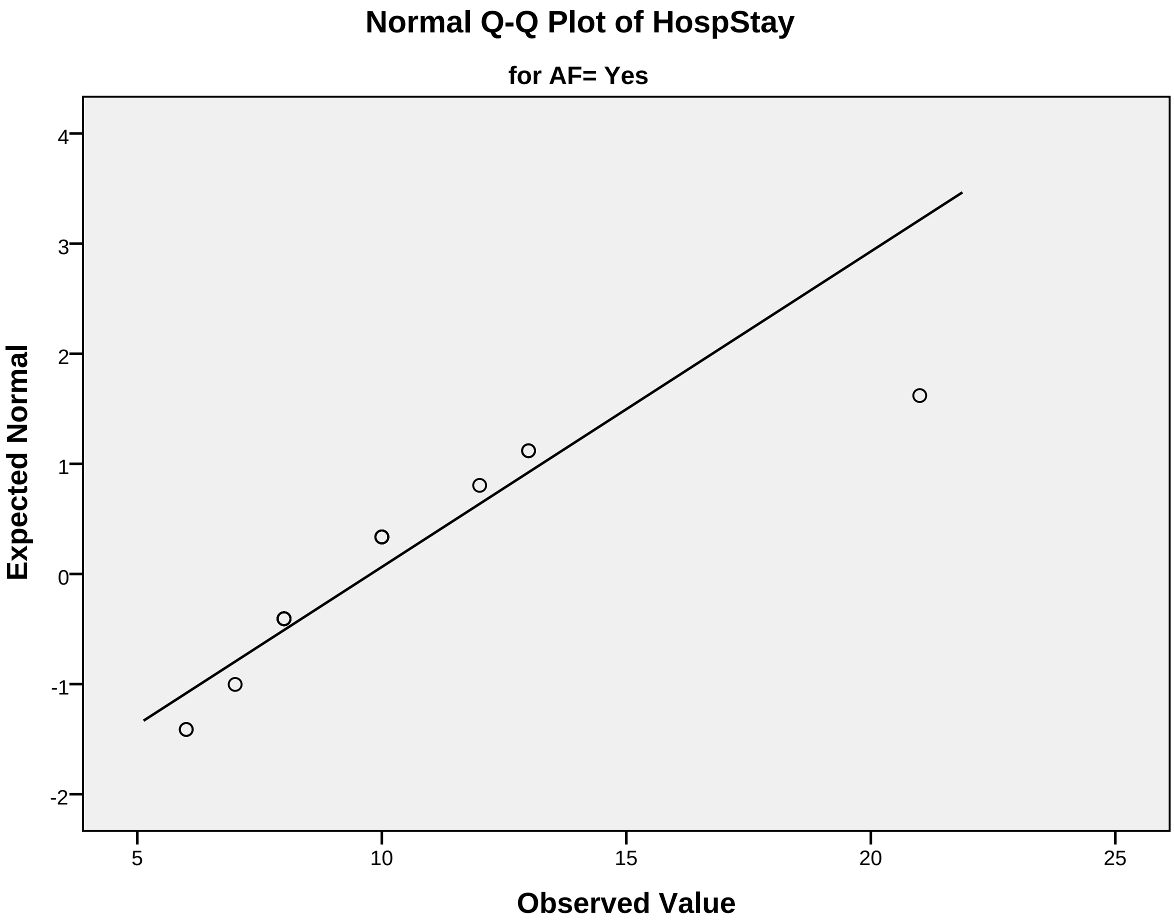

Detrended Normal Q-Q Plots

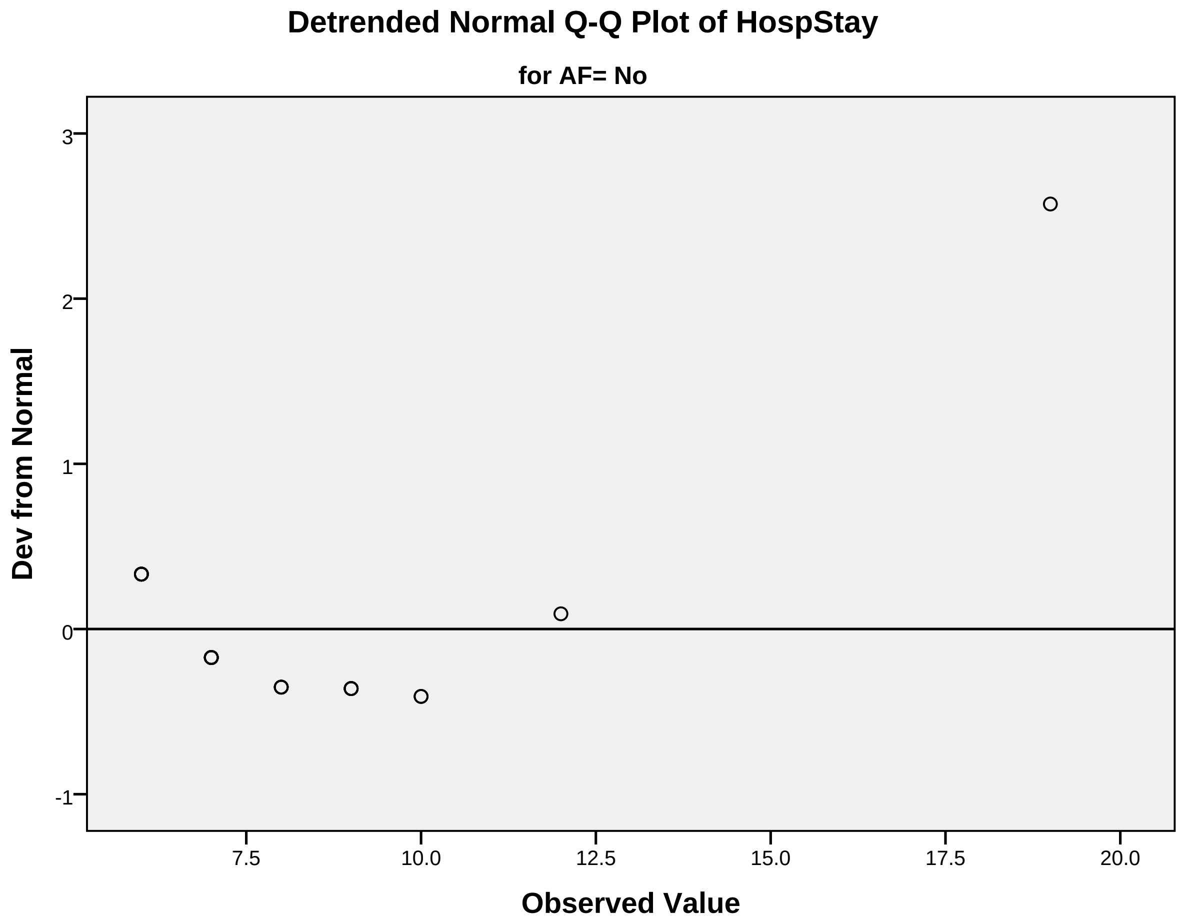
Detrended Normal Q-Q Plot of HospStay
for AF= No
Dev from Normal
3
2
1
0
-1
7.5
10.0
12.5
15.0
17.5
20.0
Observed Value

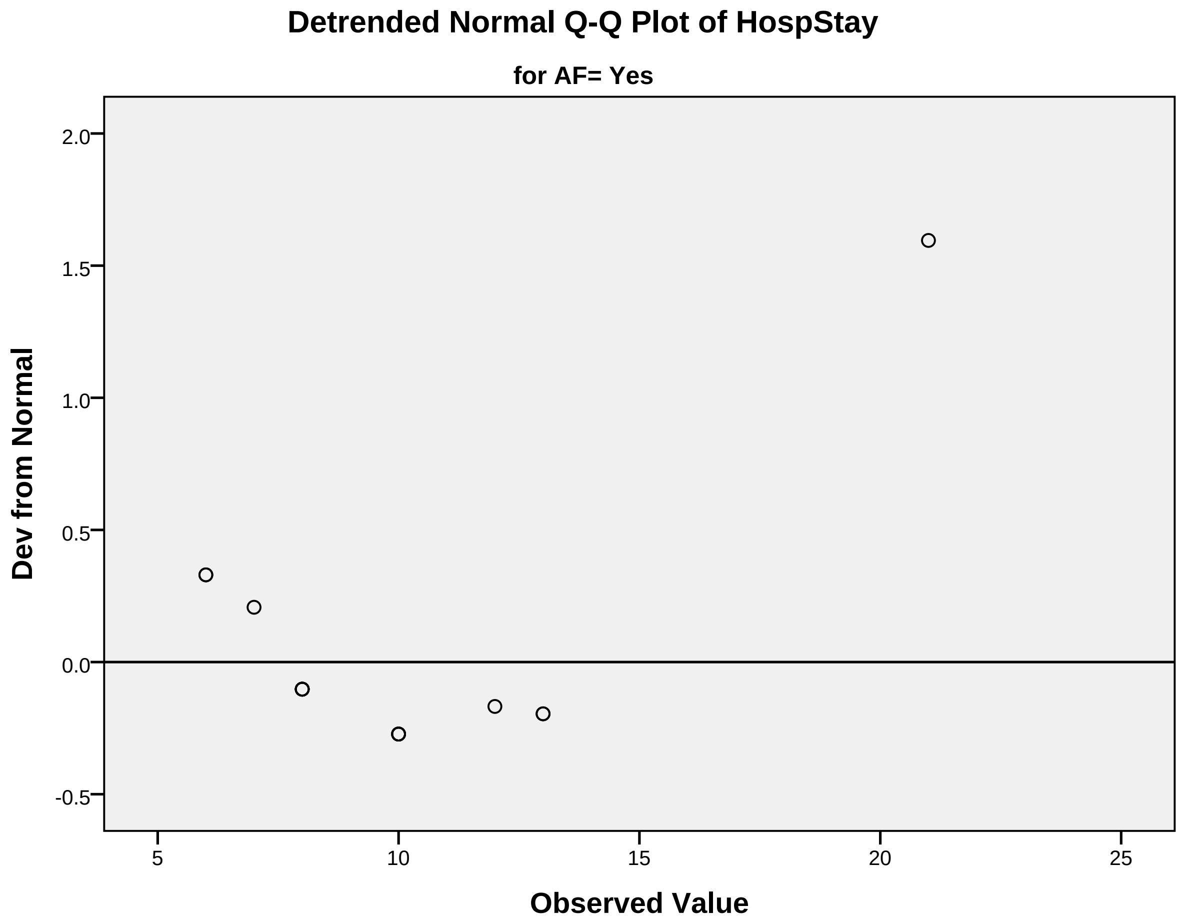
Detrended Normal Q-Q Plot of HospStay
for AF= Yes
Dev from Normal
2.0
1.5
1.0
0.5
0.0
-0.5
5
10
15
20
25
Observed Value

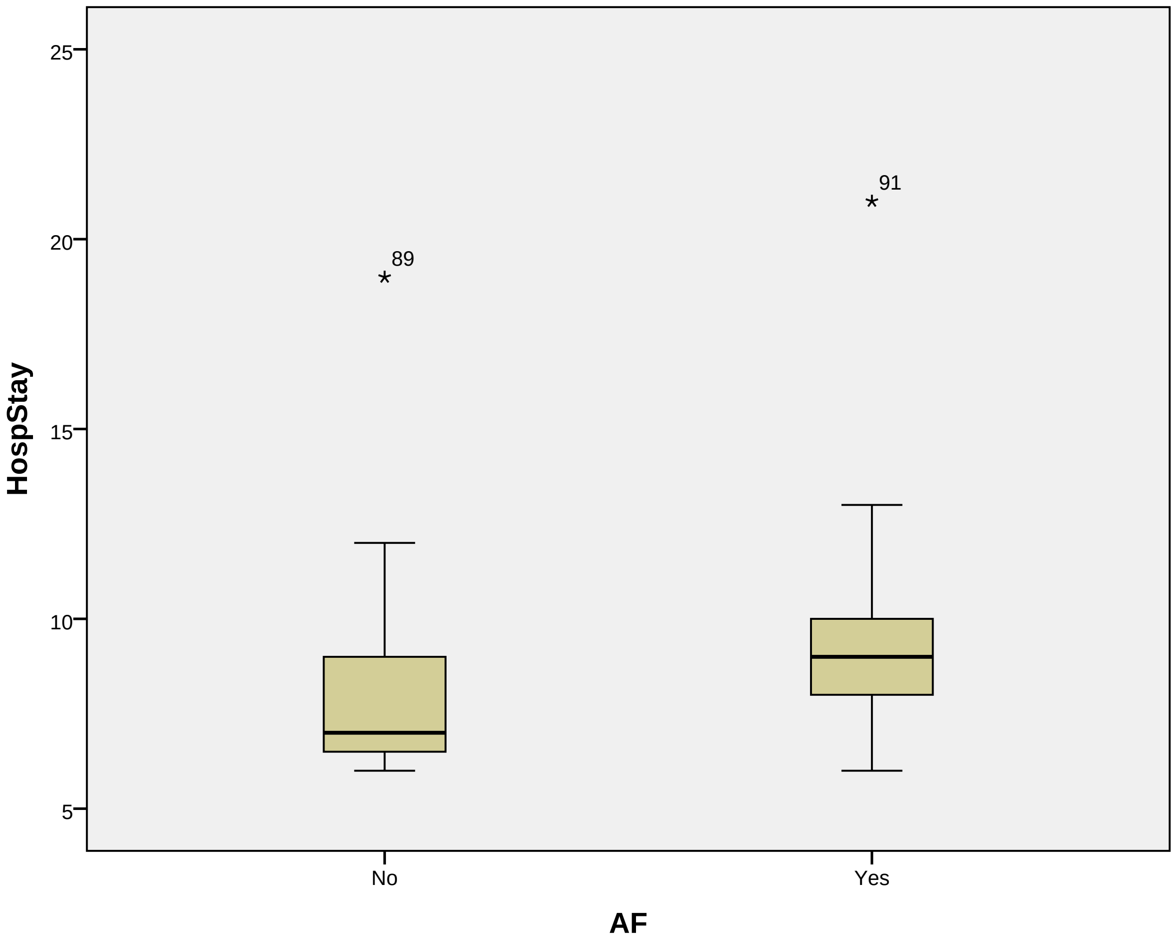


```
NPAR TESTS
 /M-W= VENT CICUstay HDUstay HospStay BY AF(0 1)
 /STATISTICS=DESCRIPTIVES
 /MISSING ANALYSIS.
```

NPar Tests

Notes

Output Created		01-MAR-2021 14:56:23
Comments		
Input	Data	C:\Users\user\Downloads\ImAnisah Statistic & SPSS\TOCO T3\Data toco.sav
	Active Dataset	DataSet1
	Filter	<none>
	Weight	<none>
	Split File	<none>
	N of Rows in Working Data File	156
Missing Value Handling	Definition of Missing	User-defined missing values are treated as missing.
	Cases Used	Statistics for each test are based on all cases with valid data for the variable(s) used in that test.
Syntax		NPAR TESTS<br>/M-W= VENT CICUstay HDUstay HospStay BY AF(0 1)<br>/STATISTICS=DESCRIPTIVES<br>/MISSING ANALYSIS.
Resources	Processor Time	00:00:00.00
	Elapsed Time	00:00:00.02
	Number of Cases Allowed[a]	314572

a. Based on availability of workspace memory.

Descriptive Statistics

	N	Mean	Std. Deviation	Minimum	Maximum
Total hours intubated	101	1421.36	1053.102	619	7380
CICUstay	99	3505.67	3777.435	640	30405
HDUstay	54	2274.44	1574.645	330	7610
HospStay	135	10.81	11.597	5	86
AF	156	.24	.431	0	1

Mann-Whitney Test

Ranks

	AF	N	Mean Rank	Sum of Ranks
Total hours intubated	No	72	48.74	3509.00
	Yes	29	56.62	1642.00
	Total	101		
CICUstay	No	72	45.42	3270.00
	Yes	27	62.22	1680.00
	Total	99		
HDUstay	No	33	25.83	852.50
	Yes	21	30.12	632.50
	Total	54		
HospStay	No	101	64.03	6467.50
	Yes	34	79.78	2712.50
	Total	135		

Test Statistics[a]

	Total hours intubated	CICUstay	HDUstay	HospStay
Mann-Whitney U	881.000	642.000	291.500	1316.500
Wilcoxon W	3509.000	3270.000	852.500	6467.500
Z	-1.224	-2.593	-.976	-2.060
Asymp. Sig. (2-tailed)	.221	.010	.329	.039

a. Grouping Variable: AF

```
CROSSTABS
 /TABLES=Reintubation BY AF
 /FORMAT=AVALUE TABLES
 /STATISTICS=CHISQ
 /CELLS=COUNT ROW COLUMN
 /COUNT ROUND CELL.
```

Crosstabs

Notes

Output Created		01-MAR-2021 14:57:09
Comments		
Input	Data	C:\Users\user\Downloads\ImAnisah Statistic & SPSS\TOCO T3\Data toco.sav
	Active Dataset	DataSet1
	Filter	<none>
	Weight	<none>
	Split File	<none>
	N of Rows in Working Data File	156
Missing Value Handling	Definition of Missing	User-defined missing values are treated as missing.
	Cases Used	Statistics for each table are based on all the cases with valid data in the specified range(s) for all variables in each table.
Syntax		CROSSTABS<br>/TABLES=Reintubation BY AF<br>/FORMAT=AVALUE TABLES<br>/STATISTICS=CHISQ<br>/CELLS=COUNT ROW COLUMN<br>/COUNT ROUND CELL.
Resources	Processor Time	00:00:00.03
	Elapsed Time	00:00:00.05
	Dimensions Requested	2
	Cells Available	524245

Case Processing Summary

	Cases					
	Valid		Missing		Total	
	N	Percent	N	Percent	N	Percent
Reintubation * AF	156	100.0%	0	0.0%	156	100.0%

Reintubation * AF Crosstabulation

			AF		Total
			No	Yes	
Reintubation	No	Count	117	35	152
		% within Reintubation	77.0%	23.0%	100.0%
		% within AF	99.2%	92.1%	97.4%
	Yes	Count	1	3	4
		% within Reintubation	25.0%	75.0%	100.0%
		% within AF	0.8%	7.9%	2.6%
Total		Count	118	38	156
		% within Reintubation	75.6%	24.4%	100.0%
		% within AF	100.0%	100.0%	100.0%

Chi-Square Tests

	Value	df	Asymptotic Significance (2-sided)	Exact Sig. (2-sided)	Exact Sig. (1-sided)
Pearson Chi-Square	5.714[a]	1	.017		
Continuity Correction[b]	3.241	1	.072		
Likelihood Ratio	4.682	1	.030		
Fisher's Exact Test				.045	.045
Linear-by-Linear Association	5.677	1	.017		
N of Valid Cases	156				

a. 2 cells (50.0%) have expected count less than 5. The minimum expected count is .97.

b. Computed only for a 2x2 table
